# Nickel-Catalyzed Three-Component 1,2-Carboacylation of Alkenes

**DOI:** 10.3390/molecules29184295

**Published:** 2024-09-10

**Authors:** Shengzhou Jin, Lanfen Wang, Yinggang Jia, Wenbo Ma, Dingyi Wang

**Affiliations:** 1 College of Sciences, Northeastern University, Shenyang 110004, China; 2 Antibiotics Research and Re-Evaluation Key Laboratory of Sichuan Province, Sichuan Industrial Institute of Antibiotics, National Base for International Science and Technology Cooperation of Chengdu University, Chengdu University, Chengdu 610106, China; 3 Hubei Key Laboratory of Pollutant Analysis & Reuse Technology, College of Chemistry and Chemical Engineering, Hubei Normal University, Huangshi 435002, China

**Keywords:** nickel-catalyzed, carboacylation, alkenes, acyl chlorides, alkyl bromides

## Abstract

Ketones, prevalent in many biologically significant molecules, require the development of novel methods to synthesize these structures, which is a critical endeavor in organic synthesis. Transition metal catalysis has proven to be an effective method for synthesizing ketones. However, the scope of these substrates remains relatively limited, particularly due to their incompatibility with sensitive functional groups. Herein, we report a Ni-catalyzed three-component 1,2-carboacylation of alkenes, which activates secondary/tertiary alkyl bromides. This method offers significant advantages: simplicity of operation, ready availability of substrates, and broad substrate applicability. A series of experimental studies have helped clarify the key mechanistic pathways involved in this cascade reaction.

## 1. Introduction

**Scheme 1:**
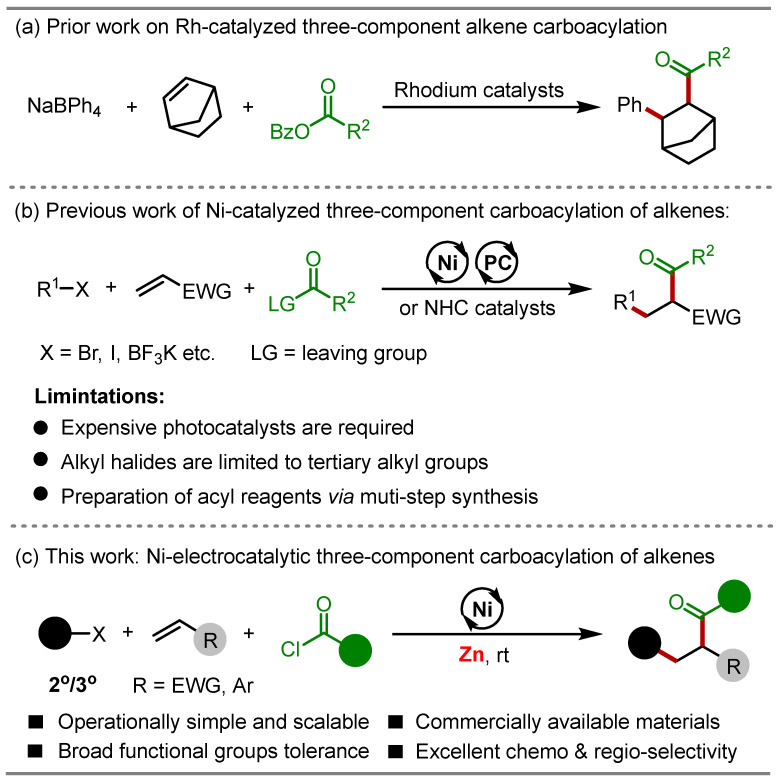
Transition-metal-catalyzed three-component 1,2-carboacylation of alkenes.

Ketones find widespread applications in pharmaceuticals, pesticides, and natural products [1,2,3,4]. Additionally, carbonyl compounds, a crucial class of organic synthesis intermediates, can undergo various transformations, including Wittig and reductive amination reactions [5,6,7]. Over the past few decades, the traditional industrial methods for the preparation of ketones have mainly involved catalytic oxidation in the presence of oxygen. However, these methods depend heavily on costly metal catalysts, stoichiometric oxidants, and high temperatures, which lead to poor functional group compatibility, substantial costs, and safety concerns, especially in large-scale production. Consequently, the development of efficient methods for synthesizing carbonyl compounds has become a critical area of research. In 2002, Miura and co-workers described a Rh-catalyzed coupling of sodium tetraphenylborate with acid anhydrides, both with and without norbornene, yielding aryl or alkyl ketones with moderate to good yields (Scheme 1a) [8]. This pioneering work demonstrated that transition-metal-catalyzed 1,2-dicarbofunctionalization via one-pot reactions is an effective method for rapidly synthesizing complex ketones by continuously introducing two functional groups into alkenes. Inspired by this work, various other transition-metal-catalyzed three-component carbonylation reactions have been documented [9,10,11,12,13,14,15,16,17,18]. Given this context and the demands of industrial production, it is imperative to develop simpler, more sustainable, and practical methods for alkene carboacylation.

Among the 3d transition metals, nickel has garnered significant attention from organic chemists due to its abundance in the Earth’s crust, non-toxic properties, and affordability [19,20,21,22,23,24,25,26,27]. Furthermore, owing to its small atomic radius, high nucleophilicity, and multiple oxidation states, nickel exhibits exceptional reactivity and selectivity in catalytic reactions [28,29,30,31,32,33,34,35]. Therefore, nickel-catalyzed carboacylation of alkenes offers unparalleled advantages over traditional synthetic methods [36,37,38,39,40,41,42,43,44,45,46,47]. In 2017, Nevado et al. first reported a nickel-catalyzed reductive three-component difunctionalization of alkenes utilizing both aryl/alkyl(pseudo)halides [41]. Subsequently, a selective intermolecular three-component alkene carboacylation was developed using Ni/photoredox dual catalysis [37]. Despite previous successes, there were notable limitations: (1) the need for costly photocatalysts; (2) the restriction of radical receptors to activated alkenes; (3) the preparation of acyl reagents via multi-step synthesis (Scheme 1b). To address these challenges, we developed a nickel-catalyzed three-component 1,2-carboacylation of alkenes that produces a variety of ketones with quaternary carbon centers. We utilized cost-effective and readily available bromo-alkanes and acyl chlorides as acylation reagents and radical precursors, respectively. This method boasts mild reaction conditions, simple operation, excellent regioselectivity and chemoselectivity, and robust functional group tolerance (Scheme 1c). Although Wangn [21] and Chu [26] also reported the 1,2-carboacylation of alkenes, their method, however, was limited to primary fluoroalkanes or tertiary bromoalkanes, whereas our reaction system is compatible with secondary haloalkanes.

## 2. Results

Motivated by this objective, we began exploring the multicomponent cascade reaction using commercially available *tert*-butyl bromides **1a**, methylacrylate **2a**, and 4-methylbenzoyl chloride **3a** as coupling agents (Table 1). We were pleased to achieve the first successful alkene difunctionalization using 10 mol% NiBr_2_•DME as the catalyst, 12 mol% 4,4-di-*tert*-butyl bipyridine (dtbbpy) **L1** as the ligand, tetrabutylammonium bromide (TBAB) as the additive, and zinc powder as the reductant in 3.0 mL of MeCN, with a 78% yield of the target product (entry 1). Systematic evaluation of additional pyridine-type ligands (**L2**–**L6**), which possess varying electronic effects, failed to enhance the yield of this transformation beyond that achieved with dtbbpy (**entries 2**–**6**). Our findings also showed that changing solvents significantly reduced the yield of the desired products (**entries 7** and **8**). The carbonyl compound was produced in low yield using 3.0 equivalents of manganese powder instead of zinc powder (**entry 9**). Finally, control experiments confirmed that this addition process was dependent on the presence of the nickel catalyst, ligand, and TBAB (**entries 10–12**). 

**Scheme 2:**
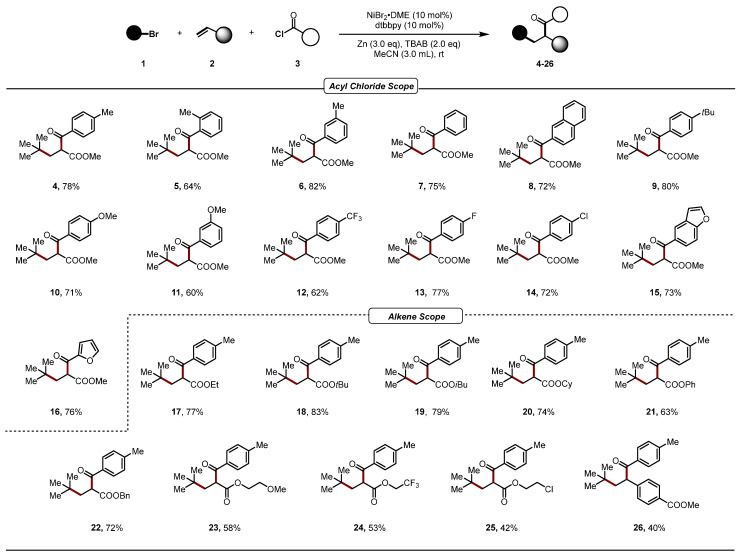
Scope of acyl chlorides and alkenes. Reaction conditions: **1** (1.0 mmol), **2** (0.2 mmol), **3** (0.6 mmol), NiBr_2_•DME (10 mol%), dtbbpy (10 mol%), TBAB (2.0 equiv.), Zn (2.0 equiv.), MeCN (3.0 mL), 12 h, rt, nitrogen, with isolated yields.

With the optimized conditions established, we evaluated the robustness of the nickel-catalyzed domino addition reaction (Scheme 2). We initially investigated the substitution patterns of aryl chlorides, discovering that various substituents at different positions on the aryl ring resulted in variable conversions. Aryl chlorides substituted with groups at the *para*- and *meta*-positions, including methyl, *tert*-butyl, methoxy, trifluoromethyl, fluorine, and chlorine, yielded products with modest to good yields (**4**–**14**). Notably, various acyl chlorides with heteroaromatic rings, including benzofurans and furans, were also well tolerated (**15** and **16**). 

Acrylic esters bearing alkyl, alkoxy, benzyl, or trifluoroethyl groups at the ester O atom yielded additional products in 42–83% yields (**17**–**22**). Moreover, this transformation also accommodated the more challenging alkyl chloride substituents in acrylic esters. Additionally, electron-deficient substituted styrenes, such as 4-ethenylbenzoic acid methyl ester, reacted to produce the target products (**26**) in modest yields. We think it may have a certain relationship with the steric hindrance of the substrate, and some other possible side reactions will also affect the yield.

**Scheme 3:**
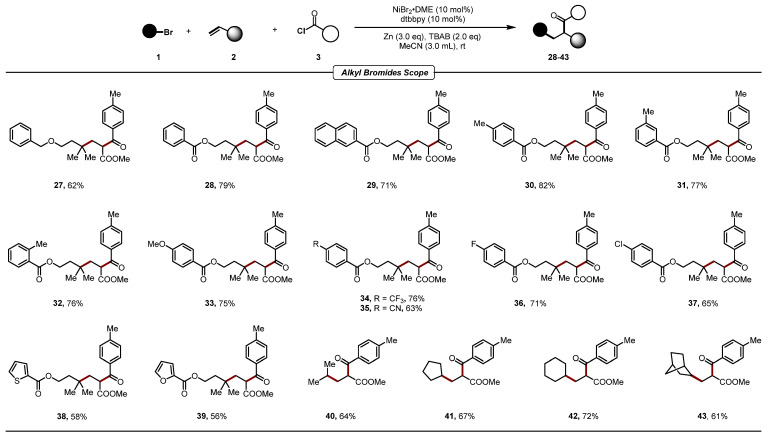
Scope of alkyl bromides. Reaction conditions: **1** (1.0 mmol), **2** (0.2 mmol), **3** (0.6 mmol), NiBr_2_•DME (10 mol%), dtbbpy (10 mol%), TBAB (2.0 equiv.), Zn (2.0 equiv.), MeCN (3.0 mL), 12 h, rt, nitrogen, with isolated yields.

Subsequently, the substrate scope with substituted alkyl bromides was explored (Scheme 3). Tertiary alkyl bromides, featuring groups like methyl, methoxy, benzyl, halogen, trifluoromethyl, cyano, thiophene, and furan, were feasible and generated products **27**–**39** in good yields with excellent site selectivity. Additionally, secondary alkyl bromides, such as 2-bromopropanes, cyclopentyl bromide, cyclohexyl bromides, and 2-bromobicyclo [2.2.1]heptane, were likewise amenable (**40**–**43**).

## 3. Discussion

**Scheme 4:**
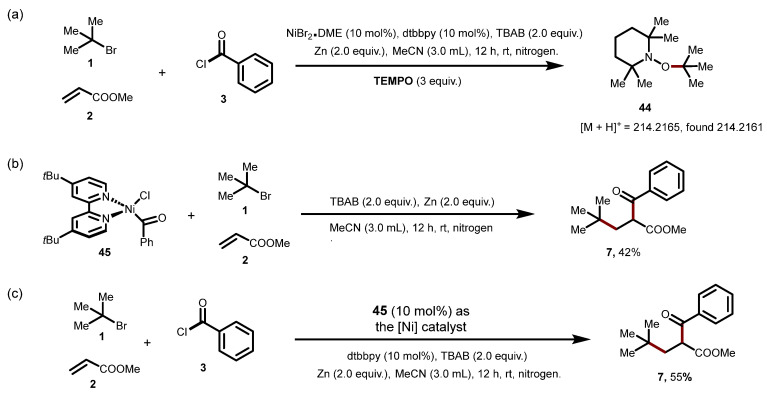
Mechanistic studies.

To gain mechanistic insights into this addition reaction, we conducted a series of experimental studies. Adding 3.0 equivalents of TEMPO significantly inhibited these transformations and prevented the detection of the corresponding products. Consequently, we detected the TEMPO-trapped species **44** using HR-MS (Scheme 4a). Moreover, we did not observe the corresponding product when using the pre-isolated Ni(II) complex **45** as a substrate in reactions with *tert*-butyl bromides **1a** and methylacrylate **2a** (Scheme 4b). This finding suggests that oxidative addition occurs subsequent to the alkyl capture by the nickel (II) complex **45**. However, when the reaction was conducted using 10 mol% of **45** as a catalyst, we achieved a 55% yield of the additional product **7** (Scheme 4c).

**Scheme 5:**
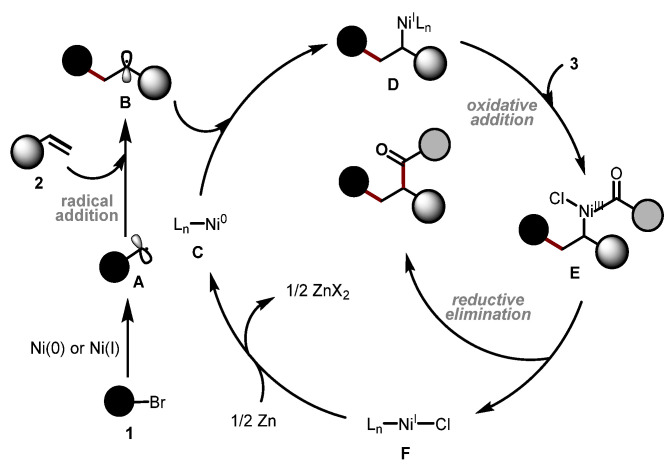
Possible reaction mechanism.

Based on our investigation and previous findings [36,37,38,39,40,41,42,43,44,45,46,47,48], we propose a plausible mechanism for this alkene addition (Scheme 5). Initially, the alkyl bromide is converted to an alkyl radical by a low-valent nickel species. Subsequently, intermediate **A** reacts with alkene **2**, forming secondary alkyl radical B, which is captured by nickel(0) species **C** to form the alkyl-Ni(I) intermediate **D**. Intermediate **D** undergoes a concerted oxidative addition with acyl chloride **3**, producing Ni(III) species **E**, which then undergoes reductive elimination to yield the target carbonyl product and reform the Ni(I) species **F**. The latter is then reduced by zinc powder to regenerate the Ni(0) species **C**.

## 4. Materials and Methods


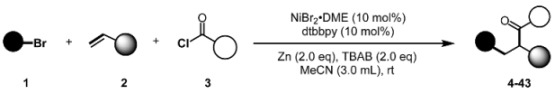


The mixture of **1** (1.0 mmol), **2** (0.2 mmol), **3** (0.6 mmol), NiBr_2_•DME (10 mol%), dtbbpy (10 mol%), TBAB (2.0 equiv.), and Zn (2.0 equiv.) was protected with N_2_ in anhydrous MeCN (3 mL). Then, the mixture was stirred at room temperature for 12 h. Removal of the solvent under reduced pressure afforded a residue that was purified by chromatography on silica gel to afford the products **4**–**43**. The general experimental details and NMR spectra of the compounds can be found in the Supplementary Materials.

## 5. Conclusions

In conclusion, our study reports on a nickel-catalyzed three-component alkenes carboacylation via the activation of alkyl bromides. This electrocatalytic platform facilitates the efficient synthesis of valuable ketones from cost-effective commercial materials, achieving excellent regioselectivity. Several mechanistic studies elucidated the key reaction pathways involved in the cascade process. We contend that this strategy offers a versatile platform for synthesizing value-added ketones in an environmentally friendly and sustainable manner.

## 6. Characterization Data of Products

*Methyl-4,4-dimethyl-2-(4-methylbenzoyl)pentanoate* (**4**). This compound was prepared according to the general procedures, 40.9 mg, 78% yield as a colorless oil. ^1^H NMR (400 MHz, Chloroform-*d*) δ 7.91 (d, *J* = 8.0 Hz, 2H), 7.28 (d, *J* = 8.6 Hz, 2H), 4.40 (t, *J* = 6.0 Hz, 1H), 3.67 (s, 3H), 2.42 (s, 3H), 2.03 (d, *J* = 6.0 Hz, 2H), 0.90 (s, 9H). ^13^C NMR (101 MHz, Chloroform-*d*) δ 195.1, 171.1, 144.4, 133.5, 129.5, 128.9, 52.5, 50.5, 42.0, 30.9, 29.4, 21.7. HR-MS (ESI) C_16_H_23_O_3_ [M + H]^+^: 263.1642, found: 263.1644.
*Methyl-4,4-dimethyl-2-(2-methylbenzoyl)pentanoate* (**5**). This compound was prepared according to the general procedures, 33.5 mg, 64% yield as a colorless oil. ^1^H NMR (400 MHz, Chloroform-*d*) δ 7.70 (d, *J* = 7.7 Hz, 1H), 7.39 (t, *J* = 7.4 Hz, 1H), 7.29–7.25 (m, 2H), 4.28 (dd, *J* = 7.0, 5.0 Hz, 1H), 3.68 (s, 3H), 2.47 (s, 3H), 2.04–1.91 (m, 2H), 0.89 (s, 9H). ^13^C NMR (101 MHz, Chloroform-*d*) δ 198.9, 171.2, 139.1, 136.8, 132.1, 131.6, 128.3, 125.7, 53.4, 52.4, 41.7, 30.8, 29.2, 21.1. HR-MS (ESI) C_16_H_23_O_3_ [M + H]^+^: 263.1642, found: 263.1642.
*Methyl-4,4-dimethyl-2-(3-methylbenzoyl)pentanoate* (**6**). This compound was prepared according to the general procedures, 43 mg, 82% yield as a colorless oil. ^1^H NMR (400 MHz, Chloroform-*d*) δ 7.80 (d, *J* = 7.2 Hz, 2H), 7.41–7.35 (m, 2H), 4.41 (t, *J* = 6.0 Hz, 1H), 3.68 (s, 3H), 2.42 (s, 3H), 2.03 (d, *J* = 6.0 Hz, 2H), 0.91 (s, 9H). ^13^C NMR (101 MHz, Chloroform-*d*) δ 195.7, 171.1, 138.7, 136.0, 134.3, 129.2, 128.6, 125.9, 52.6, 50.7, 42.0, 30.9, 29.4, 21.4. HR-MS (ESI) C_16_H_23_O_3_ [M + H]^+^: 263.1642, found: 263.1644.
*Methyl-2-benzoyl-4,4-dimethylpentanoate* (**7**). This compound was prepared according to the general procedures, 37.2 mg, 75% yield as a colorless oil. ^1^H NMR (400 MHz, Chloroform-*d*) δ 8.04–7.97 (m, 2H), 7.59 (t, *J* = 7.4 Hz, 1H), 7.52–7.45 (m, 2H), 4.42 (t, *J* = 6.0 Hz, 1H), 3.68 (s, 3H), 2.04 (d, *J* = 6.0 Hz, 2H), 0.91 (s, 9H). ^13^C NMR (101 MHz, Chloroform-*d*) δ 195.5, 171.0, 136.0, 133.5, 128.8, 128.7, 52.6, 50.7, 42.0, 30.9, 29.4. HR-MS (ESI) C_15_H_21_O_3_ [M + H]^+^: 249.1485, found: 249.1486.
*Methyl-2-(2-naphthoyl)-4,4-dimethylpentanoate* (**8**). This compound was prepared according to the general procedures, 43 mg, 72% yield as a colorless oil. ^1^H NMR (400 MHz, Chloroform-*d*) δ 8.56 (s, 1H), 8.06 (dd, *J* = 8.6, 1.7 Hz, 1H), 8.00 (d, *J* = 8.1 Hz, 1H), 7.90 (dd, *J* = 11.3, 8.4 Hz, 2H), 7.64–7.56 (m, 2H), 4.58 (t, *J* = 6.0 Hz, 1H), 3.69 (s, 3H), 2.10 (d, *J* = 5.9 Hz, 2H), 0.94 (s, 9H). ^13^C NMR (101 MHz, Chloroform-*d*) δ 195.4, 171.1, 135.7, 133.4, 132.5, 130.6, 129.8, 128.8, 128.7, 127.8, 126.9, 124.3, 52.6, 50.8, 42.1, 31.0, 29.4. HR-MS (ESI) C_19_H_23_O_3_ [M + H]^+^: 299.1642, found: 299.1645.
*Methyl-2-(4-(tert-butyl)benzoyl)-4,4-dimethylpentanoate* (**9**). This compound was prepared according to the general procedures, 48.7 mg, 80% yield as a colorless oil. ^1^H NMR (400 MHz, Chloroform-*d*) δ 7.96 (d, *J* = 8.5 Hz, 2H), 7.50 (d, *J* = 8.6 Hz, 2H), 4.42 (t, *J* = 6.0 Hz, 1H), 3.68 (s, 3H), 2.04 (d, *J* = 6.0 Hz, 2H), 1.35 (s, 9H), 0.91 (s, 9H). ^13^C NMR (101 MHz, Chloroform-*d*) δ 195.0, 171.1, 157.3, 133.4, 128.7, 125.8, 52.5, 50.5, 42.0, 35.2, 31.1, 30.9, 29.4. HR-MS (ESI) C_19_H_29_O_3_ [M + H]^+^: 305.2111, found: 305.2112.
Methyl-2-(4-methoxybenzoyl)-4,4-dimethylpentanoate (**10**). This compound was prepared according to the general procedures, 39.5 mg, 71% yield as a colorless oil. ^1^H NMR (400 MHz, Chloroform-*d*) δ 8.01 (d, *J* = 8.8 Hz, 2H), 6.96 (d, *J* = 8.8 Hz, 2H), 4.38 (t, *J* = 6.0 Hz, 1H), 3.88 (s, 3H), 3.67 (s, 3H), 2.06–1.99 (m, 2H), 0.91 (s, 9H). ^13^C NMR (101 MHz, Chloroform-*d*) δ 193.9, 171.2, 163.8, 131.1, 129.0, 114.0, 55.5, 52.5, 50.4, 42.0, 30.9, 29.4. HR-MS (ESI) C_16_H_23_O_4_ [M + H]^+^: 279.1591, found: 279.1590.
*Methyl-2-(3-methoxybenzoyl)-4,4-dimethylpentanoate* (**11**). This compound was prepared according to the general procedures, 33.4 mg, 60% yield as a colorless oil. ^1^H NMR (400 MHz, Chloroform-*d*) δ 7.60 (d, *J* = 7.7 Hz, 1H), 7.54–7.51 (m, 1H), 7.39 (t, *J* = 8.0 Hz, 1H), 7.13 (dd, *J* = 8.2, 2.1 Hz, 1H), 4.40 (t, *J* = 6.0 Hz, 1H), 3.86 (s, 3H), 3.68 (s, 3H), 2.03 (d, *J* = 6.0 Hz, 2H), 0.91 (s, 9H). ^13^C NMR (101 MHz, Chloroform-*d*) δ 195.3, 171.0, 160.0, 137.4, 129.7, 121.2, 120.1, 113.0, 55.5, 52.6, 50.8, 42.0, 30.9, 29.4. HR-MS (ESI) C_16_H_23_O_4_ [M + H]^+^: 279.1591, found: 279.1591.
*Methyl-4,4-dimethyl-2-(4-(trifluoromethyl)benzoyl)pentanoate* (**12**). This compound was prepared according to the general procedures, 39.2 mg, 62% yield as a colorless oil. ^1^H NMR (400 MHz, Chloroform-*d*) δ 8.11 (d, *J* = 8.1 Hz, 2H), 7.76 (d, *J* = 8.2 Hz, 2H), 4.39 (t, *J* = 6.0 Hz, 1H), 3.69 (s, 3H), 2.05 (d, *J* = 6.0 Hz, 2H), 0.91 (s, 9H). ^13^C NMR (101 MHz, Chloroform-*d*) δ 194.6, 170.5, 138.8, 135.1, 129.1, 127.5, 125.9 (q, *J* = 4.0 HZ), 52.8, 51.0, 41.8, 30.9, 29.3. ^19^F NMR (376 MHz, Chloroform-*d*) δ -63.20. HR-MS (ESI) C_16_H_20_F_3_O_3_ [M + H]^+^: 317.1359, found: 317.1363.
*Methyl-2-(4-fluorobenzoyl)-4,4-dimethylpentanoate* (**13**). This compound was prepared according to the general procedures, 41.0 mg, 77% yield as a colorless oil. ^1^H NMR (400 MHz, Chloroform-*d*) δ 8.07-8.03 (m, 2H), 7.20–7.11 (m, 2H), 4.38 (t, *J* = 6.0 Hz, 1H), 3.68 (s, 3H), 2.04 (d, *J* = 6.0 Hz, 2H), 0.91 (s, 9H). ^13^C NMR (101 MHz, Chloroform-*d*) δ 193.8, 170.8, 165.9 (d, *J* = 255.7 Hz), 132.4 (d, *J* = 2.9 Hz), 131.4 (d, *J* = 9.4 Hz), 128.7 (d, *J* = 4.9 Hz), 115.9 (d, *J* = 21.9 Hz), 52.6, 50.7, 41.9, 30.8, 29.3. ^19^F NMR (376 MHz, Chloroform-*d*) δ -104.4. HR-MS (ESI) C_15_H_20_FO_3_ [M + H]^+^: 267.1391, found: 267.1392.
*Methyl-2-(4-chlorobenzoyl)-4,4-dimethylpentanoate* (**14**). This compound was prepared according to the general procedures, 40.7 mg, 72% yield as a colorless oil. ^1^H NMR (400 MHz, Chloroform-*d*) δ 7.98–7.93 (m, 2H), 7.49–7.44 (m, 2H), 4.36 (t, *J* = 6.0 Hz, 1H), 3.68 (s, 3H), 2.03 (d, *J* = 6.0 Hz, 2H), 0.90 (s, 9H). ^13^C NMR (101 MHz, Chloroform-*d*) δ 194.3, 170.7, 140.0, 134.3, 130.1, 129.1, 52.7, 50.7, 41.9, 30.8, 29.3. HR-MS (ESI) C_15_H_20_ClO_3_ [M + H]^+^: 283.1095, found: 283.1099.
*Methyl-2-(benzofuran-5-carbonyl)-4,4-dimethylpentanoate* (**15**). This compound was prepared according to the general procedures, 42.1 mg, 73% yield as a colorless oil. ^1^H NMR (400 MHz, Chloroform-*d*) δ 8.33 (d, *J* = 1.5 Hz, 1H), 8.02 (dd, *J* = 8.7, 1.7 Hz, 1H), 7.71 (d, *J* = 2.1 Hz, 1H), 7.57 (d, *J* = 8.7 Hz, 1H), 6.90–6.88 (m, 1H), 4.51 (t, *J* = 6.0 Hz, 1H), 3.69 (s, 3H), 2.08 (dd, *J* = 6.0, 2.6 Hz, 2H), 0.92 (s, 9H). ^13^C NMR (101 MHz, Chloroform-*d*) δ 194.9, 171.2, 157.7, 146.6, 131.6, 127.7, 125.5, 123.1, 111.8, 107.4, 52.6, 50.8, 42.2, 30.9, 29.4. HR-MS (ESI) C_17_H_21_O_4_ [M + H]^+^: 289.1434, found: 289.1440.
*Methyl-2-(furan-2-carbonyl)-4,4-dimethylpentanoate* (**16**). This compound was prepared according to the general procedures, 36.2 mg, 76% yield as a colorless oil. ^1^H NMR (400 MHz, Chloroform-*d*) δ 7.64–7.62 (m, 1H), 7.31 (d, *J* = 3.6 Hz, 1H), 6.57 (dd, *J* = 3.6, 1.6 Hz, 1H), 4.22 (s, 1H), 3.69 (s, 3H), 2.02 (d, *J* = 6.0 Hz, 2H), 0.91 (s, 9H). ^13^C NMR (101 MHz, Chloroform-*d*) δ 184.3, 170.7, 151.8, 147.0, 118.5, 112.6, 52.6, 50.9, 41.5, 30.8, 29.3. HR-MS (ESI) C_12_H_17_O_4_ [M + H]^+^: 225.1121, found: 225.1127.
*Ethyl-4,4-dimethyl-2-(4-methylbenzoyl)pentanoate* (**17**). This compound was prepared according to the general procedures, 42.6 mg, 77% yield as a colorless oil. ^1^H NMR (400 MHz, Chloroform-*d*) δ 7.86–7.81 (m, 2H), 7.20 (d, *J* = 7.8 Hz, 2H), 4.29 (t, *J* = 6.0 Hz, 1H), 4.06 (q, *J* = 7.1 Hz, 2H), 2.34 (s, 3H), 1.94 (d, *J* = 5.9 Hz, 2H), 1.10 (t, *J* = 7.1 Hz, 3H), 0.84 (s, 9H). ^13^C NMR (101 MHz, Chloroform-*d*) δ 194.1, 169.6, 143.2, 132.6, 128.4, 127.9, 60.3, 49.8, 40.9, 29.9, 28.4, 20.6, 12.9. HR-MS (ESI) C_17_H_25_O_3_ [M + H]^+^: 277.1798, found: 277.1800.
*Tert-butyl-4,4-dimethyl-2-(4-methylbenzoyl)pentanoate* (**18**). This compound was prepared according to the general procedures, 50.5 mg, 83% yield as a colorless oil. ^1^H NMR (400 MHz, Chloroform-*d*) δ 7.90 (d, *J* = 8.2 Hz, 2H), 7.30–7.23 (m, 2H), 4.23 (t, *J* = 5.9 Hz, 1H), 2.42 (s, 3H), 1.98 (dd, *J* = 5.9, 1.2 Hz, 2H), 1.35 (s, 9H), 0.91 (s, 9H). ^13^C NMR (101 MHz, Chloroform-*d*) δ 195.6, 169.8, 144.0, 133.9, 129.3, 128.9, 81.5, 52.0, 41.6, 30.8, 29.5, 27.7, 21.6. HR-MS (ESI) C_19_H_29_O_3_ [M + H]^+^: 305.2111, found: 305.2115.
*Isobutyl-4,4-dimethyl-2-(4-methylbenzoyl)pentanoate* (**19**). This compound was prepared according to the general procedures, 48.1 mg, 79% yield as a colorless oil. ^1^H NMR (400 MHz, Chloroform-*d*) δ 7.92 (d, *J* = 8.1 Hz, 2H), 7.27 (d, *J* = 6.9 Hz, 2H), 4.39 (t, *J* = 6.0 Hz, 1H), 3.84 (d, *J* = 6.6 Hz, 2H), 2.42 (s, 3H), 2.10–1.99 (m, 2H), 1.85 (dt, *J* = 13.4, 6.7 Hz, 1H), 0.91 (s, 9H), 0.82 (d, *J* = 6.7 Hz, 6H). ^13^C NMR (101 MHz, Chloroform-*d*) δ 195.1, 170.7, 144.2, 133.7, 129.4, 128.9, 71.5, 50.7, 41.8, 30.8, 29.4, 27.6, 21.6, 18.9. HR-MS (ESI) C_19_H_29_O_3_ [M + H]^+^: 305.2111, found: 305.2114.
*Cyclohexyl-4,4-dimethyl-2-(4-methylbenzoyl)pentanoate* (**20**). This compound was prepared according to the general procedures, 48.9 mg, 74% yield as a colorless oil. ^1^H NMR (400 MHz, Chloroform-*d*) δ 7.92 (d, *J* = 8.2 Hz, 2H), 7.27 (d, *J* = 4.9 Hz, 2H), 4.76 (td, *J* = 8.5, 3.9 Hz, 1H), 4.32 (t, *J* = 5.9 Hz, 1H), 2.42 (s, 3H), 2.01 (d, *J* = 6.0 Hz, 2H), 1.77–1.57 (m, 5H), 1.39–1.22 (m, 5H), 0.91 (s, 9H). ^13^C NMR (101 MHz, Chloroform-*d*) δ 195.2, 170.1, 144.1, 133.7, 129.3, 128.9, 73.5, 51.2, 41.7, 31.2, 31.1, 30.9, 29.4, 25.3, 23.4, 23.4, 21.7. HR-MS (ESI) C_21_H_31_O_3_ [M + H]^+^: 331.2268, found: 331.2270.
*Phenyl-4,4-dimethyl-2-(4-methylbenzoyl)pentanoate* (**21**). This compound was prepared according to the general procedures, 40.5 mg, 63% yield as a colorless oil. ^1^H NMR (400 MHz, Chloroform-*d*) δ 7.98 (d, *J* = 8.1 Hz, 2H), 7.34-7.29 (m, 4H), 7.18 (t, *J* = 7.4 Hz, 1H), 6.99 (d, *J* = 8.1 Hz, 2H), 4.59 (dd, *J* = 7.2, 4.7 Hz, 1H), 2.42 (s, 3H), 2.19 (dd, *J* = 14.4, 7.3 Hz, 1H), 2.05 (dd, *J* = 14.4, 4.6 Hz, 1H), 1.00 (s, 9H). ^13^C NMR (101 MHz, Chloroform-*d*) δ 194.9, 169.4, 150.7, 144.6, 133.3, 129.6, 129.4, 129.0, 126.0, 121.3, 51.0, 41.9, 31.1, 29.5, 21.7. HR-MS (ESI) C_21_H_25_O_3_ [M + H]^+^: 325.1798, found: 325.1800.
*Benzyl-4,4-dimethyl-2-(4-methylbenzoyl)pentanoate* (**22**). This compound was prepared according to the general procedures, 48.7 mg, 72% yield as a colorless oil. ^1^H NMR (400 MHz, Chloroform-*d*) δ 7.88 (d, *J* = 8.2 Hz, 2H), 7.29–7.19 (m, 7H), 5.14–5.07 (m, 2H), 4.41 (t, *J* = 6.0 Hz, 1H), 2.41 (s, 3H), 2.04 (d, *J* = 6.0 Hz, 2H), 0.89 (s, 9H). ^13^C NMR (101 MHz, Chloroform-*d*) δ 195.0, 170.5, 144.3, 135.5, 133.5, 129.4, 128.9, 128.5, 128.2, 128.1, 67.1, 50.8, 41.9, 30.9, 29.4, 21.7. HR-MS (ESI) C_22_H_27_O_3_ [M + H]^+^: 339.1955, found: 339.1961.
*2-Methoxyethyl-4,4-dimethyl-2-(4-methylbenzoyl)pentanoate* (**23**). This compound was prepared according to the general procedures, 35.5 mg, 58% yield as a colorless oil. ^1^H NMR (400 MHz, Chloroform-*d*) δ 7.91 (d, *J* = 8.0 Hz, 2H), 7.29–7.26 (m, 2H), 4.43-4.40 (m, 1H), 4.30–4.19 (m, 2H), 3.55–3.47 (m, 2H), 3.28 (s, 3H), 2.42 (s, 3H), 2.07–1.96 (m, 2H), 0.92 (s, 9H). ^13^C NMR (101 MHz, Chloroform-*d*) δ 195.0, 170.6, 144.3, 133.5, 129.4, 128.9, 70.2, 64.3, 58.8, 50.7, 41.9, 30.9, 29.4, 21.7. HR-MS (ESI) C_18_H_27_O_4_ [M + H]^+^: 307.1904, found: 307.1905.
*2,2,2-Trifluoroethyl-4,4-dimethyl-2-(4-methylbenzoyl)pentanoate* (**24**). This compound was prepared according to the general procedures, 35.0 mg, 53% yield as a colorless oil. ^1^H NMR (400 MHz, Chloroform-*d*) δ 7.93–7.88 (m, 2H), 7.28 (d, *J* = 7.7 Hz, 2H), 4.53 (dd, *J* = 7.0, 4.5 Hz, 1H), 2.51–2.44 (m, 2H), 2.42 (s, 3H), 2.20–2.15 (m, 1H), 1.84 (dd, *J* = 14.1, 4.5 Hz, 1H), 0.87 (s, 9H). ^13^C NMR (101 MHz, Chloroform-*d*) δ 206.8, 196.1, 144.5, 134.0, 129.5, 128.9, 59.6, 41.8, 33.8, 31.1, 29.5, 21.6, 7.8. HR-MS (ESI) C_17_H_22_F_3_O_3_ [M + H]^+^: 331.1516, found: 331.1520.
*2-Chloroethyl-4,4-dimethyl-2-(4-methylbenzoyl)pentanoate* (**25**). This compound was prepared according to the general procedures, 26.1 mg, 42% yield as a colorless oil. ^1^H NMR (400 MHz, Chloroform-*d*) δ 7.91 (d, *J* = 8.2 Hz, 2H), 7.28 (d, *J* = 8.3 Hz, 2H), 4.43 (dd, *J* = 6.7, 5.3 Hz, 1H), 4.33 (4.37-4.28, m, 2H), 3.65–3.56 (m, 2H), 2.42 (s, 3H), 2.08–1.98 (m, 2H), 0.92 (s, 9H). ^13^C NMR (101 MHz, Chloroform-*d*) δ 194.8, 170.3, 144.5, 133.3, 129.5, 128.9, 64.7, 50.5, 41.9, 41.2, 30.9, 29.4, 21.7. HR-MS (ESI) C_17_H_24_ClO_3_ [M + H]^+^: 311.1408, found: 311.1410.
*Methyl-4-(4,4-dimethyl-1-oxo-1-(p-tolyl)pentan-2-yl)benzoate* (**26**). This compound was prepared according to the general procedures, 27.1 mg, 40% yield as a colorless oil. ^1^H NMR (400 MHz, Chloroform-*d*) δ 7.95–7.88 (m, 4H), 7.39 (d, *J* = 8.3 Hz, 2H), 7.22 (d, *J* = 8.0 Hz, 2H), 4.78-4.75 (m, 1H), 3.91-3.87 (m, 4H), 2.63-2.58 (m, 1H), 2.37 (s, 3H), 0.88 (s, 9H). ^13^C NMR (101 MHz, Chloroform-*d*) δ 199.0, 166.9, 146.5, 143.9, 134.2, 130.2, 129.4, 129.3, 128.7, 128.2, 52.1, 49.5, 47.4, 31.3, 29.8, 21.6. HR-MS (ESI) C_22_H_27_O_3_ [M + H]^+^: 339.1955, found: 339.1955.
*Methyl-6-(benzyloxy)-4,4-dimethyl-2-(4-methylbenzoyl)hexanoate* (**27**). This compound was prepared according to the general procedures, 47.4 mg, 62% yield as a colorless oil. ^1^H NMR (400 MHz, Chloroform-*d*) δ 7.99–7.85 (m, 2H), 7.39–7.19 (m, 7H), 4.45 (d, *J* = 3.3 Hz, 2H), 3.73–3.48 (m, 5H), 2.41 (d, *J* = 2.9 Hz, 3H), 2.12–2.00 (m, 2H), 1.72 (s, 1H), 1.61-1.58 (m, 2H), 0.89 (d, *J* = 4.6 Hz, 6H). ^13^C NMR (101 MHz, Chloroform-*d*) δ 194.9, 171.0, 144.4, 138.5, 133.5, 129.5, 128.9, 128.3, 127.6, 127.5, 73.0, 67.1, 52.6, 50.0, 41.1, 40.5, 32.7, 27.4, 27.3, 21.7. HR-MS (ESI) C_24_H_31_O_4_ [M + H]^+^: 383.2217, found: 383.2222.
*6-methoxy-3,3-dimethyl-5-(4-methylbenzoyl)-6-oxohexyl benzoate* (**28**). This compound was prepared according to the general procedures, 62.6 mg, 79% yield as a colorless oil. ^1^H NMR (400 MHz, Chloroform-*d*) δ 8.03–7.98 (m, 2H), 7.95–7.90 (m, 2H), 7.57–7.51 (m, 1H), 7.43-7.40 (m, 2H), 7.29–7.25 (m, 2H), 4.46 (t, *J* = 6.0 Hz, 1H), 4.41-4.38 (m, 2H), 3.66 (s, 3H), 2.41 (s, 3H), 2.21–2.09 (m, 2H), 1.73 (t, *J* = 7.0 Hz, 2H), 0.97 (s, 6H). ^13^C NMR (101 MHz, Chloroform-*d*) δ 194.7, 170.9, 166.6, 144.5, 133.4, 132.9, 130.3, 129.5, 128.9, 128.3, 62.0, 52.6, 49.9, 40.5, 40.2, 32.8, 27.1, 27.0, 21.7. HR-MS (ESI) C_24_H_29_O_5_ [M + H]^+^: 397.2010, found: 397.2011.
*6-methoxy-3,3-dimethyl-5-(4-methylbenzoyl)-6-oxohexyl 2-naphthoate* (**29**). This compound was prepared according to the general procedures, 63.4 mg, 71% yield as a colorless oil. ^1^H NMR (400 MHz, Chloroform-*d*) δ 8.49 (s, 1H), 7.94 (dd, *J* = 8.6, 1.7 Hz, 1H), 7.85 (dd, *J* = 10.4, 8.5 Hz, 3H), 7.78 (dd, *J* = 8.3, 3.6 Hz, 2H), 7.52–7.44 (m, 2H), 7.17 (d, *J* = 7.8 Hz, 2H), 4.41–4.33 (m, 3H), 3.58 (s, 3H), 2.31 (s, 3H), 2.17–2.05 (m, 2H), 1.71 (t, *J* = 7.2 Hz, 2H), 0.92 (s, 6H). ^13^C NMR (101 MHz, Chloroform-*d*) δ 194.7, 171.0, 166.7, 144.5, 135.5, 133.5, 132.5, 131.0, 129.5, 129.4, 128.9, 128.2, 128.1, 127.7, 127.6, 126.6, 125.2, 62.1, 52.6, 50.0, 40.5, 40.3, 32.9, 27.1, 27.0, 21.7. HR-MS (ESI) C_28_H_31_O_5_ [M + H]^+^: 447.2166, found: 447.2175.
*6-methoxy-3,3-dimethyl-5-(4-methylbenzoyl)-6-oxohexyl 4-methylbenzoate* (**30**). This compound was prepared according to the general procedures, 67.3 mg, 82% yield as a colorless oil. ^1^H NMR (400 MHz, Chloroform-*d*) δ 7.93-7.88 (m, 4H), 7.27 (d, *J* = 7.1 Hz, 2H), 7.21 (d, *J* = 7.9 Hz, 2H), 4.45 (t, *J* = 6.0 Hz, 1H), 4.38-4.32 (m, 2H), 3.66 (s, 3H), 2.40 (d, *J* = 5.7 Hz, 6H), 2.20–2.08 (m, 2H), 1.72 (t, *J* = 7.2 Hz, 2H), 0.97 (s, 6H). ^13^C NMR (101 MHz, Chloroform-*d*) δ 194.7, 170.9, 166.6, 144.5, 143.5, 133.5, 129.5, 129.5, 129.0, 128.9, 127.6, 61.7, 52.6, 50.0, 40.6, 40.2, 32.8, 27.1, 27.0, 21.7, 21.6. HR-MS (ESI) C_25_H_31_O_5_ [M + H]^+^: 411.2166, found: 411.2171.
*6-methoxy-3,3-dimethyl-5-(4-methylbenzoyl)-6-oxohexyl 3-methylbenzoate* (**31**). This compound was prepared according to the general procedures, 63.2 mg, 77% yield as a colorless oil. ^1^H NMR (400 MHz, Chloroform-*d*) δ 7.95–7.90 (m, 2H), 7.84–7.77 (m, 2H), 7.37–7.26 (m, 4H), 4.47–4.32 (m, 3H), 3.66 (s, 3H), 2.40 (d, *J* = 7.6 Hz, 6H), 2.21–2.11 (m, 2H), 1.73 (t, *J* = 7.3 Hz, 2H), 0.97 (s, 6H). ^13^C NMR (101 MHz, Chloroform-*d*) δ 194.7, 170.9, 166.8, 144.5, 138.1, 133.6, 133.4, 130.2, 130.1, 129.5, 128.9, 128.2, 126.7, 61.9, 52.6, 50.0, 40.5, 40.2, 32.8, 27.1, 27.0, 21.7, 21.3. HR-MS (ESI) C_25_H_31_O_5_ [M + H]^+^: 411.2166, found: 411.2171.
*6-methoxy-3,3-dimethyl-5-(4-methylbenzoyl)-6-oxohexyl 2-methylbenzoate* (32). This compound was prepared according to the general procedures, 62.4 mg, 76% yield as a colorless oil. ^1^H NMR (400 MHz, Chloroform-*d*) δ 7.84 (d, *J* = 8.3 Hz, 2H), 7.78 (d, *J* = 8.0 Hz, 1H), 7.32–7.28 (m, 1H), 7.21–7.18 (m, 2H), 7.15 (d, *J* = 7.5 Hz, 2H), 4.38 (t, *J* = 6.0 Hz, 1H), 4.31–4.21 (m, 2H), 3.58 (s, 3H), 2.50 (s, 3H), 2.34 (s, 3H), 2.12–2.01 (m, 2H), 1.67–1.62 (m, 2H), 0.89 (s, 6H). ^13^C NMR (101 MHz, Chloroform-*d*) δ 194.7, 170.9, 167.6, 144.5, 140.1, 133.5, 131.9, 131.7, 130.5, 129.5, 128.9, 125.7, 61.7, 52.6, 49.9, 40.5, 40.2, 32.8, 27.1, 27.0, 21.7, 21.7. HR-MS (ESI) C_25_H_31_O_5_ [M + H]^+^: 411.2166, found: 411.2171.
*6-methoxy-3,3-dimethyl-5-(4-methylbenzoyl)-6-oxohexyl 4-methoxybenzoate* (**33**). This compound was prepared according to the general procedures, 75.0 mg, 64% yield as a colorless oil. ^1^H NMR (400 MHz, Chloroform-*d*) δ 7.99–7.90 (m, 4H), 7.27 (d, *J* = 6.8 Hz, 2H), 6.89 (d, *J* = 8.9 Hz, 2H), 4.45 (t, *J* = 6.0 Hz, 1H), 4.39-4.29 (m, 2H), 3.85 (s, 3H), 3.66 (s, 3H), 2.41 (s, 3H), 2.20–2.08 (m, 2H), 1.71 (t, *J* = 7.2 Hz, 2H), 0.96 (s, 6H). ^13^C NMR (101 MHz, Chloroform-*d*) δ 194.7, 170.9, 166.3, 163.3, 144.5, 133.5, 131.5, 129.5, 128.9, 122.8, 113.6, 61.6, 55.4, 52.6, 50.0, 40.6, 40.2, 32.8, 27.1, 27.0, 21.7. HR-MS (ESI) C_25_H_31_O_6_ [M + H]^+^: 427.2115, found: 427.2120.
*6-methoxy-3,3-dimethyl-5-(4-methylbenzoyl)-6-oxohexyl 4-(trifluoromethyl)benzoate* (**34**). This compound was prepared according to the general procedures, 70.6 mg, 76% yield as a colorless oil. ^1^H NMR (400 MHz, Chloroform-*d*) δ 8.11 (d, *J* = 8.1 Hz, 2H), 7.92 (d, *J* = 8.0 Hz, 2H), 7.68 (d, *J* = 8.2 Hz, 2H), 7.29–7.26 (m, 2H), 4.47–4.37 (m, 3H), 3.67 (s, 3H), 2.41 (s, 3H), 2.22–2.10 (m, 2H), 1.74 (t, *J* = 7.2 Hz, 2H), 0.98 (d, *J* = 2.1 Hz, 6H). ^13^C NMR (101 MHz, Chloroform-*d*) δ 194.5, 170.9, 165.3, 144.6, 134.5, 134.2, 133.5, 133.4, 129.9, 129.5, 128.9, 128.1, 125.4, 125.4, 125.3, 62.5, 52.7, 50.0, 40.4, 40.2, 32.8, 27.1, 27.0, 21.6. ^19^F NMR (376 MHz, Chloroform-*d*) δ −63.11. HR-MS (ESI) C_25_H_28_F_3_O_5_ [M + H]^+^: 465.1883, found: 465.1889.
*6-methoxy-3,3-dimethyl-5-(4-methylbenzoyl)-6-oxohexyl 4-cyanobenzoate* (**35**). This compound was prepared according to the general procedures, 53.1 mg, 63% yield as a colorless oil. ^1^H NMR (400 MHz, Chloroform-*d*) δ 8.02 (d, *J* = 8.6 Hz, 2H), 7.84 (d, *J* = 8.3 Hz, 2H), 7.64 (d, *J* = 8.6 Hz, 2H), 7.20 (d, *J* = 8.0 Hz, 2H), 4.39–4.30 (m, 3H), 3.59 (s, 3H), 2.34 (s, 3H), 2.15–2.00 (m, 2H), 1.69–1.64 (m, 2H), 0.90 (d, *J* = 4.3 Hz, 6H). ^13^C NMR (101 MHz, Chloroform-*d*) δ 194.5, 170.9, 164.9, 144.6, 134.1, 133.4, 132.2, 130.0, 129.5, 128.9, 118.0, 116.3, 62.8, 52.7, 49.9, 40.3, 40.2, 32.8, 27.1, 27.0, 21.7. HR-MS (ESI) C_25_H_28_NO_5_ [M + H]^+^: 422.1962, found: 422.1966.
*6-methoxy-3,3-dimethyl-5-(4-methylbenzoyl)-6-oxohexyl 4-fluorobenzoate* (**36**). This compound was prepared according to the general procedures, 58.9 mg, 71% yield as a colorless oil. ^1^H NMR (400 MHz, Chloroform-*d*) δ 8.04–7.99 (m, 2H), 7.95–7.89 (m, 2H), 7.28 (d, *J* = 7.5 Hz, 2H), 7.09 (t, *J* = 8.6 Hz, 2H), 4.47–4.32 (m, 3H), 3.67 (s, 3H), 2.42 (s, 3H), 2.21-2.01 (m, 2H), 1.72 (t, *J* = 7.2 Hz, 2H), 0.97 (s, 6H). ^13^C NMR (101 MHz, Chloroform-*d*) δ 194.6, 170.9, 167.0, 165.6, 164.4, 144.5, 133.4, 132.1, 132.0, 129.5, 128.9, 126.6, 126.5, 115.6, 115.3, 62.1, 52.6, 50.0, 40.5, 40.2, 32.8, 27.1, 27.0, 21.7. HR-MS (ESI) C_24_H_28_FO_5_ [M + H]^+^: 415.1915, found:415.1918.
*6-methoxy-3,3-dimethyl-5-(4-methylbenzoyl)-6-oxohexyl 4-chlorobenzoate* (**37**). This compound was prepared according to the general procedures, 56.0 mg, 65% yield as a colorless oil. ^1^H NMR (400 MHz, Chloroform-*d*) δ 7.94–7.91 (m, 4H), 7.38 (d, *J* = 8.6 Hz, 2H), 7.29–7.26 (m, 2H), 4.47–4.34 (m, 3H), 3.66 (s, 3H), 2.41 (s, 3H), 2.21–2.09 (m, 2H), 1.73 (d, *J* = 7.0 Hz, 2H), 0.97 (s, 6H). ^13^C NMR (101 MHz, Chloroform-*d*) δ 194.6, 170.9, 165.7, 144.5, 139.3, 133.4, 130.9, 129.5, 128.9, 128.8, 128.7, 62.2, 52.6, 50.0, 40.4, 40.2, 32.8, 27.1, 27.0, 21.7. HR-MS (ESI) C_24_H_28_ClO_5_ [M + H]^+^: 431.1620, found: 431.1627.
*6-methoxy-3,3-dimethyl-5-(4-methylbenzoyl)-6-oxohexyl thiophene-2-carboxylate* (**38**). This compound was prepared according to the general procedures, 46.7 mg, 58% yield as a colorless oil. ^1^H NMR (400 MHz, Chloroform-*d*) δ 7.92 (d, *J* = 8.1 Hz, 2H), 7.76 (dd, *J* = 3.8, 1.2 Hz, 1H), 7.53 (dd, *J* = 5.0, 1.2 Hz, 1H), 7.28 (d, *J* = 7.6 Hz, 2H), 7.08 (dd, *J* = 5.0, 3.8 Hz, 1H), 4.44 (t, *J* = 6.0 Hz, 1H), 4.39-4.31 (m, 2H), 3.67 (s, 3H), 2.41 (s, 3H), 2.19–2.09 (m, 2H), 1.70 (t, *J* = 7.2 Hz, 2H), 0.96 (s, 6H). ^13^C NMR (101 MHz, Chloroform-*d*) δ 194.7, 170.9, 162.2, 144.5, 133.9, 133.5, 133.3, 132.3, 129.5, 128.9, 127.7, 62.1, 52.6, 49.9, 40.5, 40.1, 32.8, 27.1, 27.0, 21.7. HR-MS (ESI) C_22_H_27_SO_5_ [M + H]^+^: 403.1574, found: 403.1575.
6-methoxy-3,3-dimethyl-5-(4-methylbenzoyl)-6-oxohexyl furan-2-carboxylate (*39*). This compound was prepared according to the general procedures, 43.3 mg, 56% yield as a colorless oil. ^1^H NMR (400 MHz, Chloroform-*d*) δ 7.93–7.90 (m, 2H), 7.53-7.47(m, 1H), 7.29–7.26 (m, 2H), 7.21-7.17 (m, 1H), 7.15–7.06 (m, 1H), 4.48–4.33 (m, 3H), 3.66 (s, 3H), 2.41 (s, 3H), 2.22–2.07 (m, 2H), 1.73 (t, *J* = 7.1 Hz, 2H), 0.96 (d, *J* = 1.9 Hz, 6H). ^13^C NMR (101 MHz, Chloroform-*d*) δ 194.7, 170.9, 144.5, 134.4, 134.3, 133.4, 132.0, 129.5, 128.9, 123.9, 123.9, 117.1, 116.8, 62.3, 52.6, 49.9, 40.5, 40.0, 32.8, 27.1, 27.0, 21.7. HR-MS (ESI) C_22_H_27_O_6_ [M + H]^+^: 387.1802, found: 387.1810.
*Methyl-4-methyl-2-(4-methylbenzoyl)pentanoate* (**40**). This compound was prepared according to the general procedures, 31.8 mg, 64% yield as a colorless oil. ^1^H NMR (400 MHz, Chloroform-*d*) δ 7.90 (d, *J* = 8.0 Hz, 2H), 7.28 (d, *J* = 7.7 Hz, 2H), 4.43–4.39 (m, 1H), 3.68 (s, 3H), 2.42 (s, 3H), 1.99–1.82 (m, 2H), 1.67-1.56 (m, 1H), 0.96–0.91 (m, 6H). ^13^C NMR (101 MHz, Chloroform-*d*) δ 194.9, 170.8, 144.5, 133.7, 129.5, 128.7, 52.4, 52.1, 37.9, 26.4, 22.6, 22.3, 21.7. HR-MS (ESI) C_15_H_21_O_3_ [M + H]^+^: 249.1485, found: 249.1485.
*Methyl 2-(cyclopentylmethyl)-3-oxo-3-(p-tolyl)propanoate* (**41**). This compound was prepared according to the general procedures, 36.7 mg, 67% yield as a colorless oil. ^1^H NMR (400 MHz, Chloroform-*d*) δ 7.88 (d, *J* = 7.9 Hz, 2H), 7.26 (d, *J* = 7.6 Hz, 2H), 4.44–4.40 (m, 1H), 3.69 (s, 3H), 2.43 (s, 3H), 2.00–1.82 (m, 2H), 1.67-1.56 (m, 1H), 0.95-0.91 (m, 6H). ^13^C NMR (101 MHz, Chloroform-*d*) δ 195.0, 170.7, 144.5, 132.7, 129.5, 127.7, 52.3, 52.2, 37.8, 26.4, 22.5, 22.3, 21.6. HR-MS (ESI) C_17_H_23_O_3_ [M + H]^+^: 275.1642, found: 275.1645.
*Methyl 2-(cyclohexylmethyl)-3-oxo-3-(p-tolyl)propanoate* (**42**). This compound was prepared according to the general procedures, 41.4 mg, 72% yield as a colorless oil. ^1^H NMR (400 MHz, Chloroform-*d*) δ 7.91 (d, *J* = 8.0 Hz, 2H), 7.30 (d, *J* = 7.8 Hz, 2H), 4.44–4.39 (m, 1H), 3.67 (s, 3H), 2.43 (s, 3H), 2.00–1.82 (m, 2H), 1.68-1.57 (m, 1H), 0.97–0.92 (m, 6H). ^13^C NMR (101 MHz, Chloroform-*d*) δ 194.9, 170.8, 144.5, 134.1, 129.5, 128.7, 52.4, 52.1, 37.9, 26.4, 22.6, 22.3, 21.7. HR-MS (ESI) C_18_H_25_O_3_ [M + H]^+^: 289.1789, found: 289.1792.
*Methyl 2-(bicyclo[2.2.1]heptan-2-ylmethyl)-3-oxo-3-(p-tolyl)propanoate* (**43**). This compound was prepared according to the general procedures, 36.6 mg, 61% yield as a colorless oil. ^1^H NMR (400 MHz, Chloroform-*d*) δ 7.90 (d, *J* = 87.9 Hz, 2H), 7.28 (d, *J* = 7.8 Hz, 2H), 4.43-4.39 (m, 1H), 3.69 (s, 3H), 2.42 (s, 3H), 1.97–1.82 (m, 2H), 1.67–1.57 (m, 1H), 0.96–0.92 (m, 6H). ^13^C NMR (101 MHz, Chloroform-*d*) δ 194.9, 170.7, 144.6, 133.8, 129.6, 128.8, 52.4, 52.1, 38.0, 26.4, 22.6, 22.4, 21.7. HR-MS (ESI) C_19_H_25_O_3_ [M + H]^+^: 301.1798, found: 301.1781.

## 7. Mechanistic Investigations


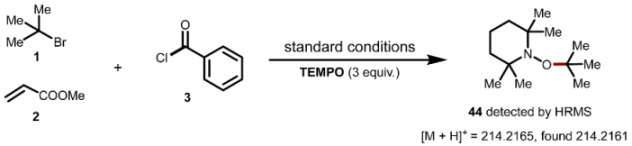


A dry 10 mL vial equipped with a Teflon-coated magnetic stir bar was charged with benzoyl chlorides (0.60 mmol, 3.0 equiv.), 2-bromo-2-methylpropanes (1.0 mmol, 5 equiv.), methyl acrylate (0.20 mmol, 1.0 equiv.), TBAB (2.0 equiv.), NiBr_2_•DME (10 mol%), dtbbpy (10 mol%), Zn (0.4 mmol, 2.0 equiv.), and TEMPO (0.6 mmol, 3.0 equiv.) were dissolved in MeCN (3.0 mL). The reaction mixture was stirred under N_2_ for 12 h. After the reaction was completed, the reaction mixture was analyzed by HRMS.

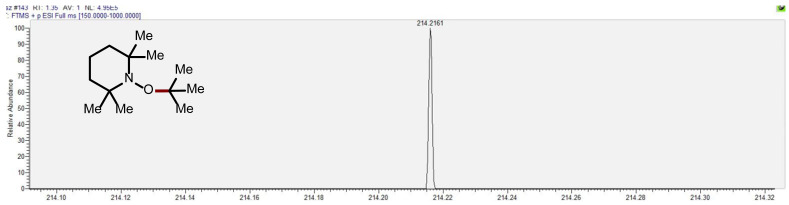




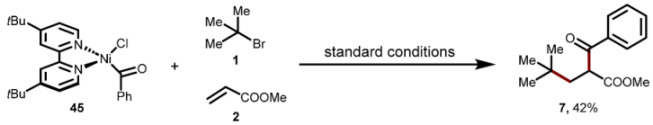


A dry 10 mL vial equipped with a Teflon-coated magnetic stir bar was charged with 2-bromo-2-methylpropanes (1.0 mmol, 5 equiv.), methyl acrylate (0.20 mmol, 1.0 equiv.), TBAB (2.0 equiv.), complex **45** (10 mol%), and Zn (0.4 mmol, 2.0 equiv.) were dissolved in MeCN (3.0 mL). The reaction mixture was stirred under N_2_ for 12 h. After the reaction was completed, the reaction mixture was analyzed by gas chromatography to obtain the yield of **7** using dodecane as an internal standard.


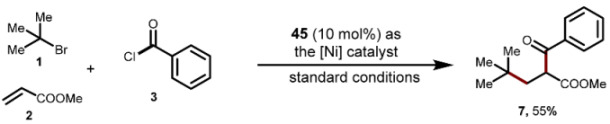


A dry 10 mL vial equipped with a Teflon-coated magnetic stir bar was charged with benzoyl chlorides (0.60 mmol, 3.0 equiv.), 2-bromo-2-methylpropanes (1.0 mmol, 5 equiv.), methyl acrylate (0.20 mmol, 1.0 equiv.), TBAB (2.0 equiv.), complex **45** (10 mol%), and Zn (0.4 mmol, 2.0 equiv.) were dissolved in MeCN (3.0 mL). The reaction mixture was stirred under N_2_ for 12 h. After the reaction was completed, the reaction mixture was analyzed by gas chromatography to obtain the yield of **7** using dodecane as an internal standard.

## Figures and Tables

**Table 1:** Optimization of reaction conditions ^[a]^.

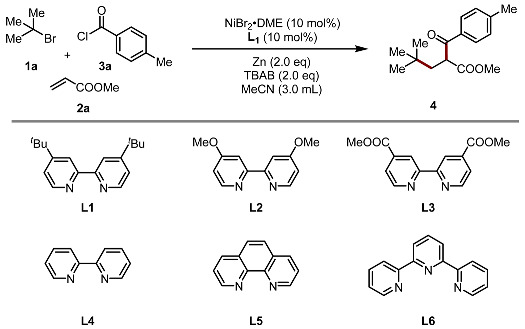		
Entry	Variation from “Standard Conditions”	4 [%] ^[b]^
1	none	78 ^[c]^
2	**L2** as ligand	47
3	**L3** as ligand	12
4	**L4** as ligand	55
5	**L5** as ligand	10
6	**L6** as ligand	0
7	THF as solvent	trace
8	DMA as solvent	24
9	Mn powder as a reducing agent	32
10	w/o NiBr_2_•DME	0
11	w/o TBAB	12
12	w/o ligand	0

^[a]^ Reaction conditions: **1a** (1.0 mmol), **2a** (0.2 mmol), **3a** (0.6 mmol), NiBr_2_•DME (10 mol%), **L** (10 mol%), TBAB (2.0 equiv.), Zn (2.0 equiv.), MeCN (3.0 mL), 12 h, rt, nitrogen. ^[b]^ GC yields using dodecane as an internal standard. ^[c]^ Isolated yields.

## Data Availability

The data that support the findings of this study are available in the Supporting Information of this article.

## Supplementary Materials

The general experimental details, optimization of the reaction condition and NMR spectra can be downloaded at: https://www.mdpi.com/article/10.3390/molecules29184295/s1.

## References

[1] Kurth M.C., Adler C.H., St. Hilaire M., Singer C., Waters C., Le Witt P., Chernik D.A., Dorflinger E.E., Yoo K. (1997). Tolcapone Improves Motor Function and Reduces Levodopa Requirement in Patients with Parkinson’s Disease Experiencing Motor Fluctuations: A Multicenter, Double-Blind, Randomized, Placebo-Controlled Trial. Neurology.

[2] Walter M.W. (2002). Structure-based design of agrochemicals. Nat. Prod. Rep..

[3] Koh K.K., Han S.H., Quon M.J., Yeal Ahn J., Shin E.K. (2005). Beneficial Effects of Fenofibrate to Improve Endothelial Dysfunction and Raise Adiponectin Levels in Patients With Primary Hypertriglyceridemia. Diabetes Care.

[4] Rong Z.-Q., Lim H.N., Dong G. (2018). Intramolecular Acetyl Transfer to Olefins by Catalytic C-C Bond Activation of Unstrained Ketones. Angew. Chem. Int. Ed..

[5] Ouyang X.-H., Song R.-J., Li J.-H. (2014). Iron-Catalyzed Oxidative 1,2-Carboacylation of Activated Alkenes with Alcohols: A Tandem Route to 3-(2-Oxoethyl)-indolin-2-ones. Eur. J. Org. Chem..

[6] Walker J.A., Vickerman K.L., Humke J.N., Stanley L.M. (2017). Ni-Catalyzed Alkene Carboacylation via Amide C-N Bond Activation. J. Am. Chem. Soc..

[7] Deng L., Fu Y., Lee S.Y., Wang C., Liu P., Dong G. (2019). Kinetic Resolution via Rh-Catalyzed C-C Activation of Cyclobutanones at Room Temperature. J. Am. Chem. Soc..

[8] Oguma K., Miura M., Satoh T., Nomura M. (2002). Rhodium Catalyzed Coupling of Sodium Tetraphenylborate with Acid Anhydrides in the Presence or Absence of Norbornene. J. Organomet. Chem..

[9] Giri R., KC S. (2018). Strategies toward Dicarbofunctionalization of Unactivated Olefins by Combined Heck Carbometalation and Cross-Coupling. J. Org. Chem..

[10] Mizutani K., Shinokubo H., Oshima K. (2003). Cobalt-Catalyzed Three-Component Coupling Reaction of Alkyl Halides, 1,3-Dienes, and Trimethylsilylmethylmagnesium Chloride. Org. Lett..

[11] Terao J., Kato Y., Kambe N. (2008). Titanocene-Catalyzed Regioselective Alkylation of Styrenes with Grignard Reagents Using β-Bromoethyl Ethers, Thioethers, or Amines. Chem.—Asian J..

[12] Wu L., Wang F., Wan X., Wang D., Chen P., Liu G. (2017). Asymmetric Cu-Catalyzed Intermolecular Trifluoromethylarylation of Styrenes: Enantioselective Arylation of Benzylic Radicals. J. Am. Chem. Soc..

[13] Lin J.-S., Li T.-T., Liu J.-R., Jiao G.-Y., Gu Q.-S., Cheng J.-T., Guo Y.-L., Hong X., Liu X.-Y. (2019). Cu/Chiral Phosphoric Acid-Catalyzed Asymmetric Three-Component Radical-Initiated 1,2-Dicarbofunctionalization of Alkenes. J. Am. Chem. Soc..

[14] Klauck F.J.R., Yoon H., James M.J., Lautens M., Glorius F. (2019). Visible-Light-MediatedDeaminative Three-Component Dicarbofunctionalization of Styrenes with Benzylic Radicals. ACS Catal..

[15] Mega R.S., Duong V.K., Noble A., Aggarwal V.K. (2020). Decarboxylative Conjunctive Cross-coupling of Vinyl Boronic Esters using Metallaphotoredox Catalysis. Angew. Chem. Int. Ed..

[16] Lei G., Zhang H., Chen B., Xu M., Zhang G. (2020). Copper-Catalyzed Enantioselective Arylalkynylation of Alkenes. Chem. Sci..

[17] Sun S.-Z., Duan Y., Mega R.S., Somerville R.J., Martin R. (2020). Site-Selective 1,2-Dicarbofunctionalization of Vinyl Boronates through Dual Catalysis. Angew. Chem. Int. Ed..

[18] Yang T., Chen X., Rao W., Koh M.J. (2020). Broadly Applicable Directed Catalytic Reductive Difunctionalization of Alkenyl Carbonyl Compounds. Chem.

[19] Zheng Y.-L., Newman S.G. (2019). Nickel-Catalyzed Domino Heck-Type Reactions Using Methyl Esters as Cross-Coupling Electrophiles. Angew. Chem. Int. Ed..

[20] Xu S., Wang K., Kong W. (2019). Ni-Catalyzed Reductive Arylacylation of Alkenes toward Carbonyl-Containing Oxindoles. Org. Lett..

[21] Wang L., Wang C. (2020). Nickel-Catalyzed Three-Component Reductive Alkylacylation of Electron-Deficient Activated Alkenes. Org. Lett..

[22] Kadam A.A., Metz T.L., Qian Y., Stanley L.M. (2019). Ni-Catalyzed Three-Component Alkene Carboacylation Initiated by Amide C-N Bond Activation. ACS Catal..

[23] Ping Y., Kong W. (2020). Ni-Catalyzed Reductive Difunctionalization of Alkenes. Synthesis.

[24] Luo Y.-C., Xu C., Zhang X. (2020). Nickel-Catalyzed Dicarbofunctionalization of Alkenes. Chin. J. Chem..

[25] Derosa J., Apolinar O., Kang T., Tran V.T., Engle K.M. (2020). Recent Developments in NickelCatalyzed Intermolecular Dicarbofunctionalization of Alkenes. Chem. Sci..

[26] Zhao X., Tu H.-Y., Guo L., Zhu S., Qing F.-L., Chu L. (2018). Intermolecular selective carboacylation of alkenes via nickel-catalyzed reductive radical relay. Nat. Commun..

[27] Gu J.-W., Min Q.-Q., Yu L.-C., Zhang X. (2016). Tandem Difluoroalkylation-Arylation of Enamides Catalyzed by Nickel. Angew. Chem. Int. Ed..

[28] Shrestha B., Basnet P., Dhungana R.K., KC S., Thapa S., Sears J.M., Giri R. (2017). Ni-Catalyzed Regioselective 1,2-Dicarbofunctionalization of Olefins by Intercepting Heck Intermediates as Imine-Stabilized Transient Metallacycles. J. Am. Chem. Soc..

[29] Derosa J., Tran V.T., Boulous M.N., Chen J.S., Engle K.M. (2017). Nickel-Catalyzed β, γ-Dicarbofunctionalization of Alkenyl Carbonyl Compounds via Conjunctive Cross-Coupling. J. Am. Chem. Soc..

[30] Basnet P., Dhungana R.K., Thapa S., Shrestha B., KC S., Sears J.M., Giri R. (2018). Ni-Catalyzed Regioselective β, δ-Diarylation of Unactivated Olefins in Ketimines via Ligand-Enabled Contraction of Transient Nickellacycles: Rapid Access to Remotely Diarylated Ketones. J. Am. Chem. Soc..

[31] KC S., Dhungana R.K., Shrestha B., Thapa S., Khanal N., Basnet P., Lebrun R.W., Giri R. (2018). Ni-Catalyzed Regioselective Alkylarylation of Vinylarenes via C(sp^3^)-C(sp^3^)/C(sp^3^)-C(sp^2^) Bond Formation and Mechanistic Studies. J. Am. Chem. Soc..

[32] Gao P., Chen L.-A., Brown M.K. (2018). Nickel-Catalyzed Stereoselective Diarylation of Alkenylarenes. J. Am. Chem. Soc..

[33] Fu L., Zhou S., Wan X., Chen P., Liu G. (2018). Enantioselective Trifluoromethylalkynylation of Alkenes via CopperCatalyzed Radical Relay. J. Am. Chem. Soc..

[34] Luridiana A., Mazzarella D., Capaldo L., Rincón J.A., García-Losada P., Mateos C., Frederick M.O., Nuño M., Buma W.J., Noël T. (2022). The Merger of Benzophenone HAT Photocatalysis and Silyl RadicalInduced XAT Enables Both Nickel-Catalyzed Cross-Electrophile Coupling and 1,2-Dicarbofunctionalization of Olefins. ACS Catal..

[35] Derosa J., Kleinmans R., Tran V.T., Karunananda M.K., Wisniewski S.R., Eastgate M.D., Engle K.M. (2018). Nickel-Catalyzed 1,2-Diarylation of Simple Alkenyl Amides. J. Am. Chem. Soc..

[36] Li W., Boon J.K., Zhao Y. (2018). Nickel-Catalyzed Difunctionalization of Allyl moieties using Organoboronic Acids and Halides with Divergent Regioselectivities. Chem. Sci..

[37] GarcíaDomínguez A., Mondal R., Nevado C. (2019). Dual Photoredox/NickelCatalyzed Three-Component Carbofunctionalization of Alkenes. Angew. Chem. Int. Ed..

[38] Campbell M.W., Compton J.-S., Kelly C.B., Molander G.A. (2019). Three-Component Olefin Dicarbofunctionalization Enabled by Nickel/Photoredox Dual Catalysis. J. Am. Chem. Soc..

[39] Kc S., Dhungana R.K., Khanal N., Giri R. (2020). Nickel Catalyzed α-Carbonylalkylarylation of Vinylarenes: Expedient Access to γ,γ-Diarylcarbonyl and Aryltetralone Derivatives. Angew. Chem. Int. Ed..

[40] Xu C., Cheng R., Luo Y.-C., Wang M.-K., Zhang X. (2020). trans-Selective Aryldifluoroalkylation of Endocyclic Enecarbamates and Enamides via Nickel Catalysis. Angew. Chem. Int. Ed..

[41] García-Domínguez A., Li Z., Nevado C. (2017). Nickel-Catalyzed Reductive Dicarbofunctionalization of Alkenes. J. Am. Chem. Soc..

[42] Shu W., García-Domínguez A., Quiros M.T., Mondal R., Cárdenas D.J., Nevado C. (2019). Ni-Catalyzed Reductive Dicarbofunctionalization of Nonactivated Alkenes: Scope and Mechanistic Insights. J. Am. Chem. Soc..

[43] Guo L., Tu H.-Y., Zhu S., Chu L. (2019). Selective, Intermolecular Alkylarylation of Alkenes via Photoredox/Nickel Dual Catalysis. Org. Lett..

[44] Tu H.-Y., Wang F., Huo L.-P., Li Y., Zhu S., Zhao X., Li H., Qing F.-L., Chu L. (2020). Enantioselective Three-Component Fluoroalkylarylation of Unactivated Olefins through Nickel-Catalyzed Cross-Electrophile Coupling. J. Am. Chem. Soc..

[45] Wei X., Shu W., García-Domínguez A., Merino E., Nevado C. (2020). Asymmetric Ni-Catalyzed Radical Relayed Reductive Coupling. J. Am. Chem. Soc..

[46] Wang X.-X., Lu X., He S.-J., Fu Y. (2020). Nickel-Catalyzed Three-Component Olefin Reductive Dicarbofunctionalization to Access Alkylborates. Chem. Sci..

[47] Wang Z.K., Wang Y.P., Rao Z.W., Liu C.-Y., Pan X.-H., Guo L. (2023). General Method for Selective Three-Component Carboacylation of Alkenes via Visible-Light Dual Photoredox/Nickel Catalysis. Org. Lett..

[48] Fan H., Wang B., Wu T., Kang Q., Wang H., Sun J., Wei X. (2024). Ball-milling-enabled nickel-catalyzed radical relayed reductive cross-coupling. Cell Rep. Phys. Sci..

