# Supplementary material for: Nickel-Catalyzed Three-Component 1,2-Carboacylation of Alkenes
Source: Molecules. 2024 Sep 10;29(18):4295. doi: 10.3390/molecules29184295 (PMC11433782; doi:10.3390/molecules29184295)
Supplement: Supplementary file 1 [file molecules-29-04295-s001.zip › molecules-3180812-supplementary.pdf]

Supplementary Information for

Nickel-Catalyzed Three-Component 1,2-Carboacylation of Alkenes

Shengzhou Jin [3], Lanfen Wang [1], Yinggang Jia [1], Wenbo Ma [2,*] and Dingyi Wang [1,*]

[1] College of Sciences, Northeastern University, Shenyang 110004, China
[2] Antibiotics Research and Re-Evaluation Key Laboratory of Sichuan Province, Sichuan Industrial Institute of Antibiotics, National Base for International Science and Technology Cooperation of Chengdu University, Chengdu University, Chengdu 610106, China
[3] Hubei Key Laboratory of Pollutant Analysis & Reuse Technology, College of Chemistry and Chemical Engineering, Hubei Normal University, Huangshi 435002, China
* Correspondence: wenboma@hotmail.com (W.M.); wangdingyi@mail.neu.edu.cn (D.W.)

TABLE OF CONTENTS

1. General Experimental Details 3

2. Optimization of the Reaction Condition 3

3. NMR Spectra 5

1. General Experimental Details

All new compounds were fully characterized. Compounds were visualized by exposure to UV-light. All reactions and manipulations involving air- or moisture-sensitive compounds were performed using standard Schlenk techniques or in a glovebox. Toluene was purified using Pure Solv MD-5 solvent purification system, from Innovative Technology, Inc., by passing the solvent through two activated alumina columns after purging with argon. ^{1}H, $^{13}C$and ^{19}F NMR spectra were recorded on a Bruker AVANCE III 400 MHz or 500 MHz spectrometer. Chemical shifts (δ values) were reported in ppm with $CDCl_3$ (7.26 and 77.16 ppm for ^{1}H and ^{13}C respectively). Mass spectra were conducted at Agilent 6540 Ultra-High-Definition (UHD) Accurate-Mass Quadrupole Time-of-Flight (Q-TOF) liquid chromatography/mass spectrometry (LC/MS) system and Thermo Scientific TRACE 1300 ISQ LT gas chromatography/mass spectrometry (GC/MS) system. Unless otherwise noted, materials obtained from commercial suppliers were used without further purification.

2. Optimization of the Reaction Condition

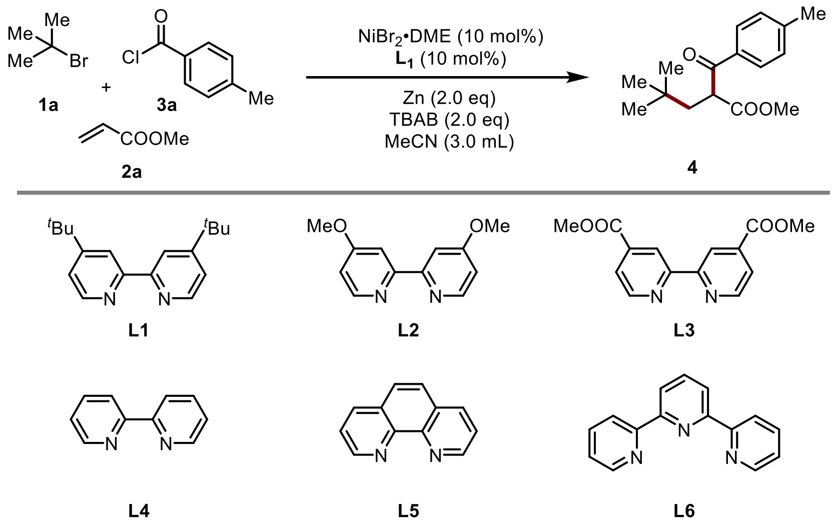

Entry	Variation from "standard conditions"	4 [%][b]
1	none	78[c]
2	**L2** as ligand	47

3	**L3** as ligand	12
4	**L4** as ligand	55
5	**L5** as ligand	10
6	**L6** as ligand	0
7	THF as solvent	trace
8	DMA as solvent	24
9	Mn powder as a reducing agent	32
10	5 mol% of $NiBr_2$·DME and 5 mol% of dtbbpy	65
11	12 mol% of $NiBr_2$·DME and 12 mol% of dtbbpy	77
12	Zn (3.0 equiv.)	76
13	80 °C	62
14	w/o $NiBr_2$·DME	0
15	w/o TBAB	12
16	w/o ligand	0

*[a]*Reaction conditions: **1a** (1.0 mmol), **2a** (0.2 mmol), **3a** (0.6 mmol), $NiBr_2$•DME (10 mol%), **L** (10 mol%), TBAB (2.0 equiv.), Zn (2.0 equiv.), MeCN (3.0 mL), 12 h, rt, nitrogen. *[b]*GC yields using dodecane as an internal standard. *[c]*isolated yields.

3. NMR Spectra

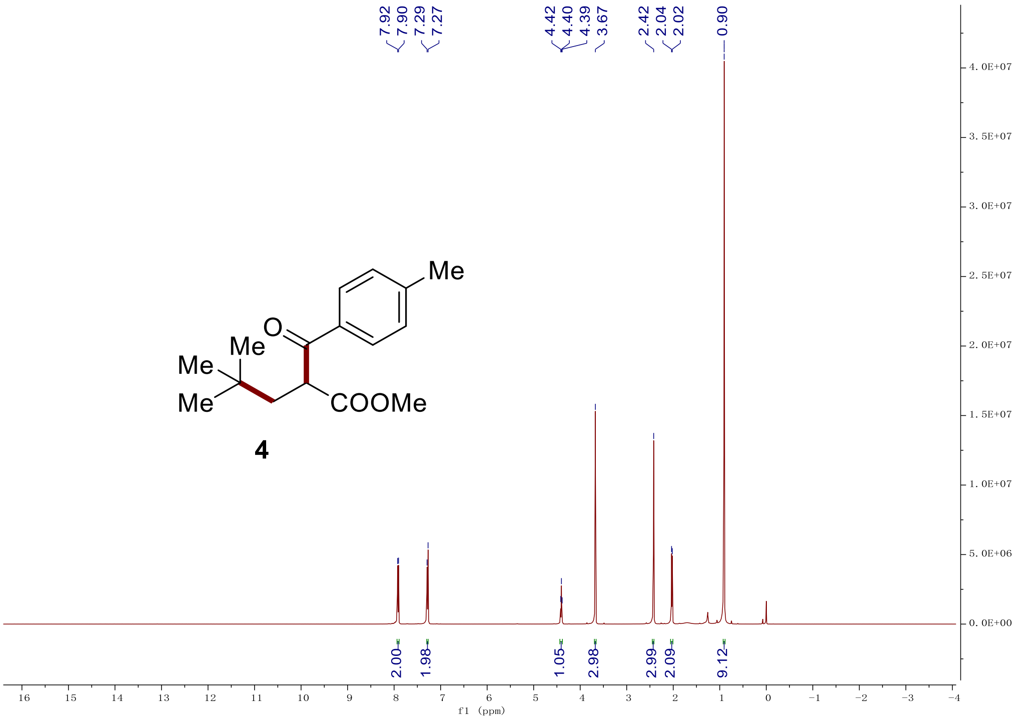

Figure S1. ^{1}H NMR of 4

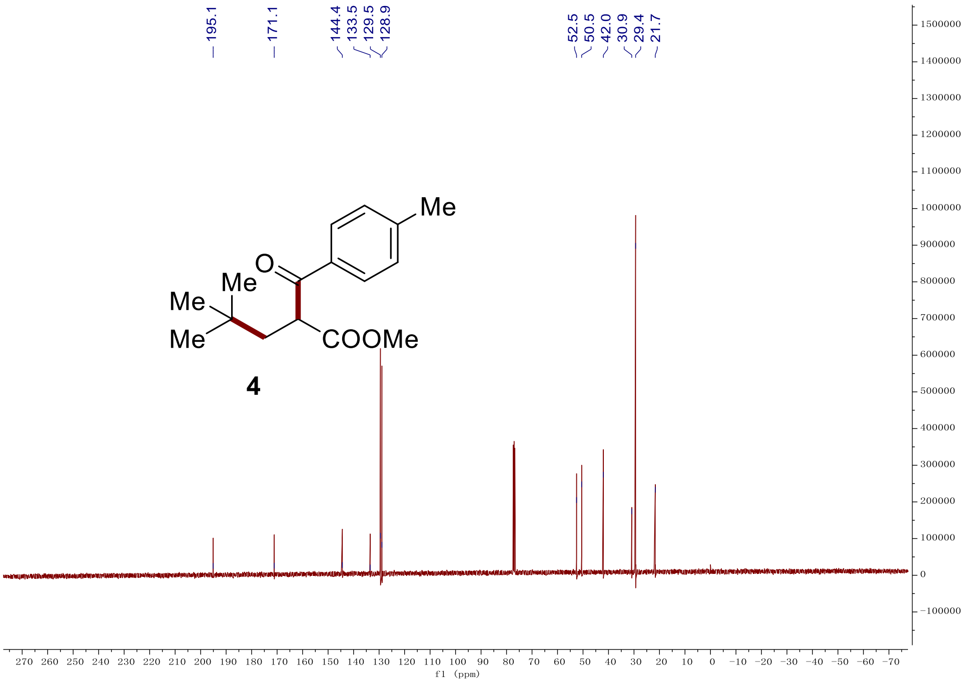

Figure S2. ^{13}C NMR of 4

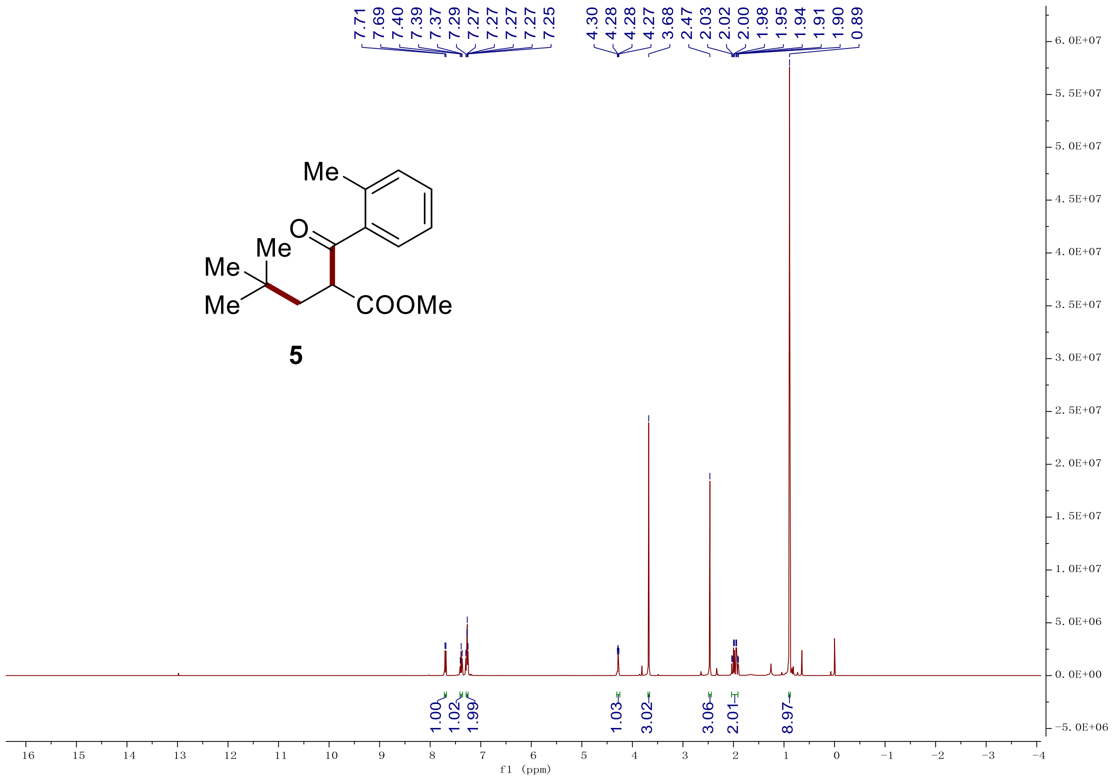

Figure S3. ^{1}H **NMR of 5**

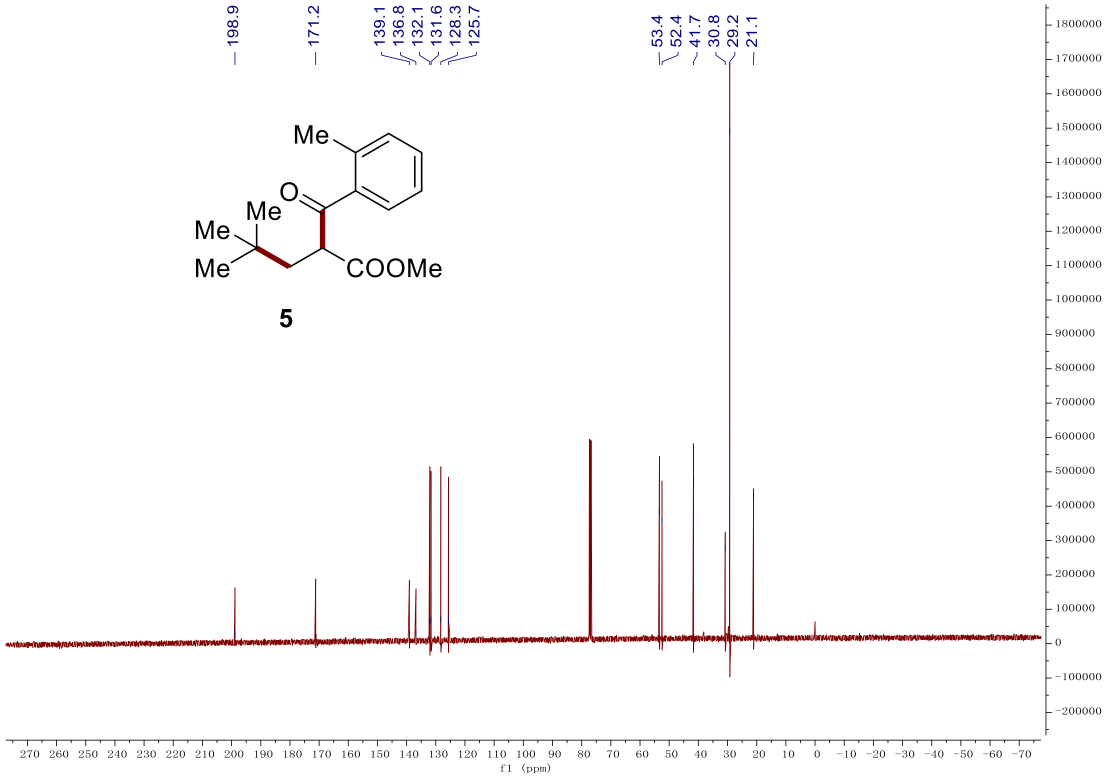

Figure S4. ^{13}C **NMR of 5**

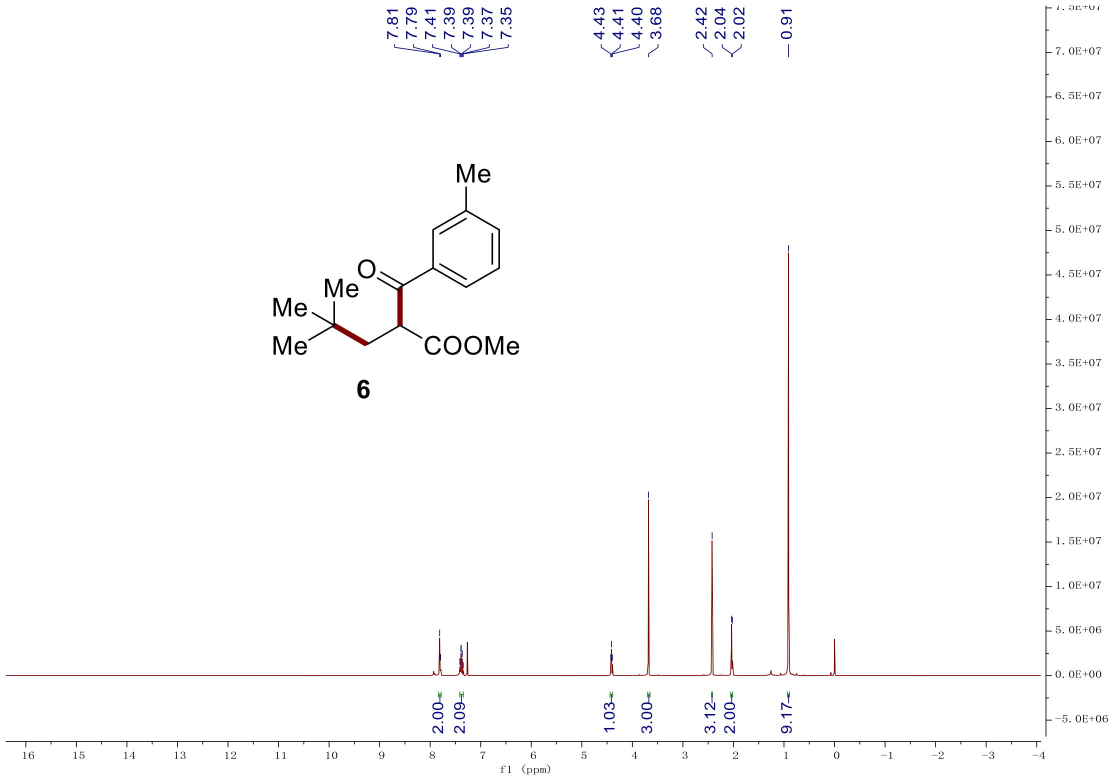

Figure S5. ^{1}H NMR of 6

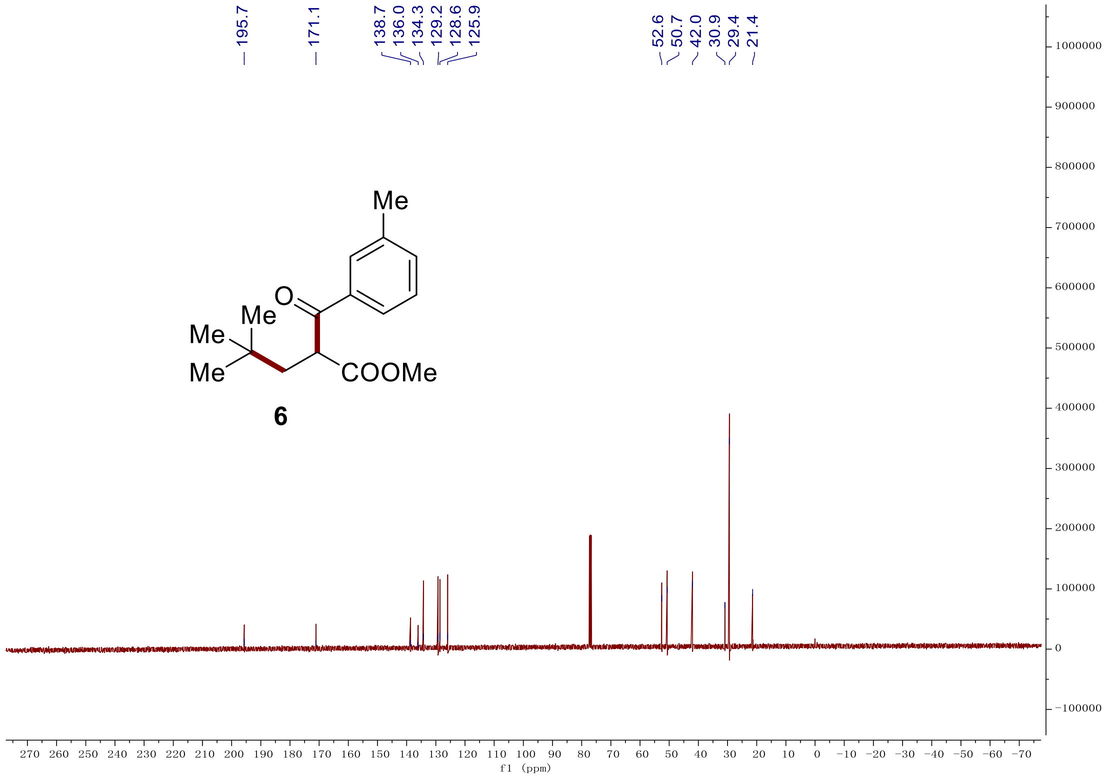

Figure S6. ^{13}C NMR of 6

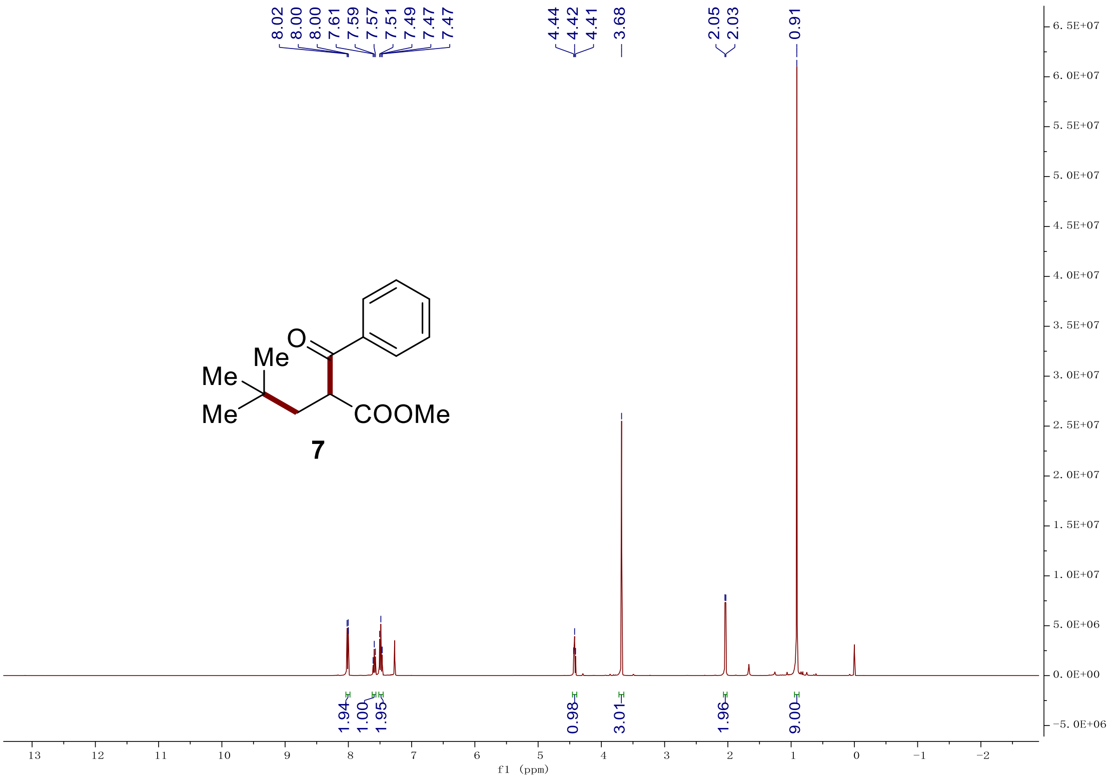

Figure S7. ^{1}**H NMR of 7**

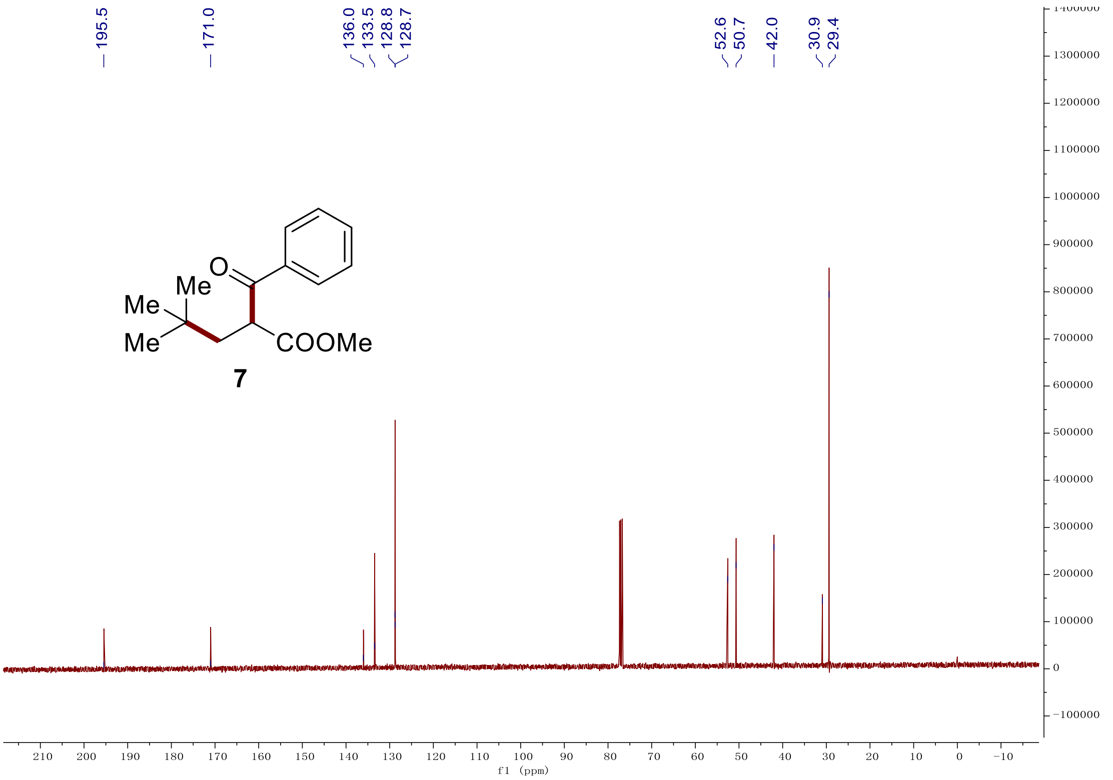

Figure S8. ^{13}**C NMR of 7**

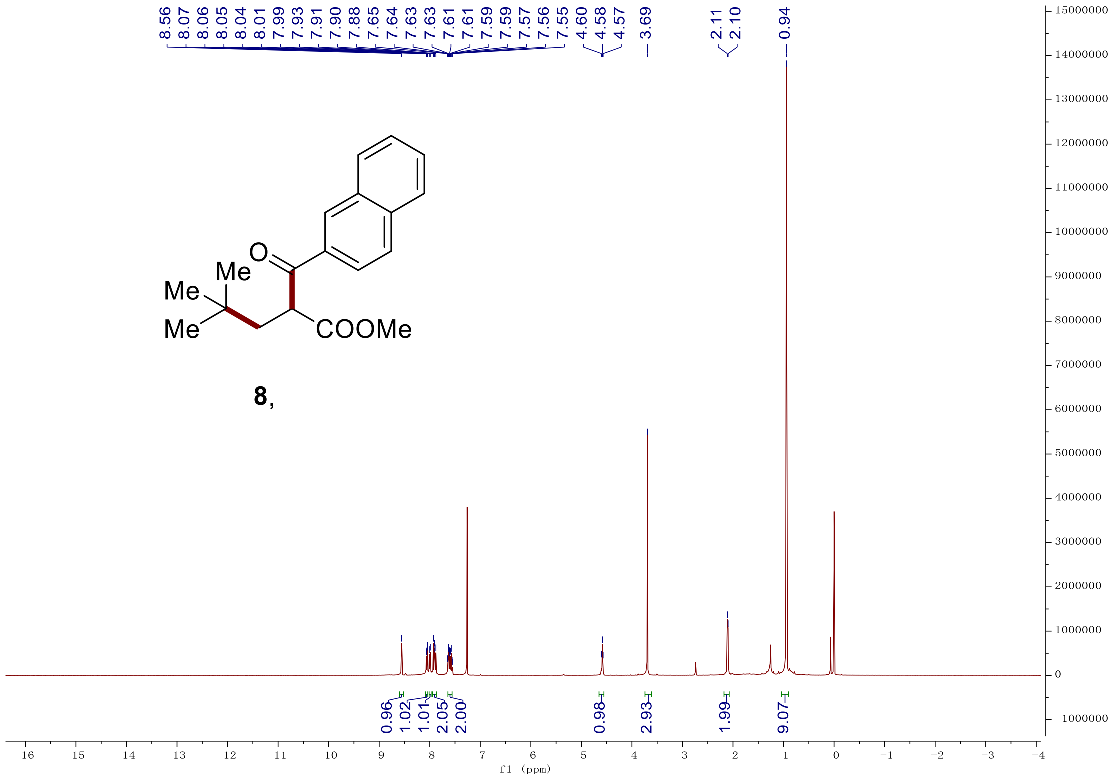

Figure S9. ^{1}H NMR of 8

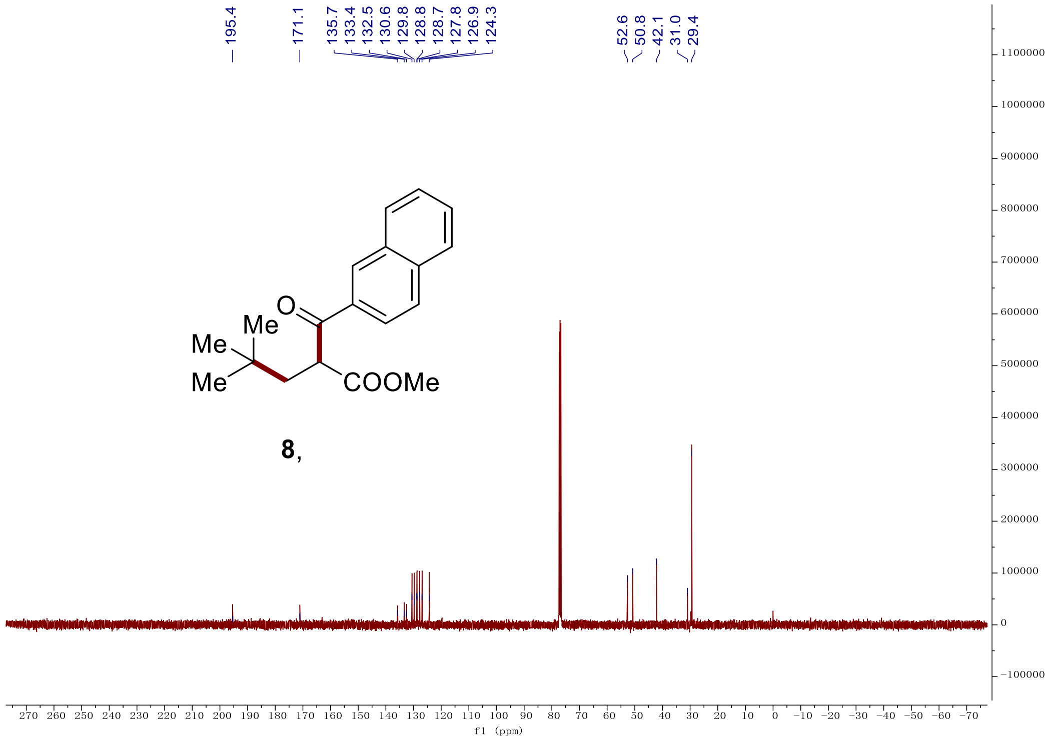

Figure S10. ^{13}C NMR of 8

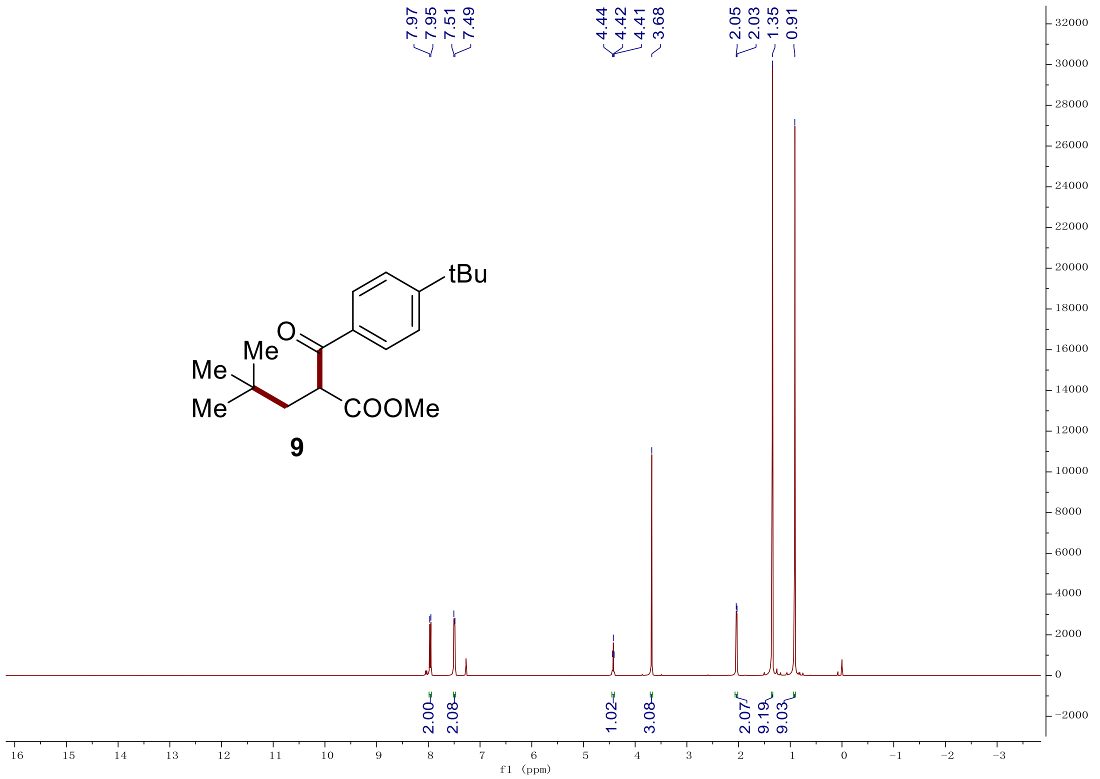

Figure S11. ^{1}**H NMR of 9**

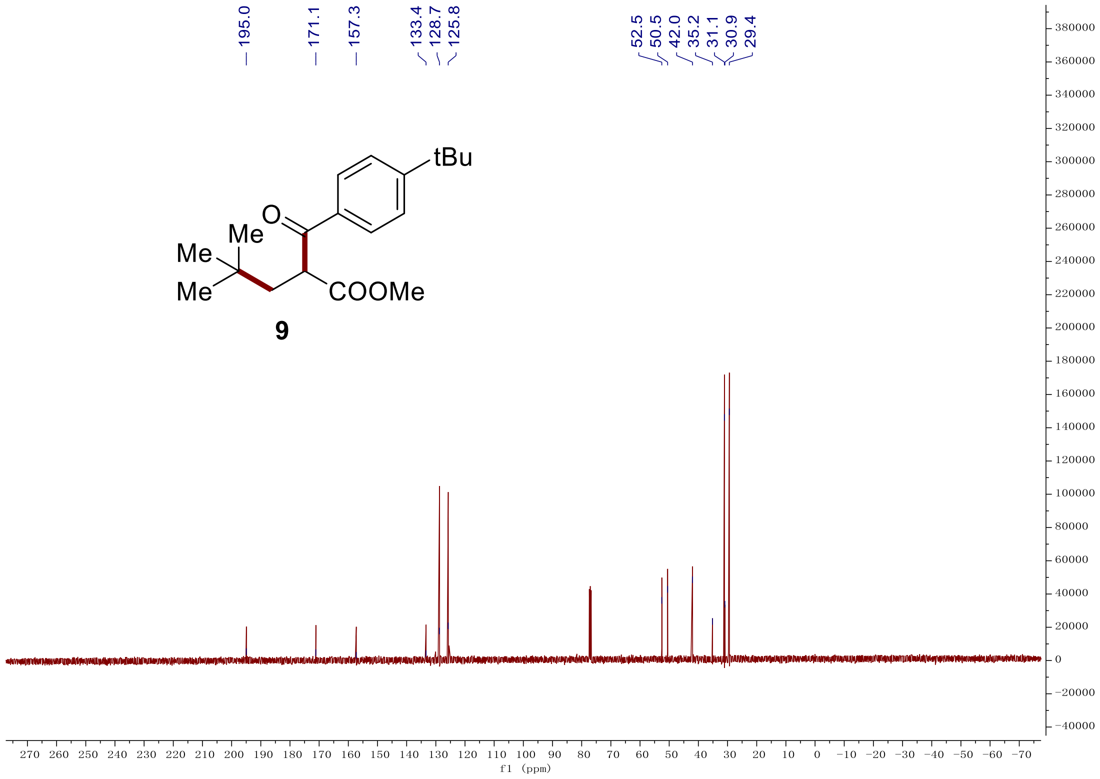

Figure S12. ^{13}**C NMR of 9**

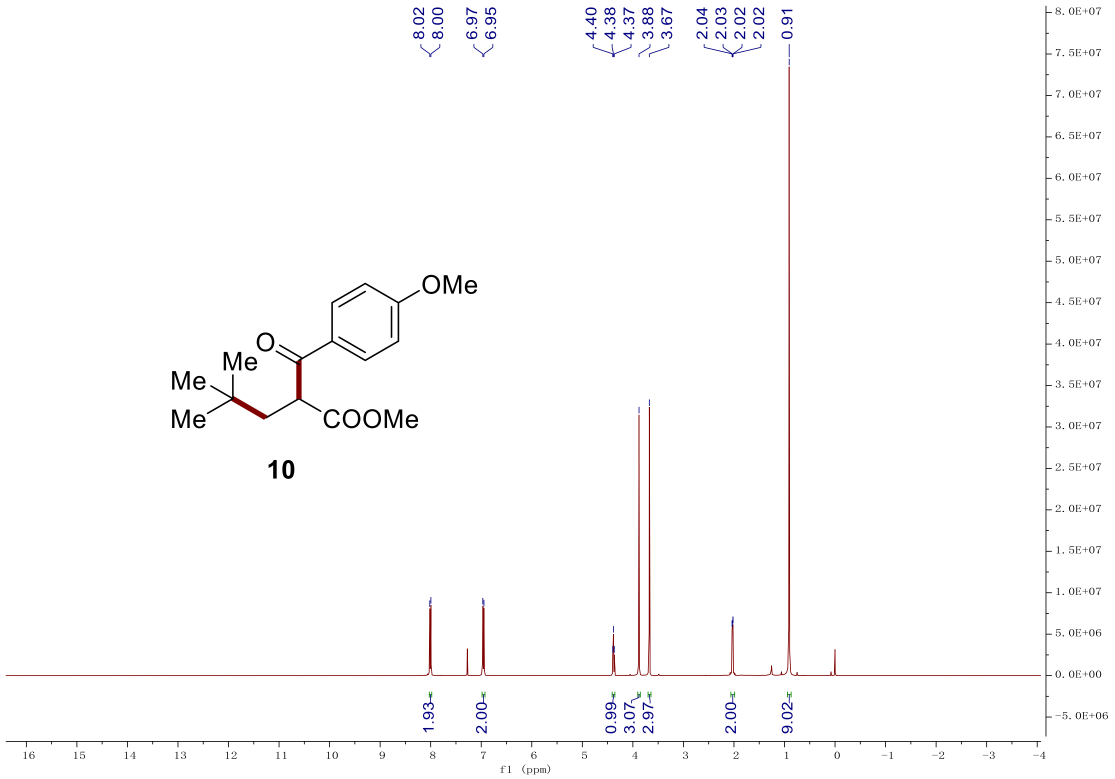

Figure S13. ^{1}H NMR of 10

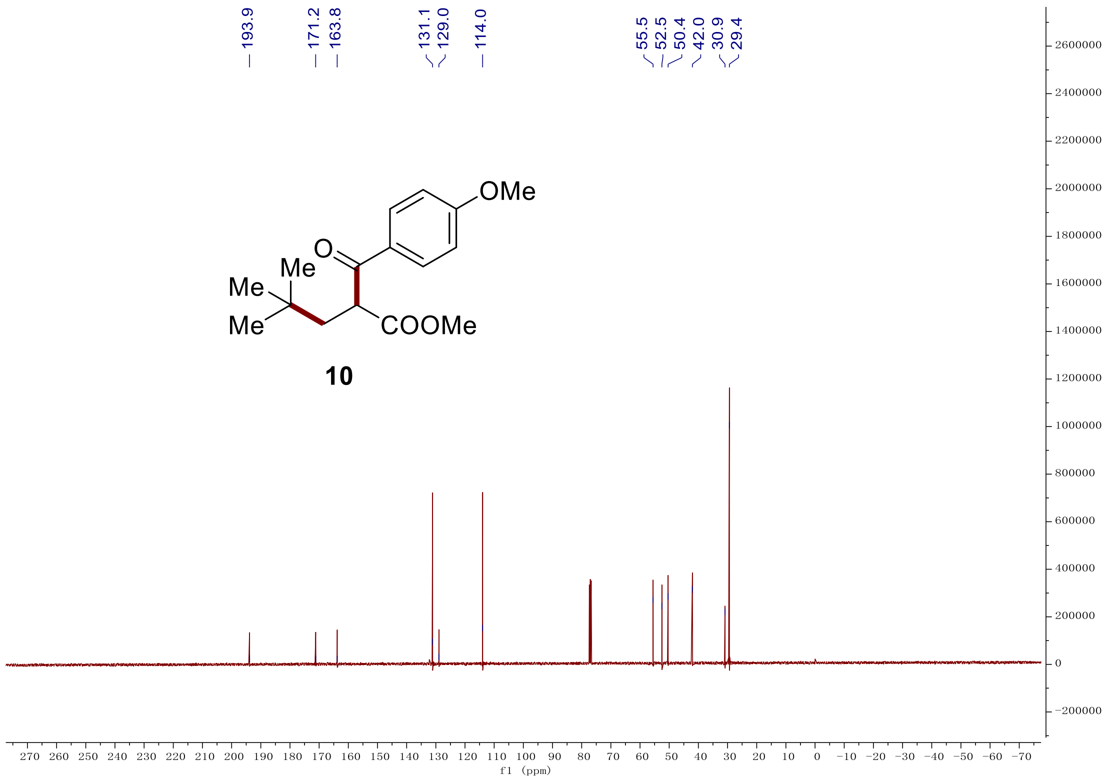

Figure S14. ^{13}C NMR of 10

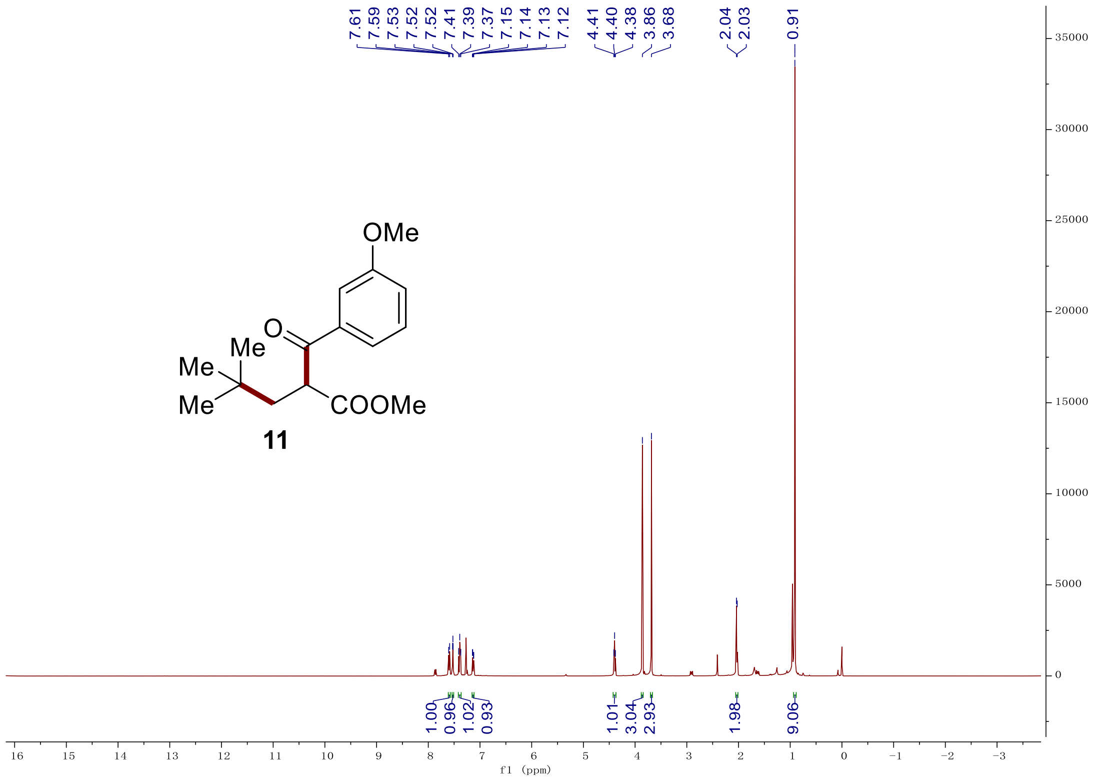

Figure S15. ^{1}**H NMR of 11**

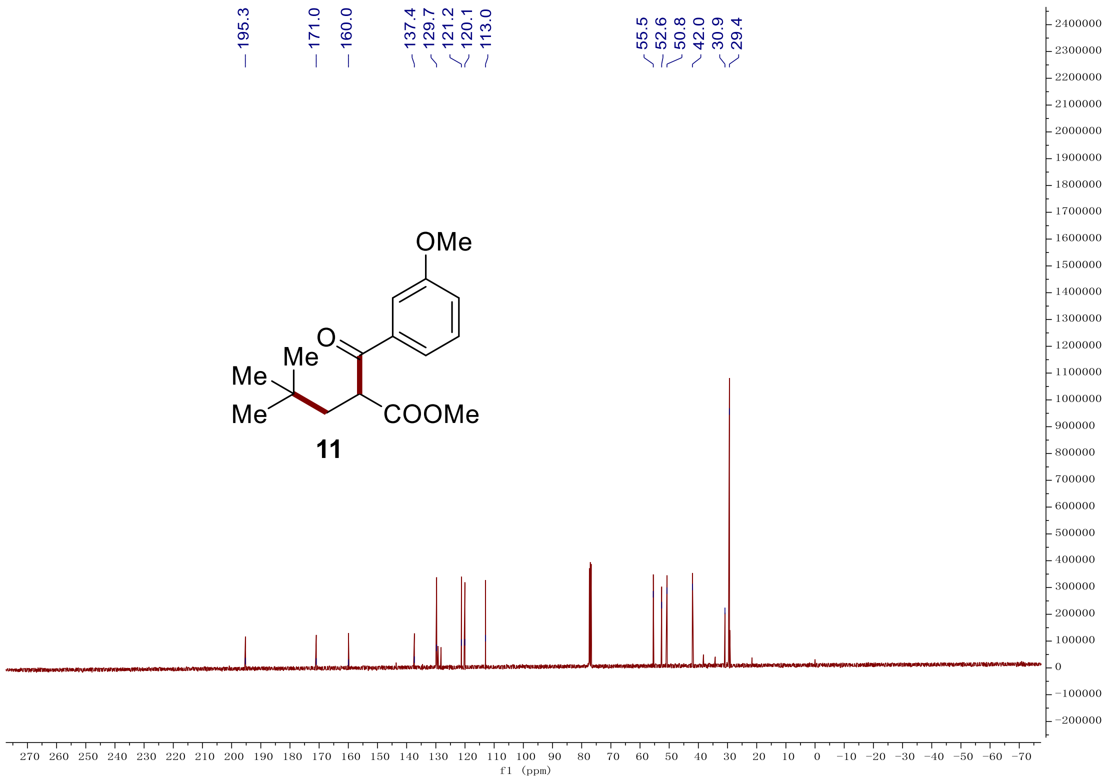

Figure S16. ^{13}**C NMR of 11**

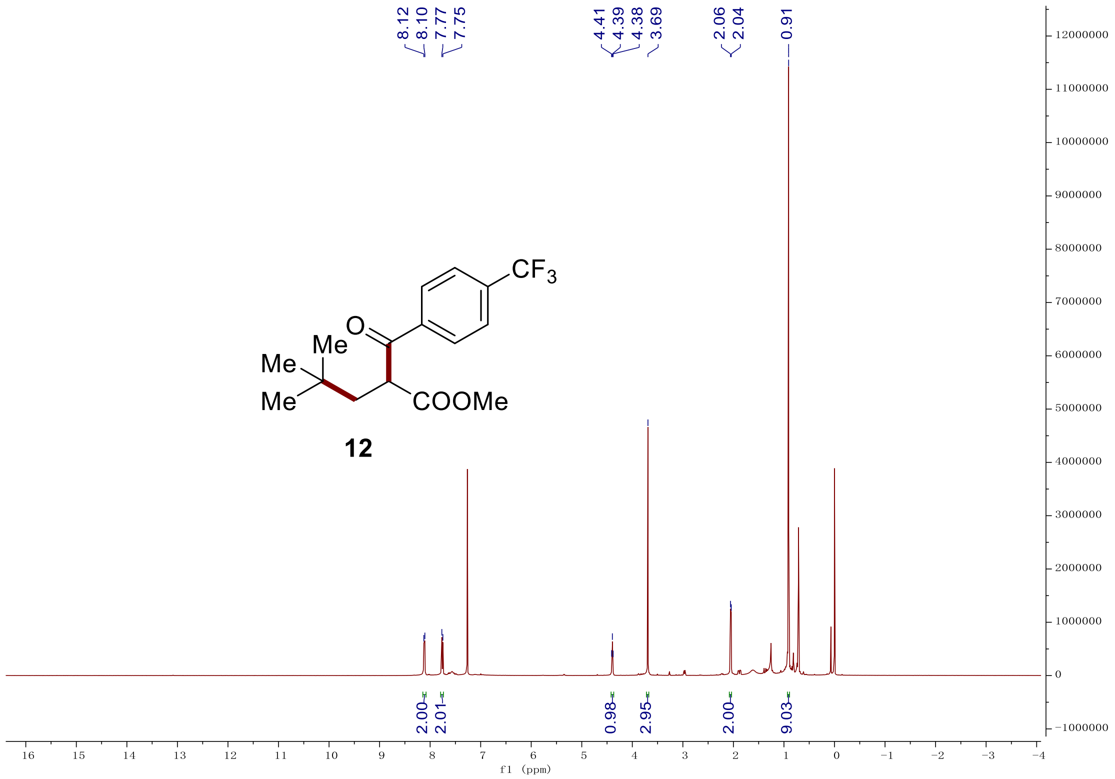

Figure S17. ^{1}H NMR of 12

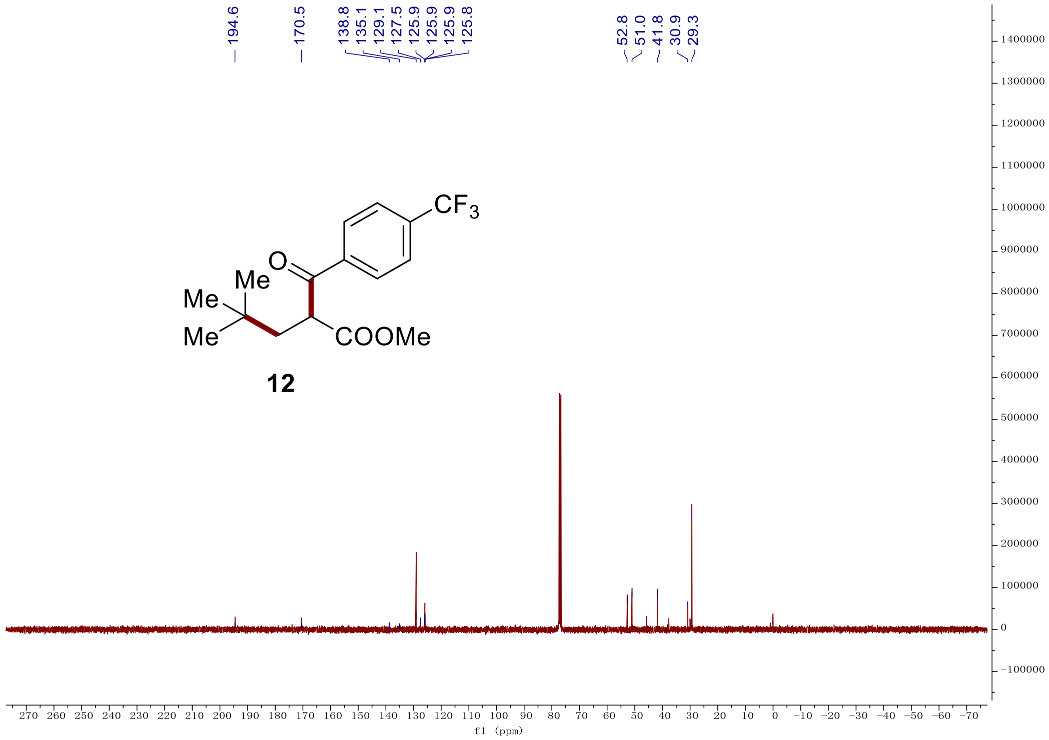

Figure S18. ^{13}C NMR of 12

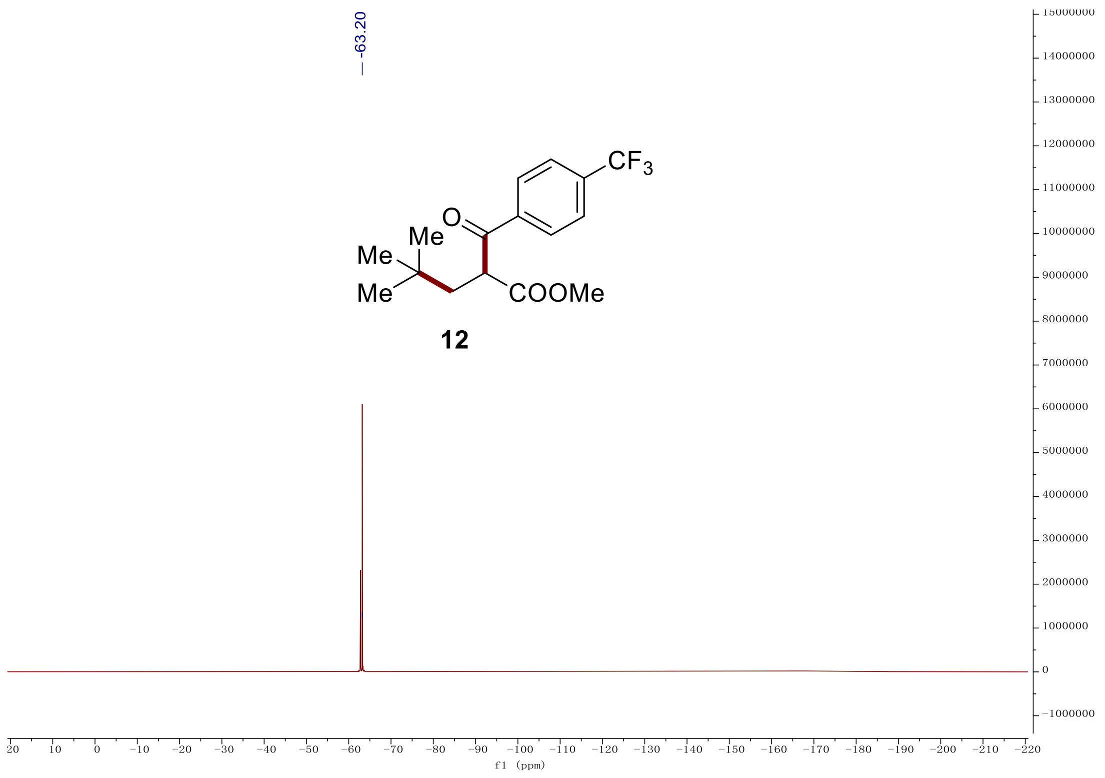

Figure S19. ^{19}F NMR of 12

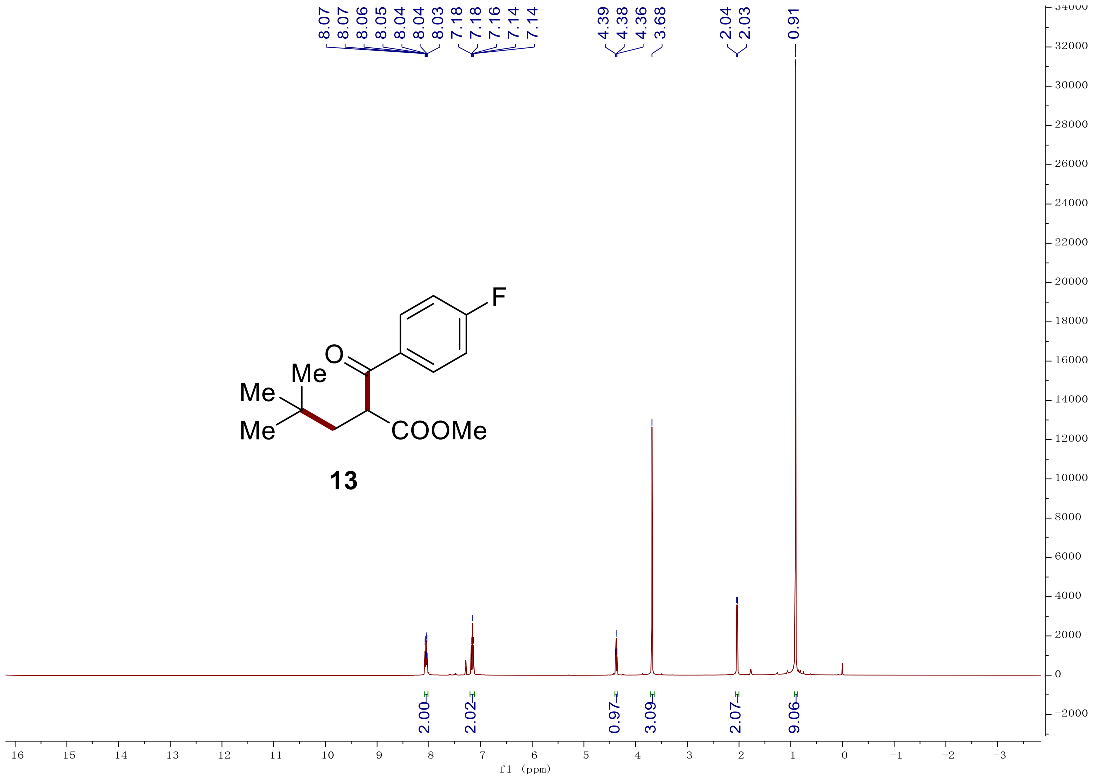

Figure S20. ^{1}H NMR of 13

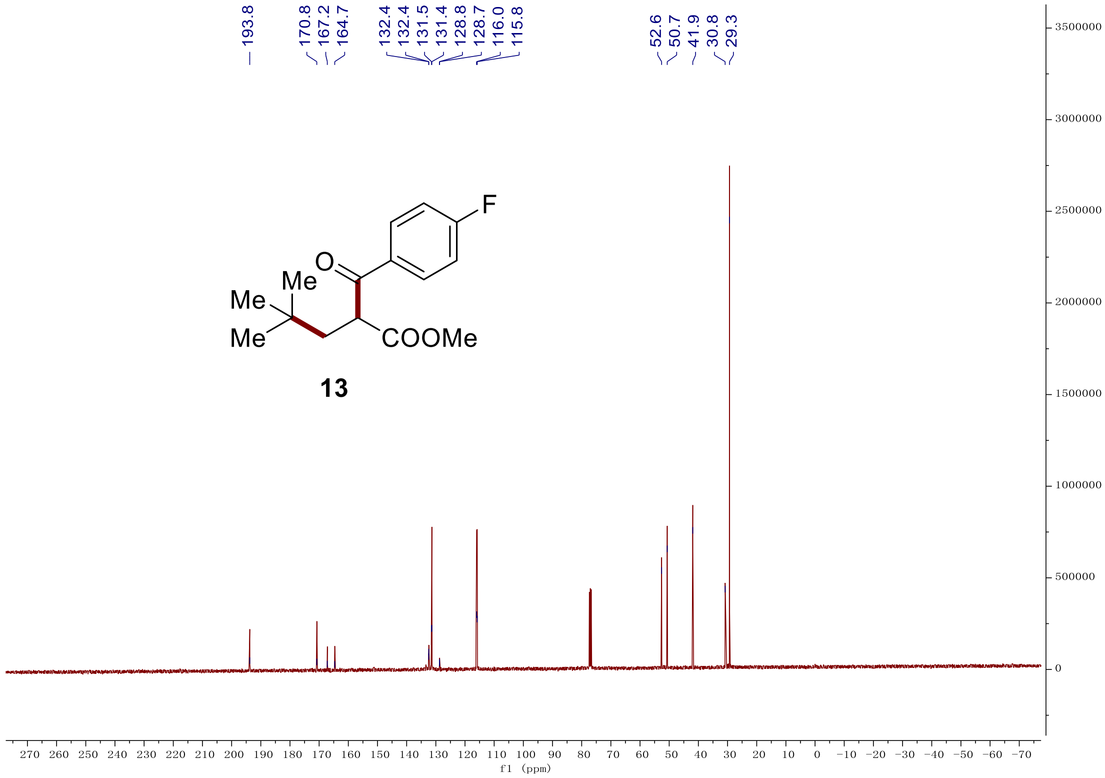

Figure S21. ^{13}C NMR of 13

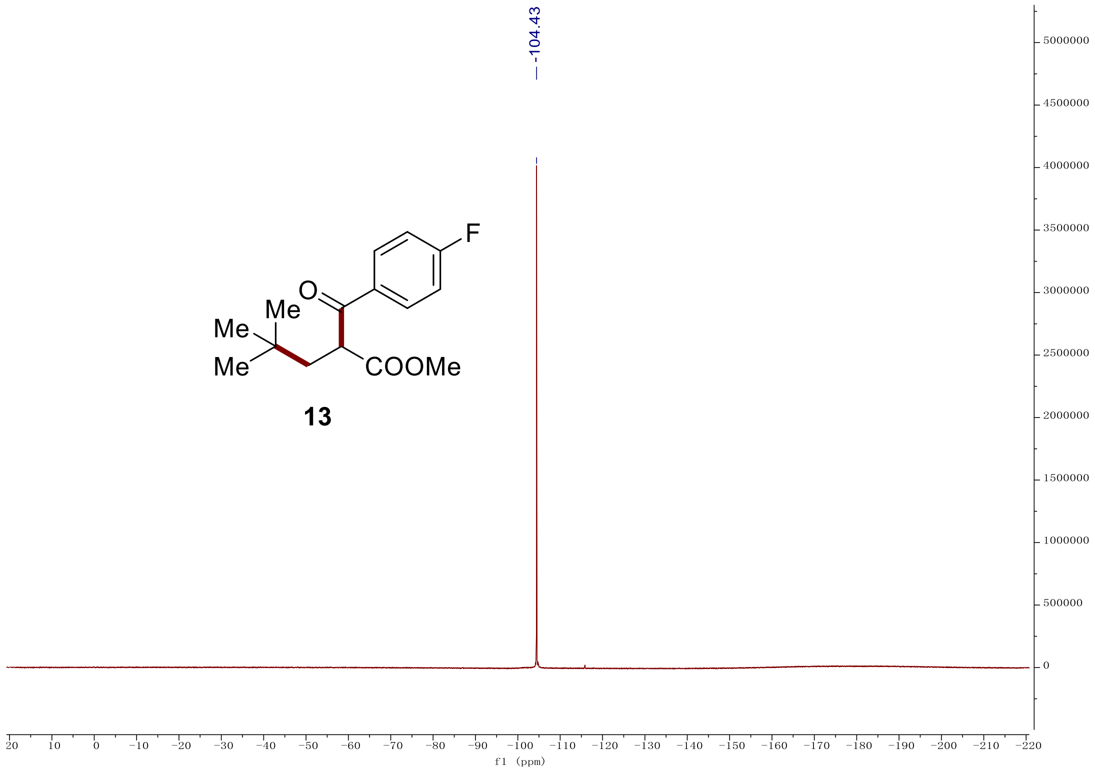

Figure S22. ^{19}F NMR of 13

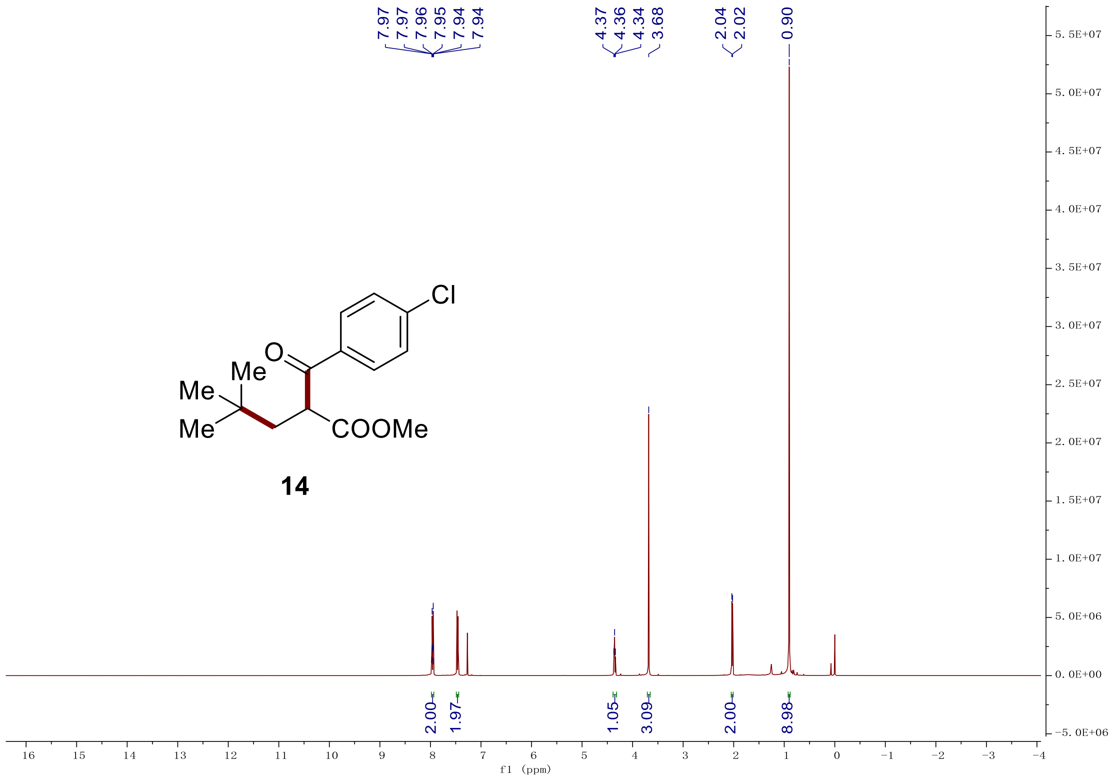

Figure S23. ¹H NMR of 14

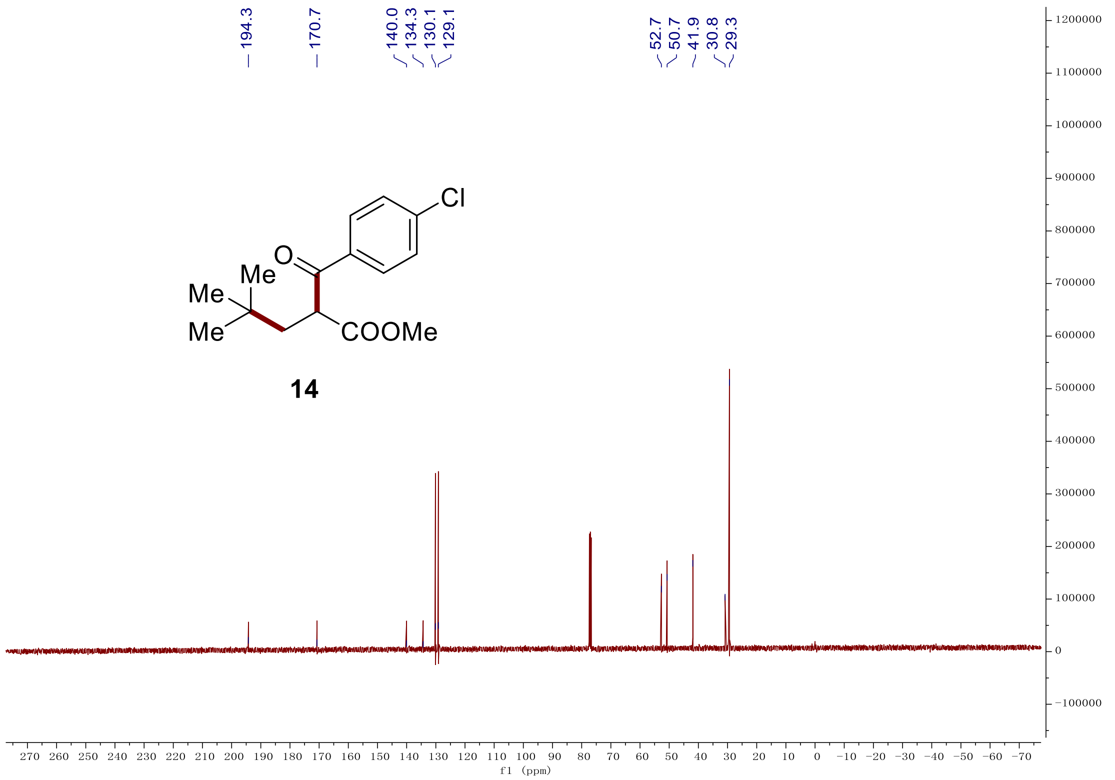

Figure S24. ¹³C NMR of 14

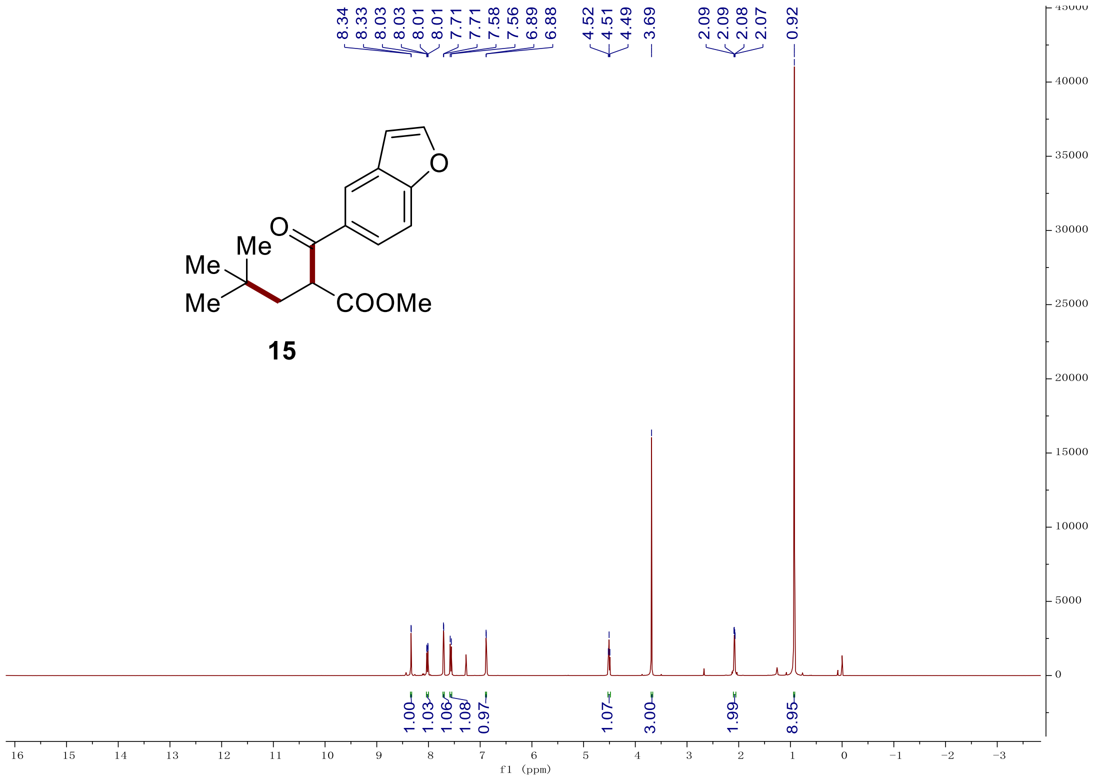

Figure S25. ^{1}**H NMR of 15**

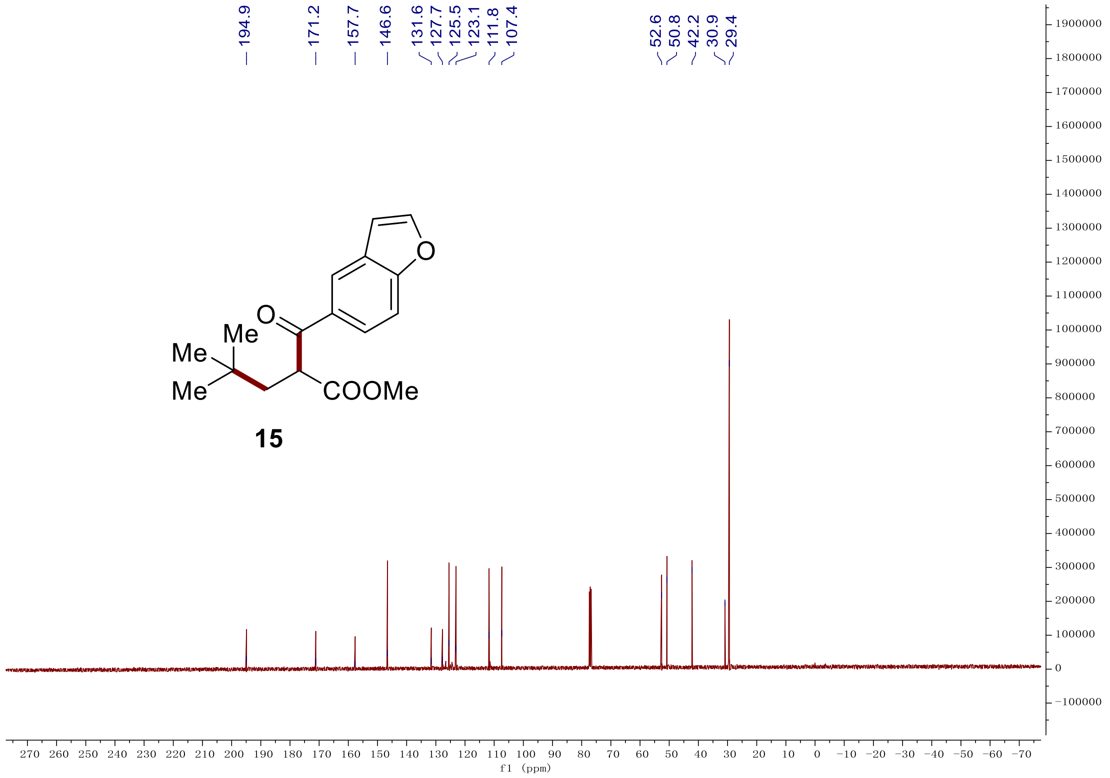

Figure S26. ^{13}**C NMR of 15**

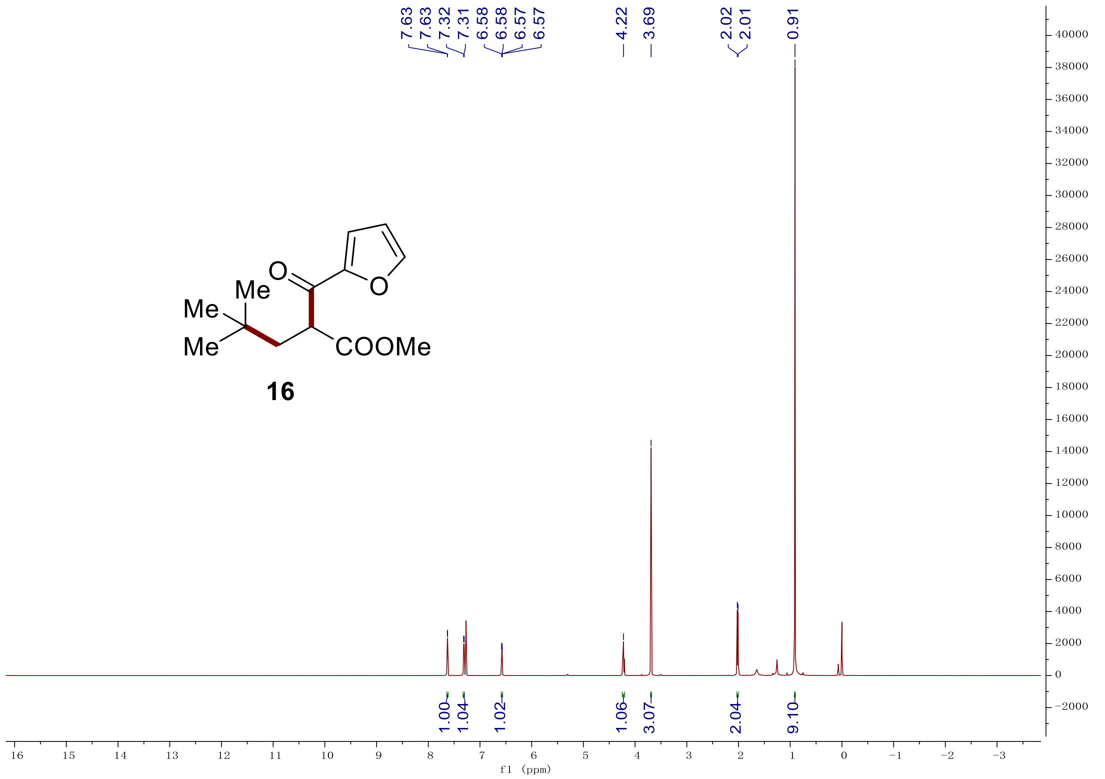

Figure S27. ^{1}H NMR of 16

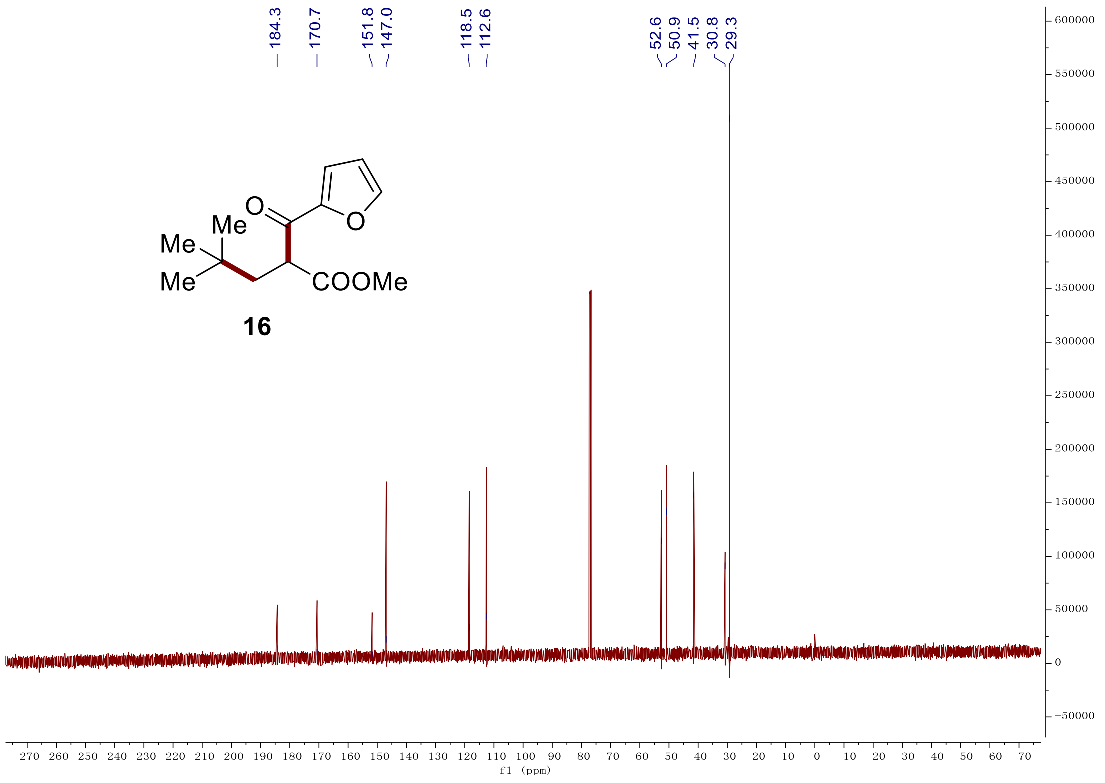

Figure S28. ^{13}C NMR of 16

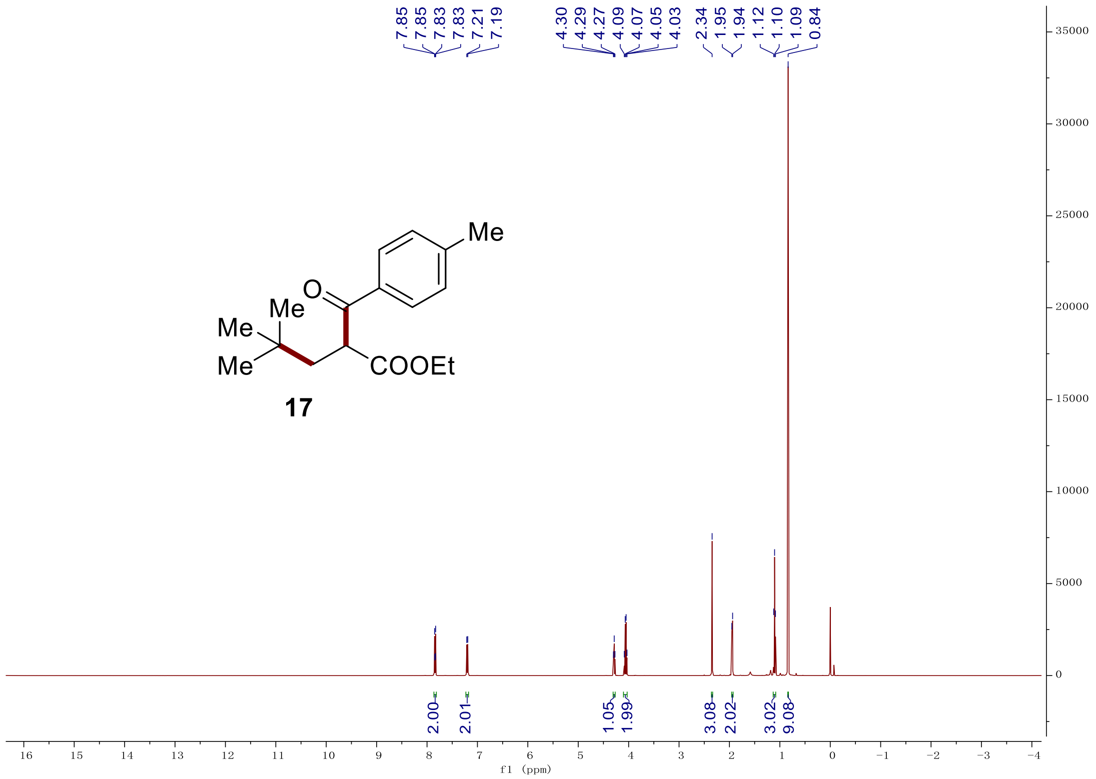

Figure S29. ^{1}H NMR of 17

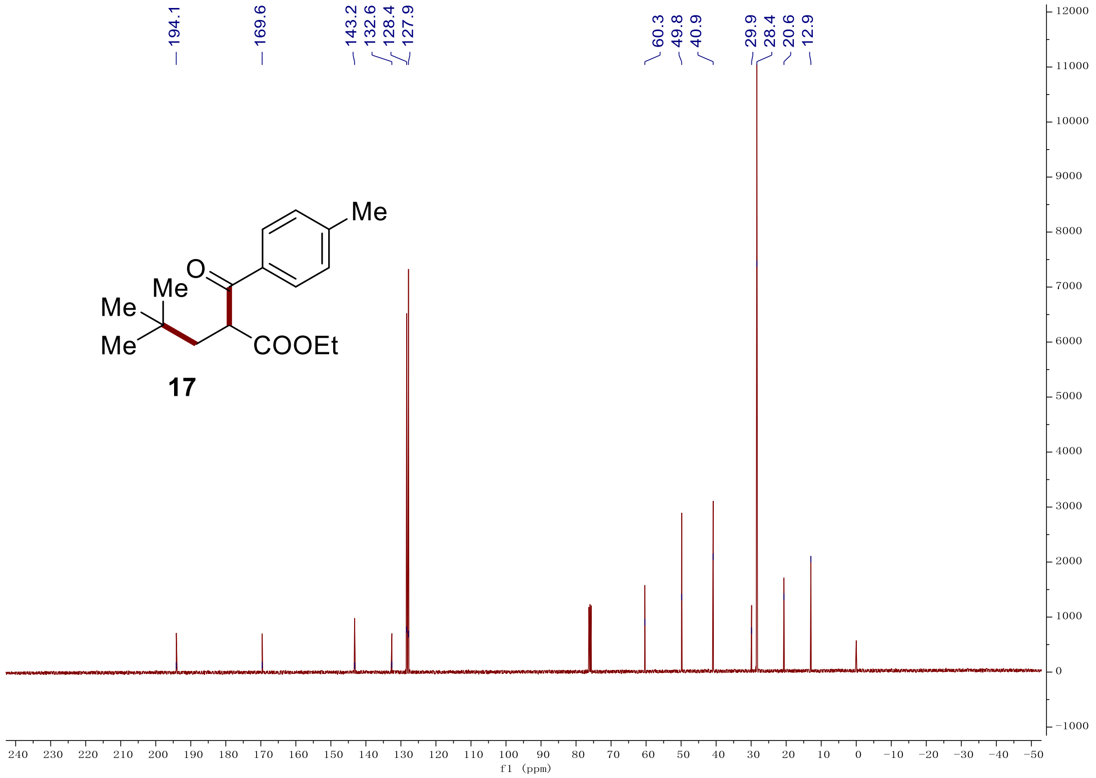

Figure S30. ^{13}C NMR of 17

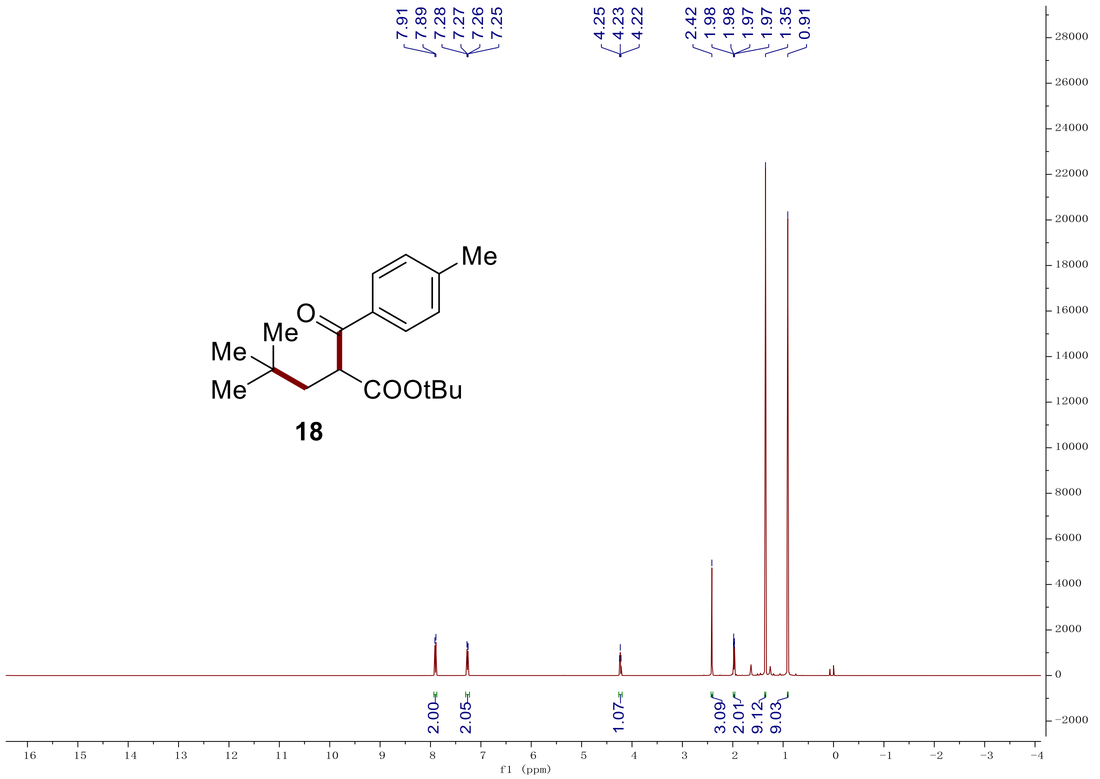

Figure S31. ^{1}H NMR of 18

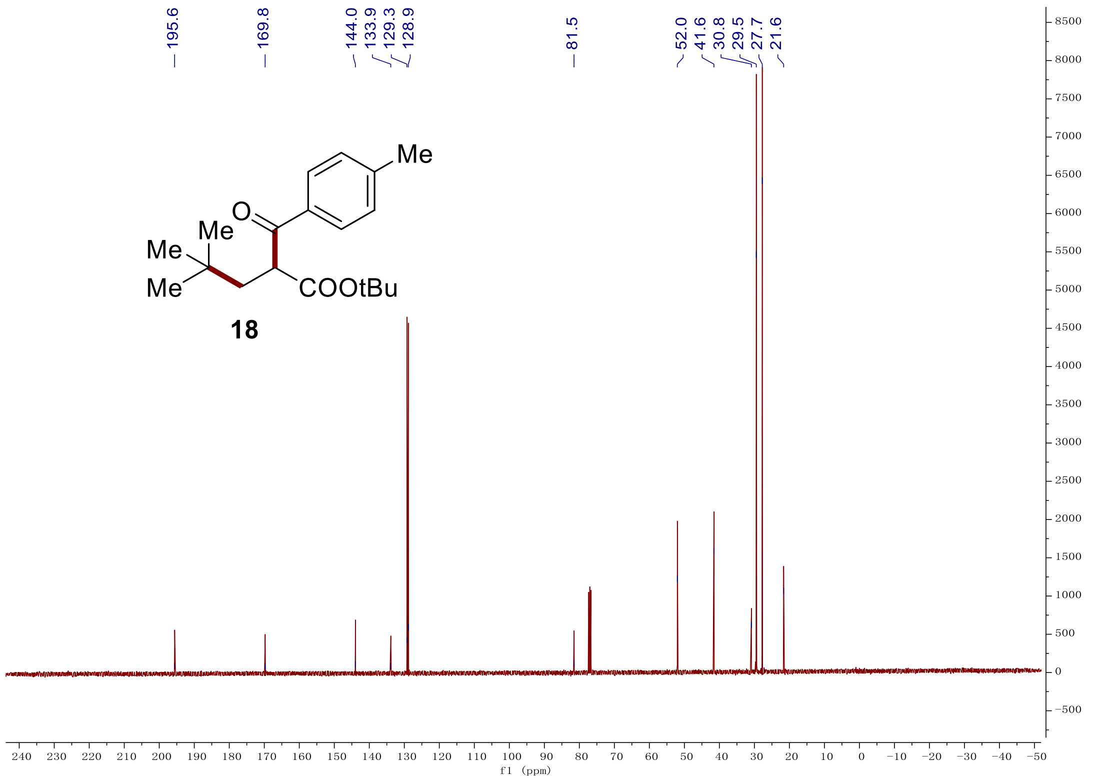

Figure S32. ^{13}C NMR of 18

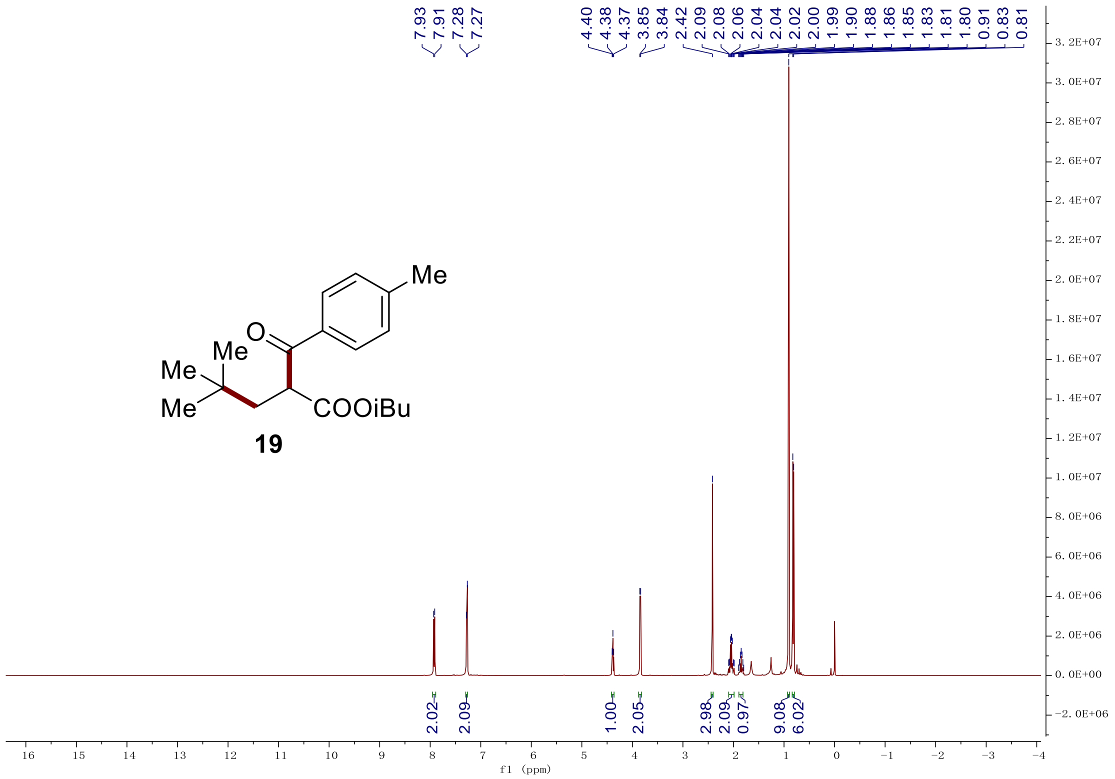

Figure S33. ^{1}H NMR of 19

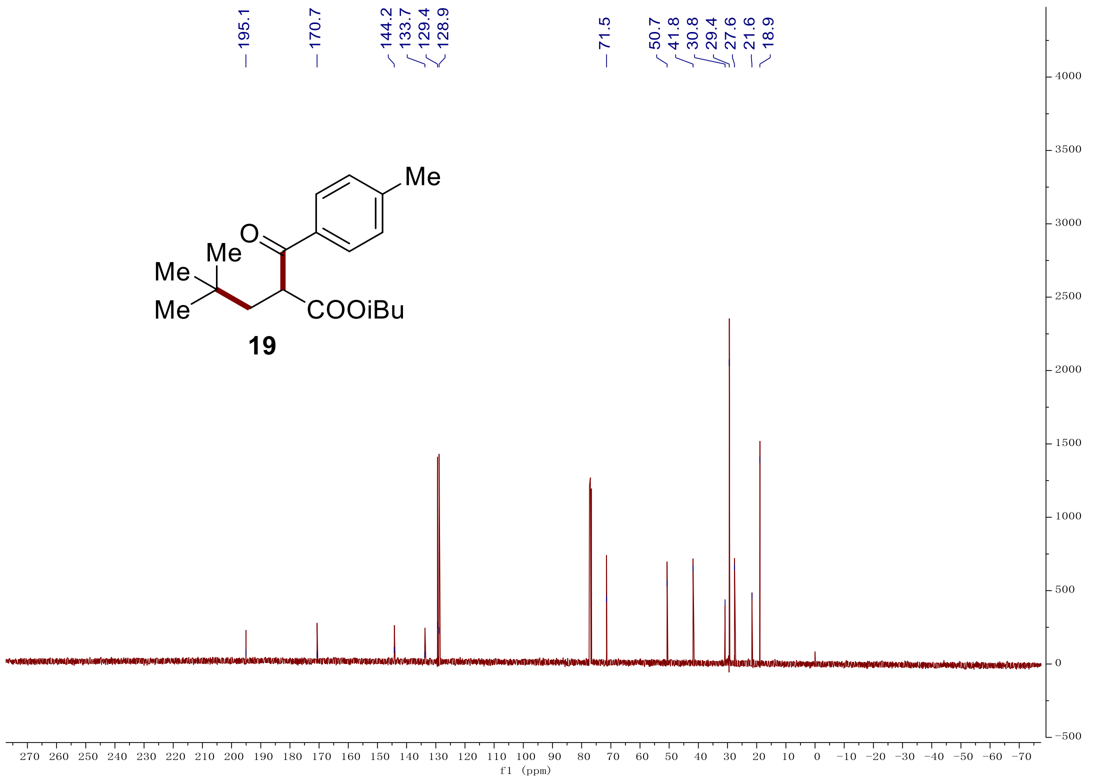

Figure S34. ^{13}C NMR of 19

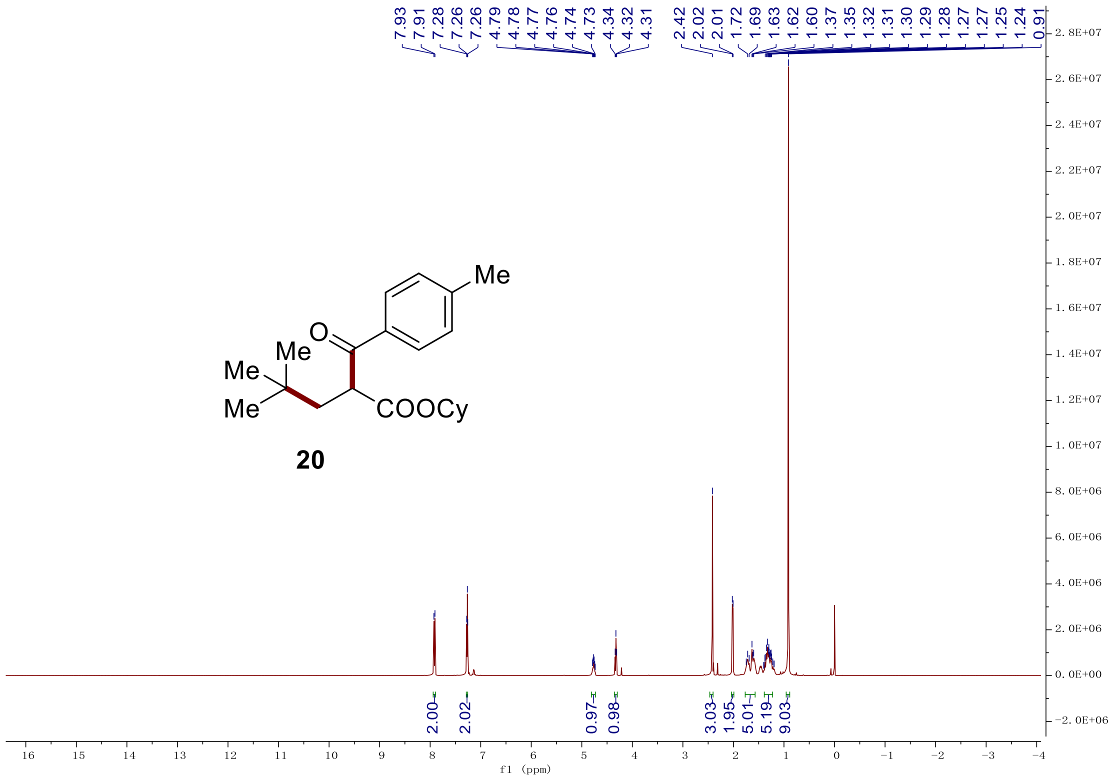

Figure S35. ^{1}H NMR of 20

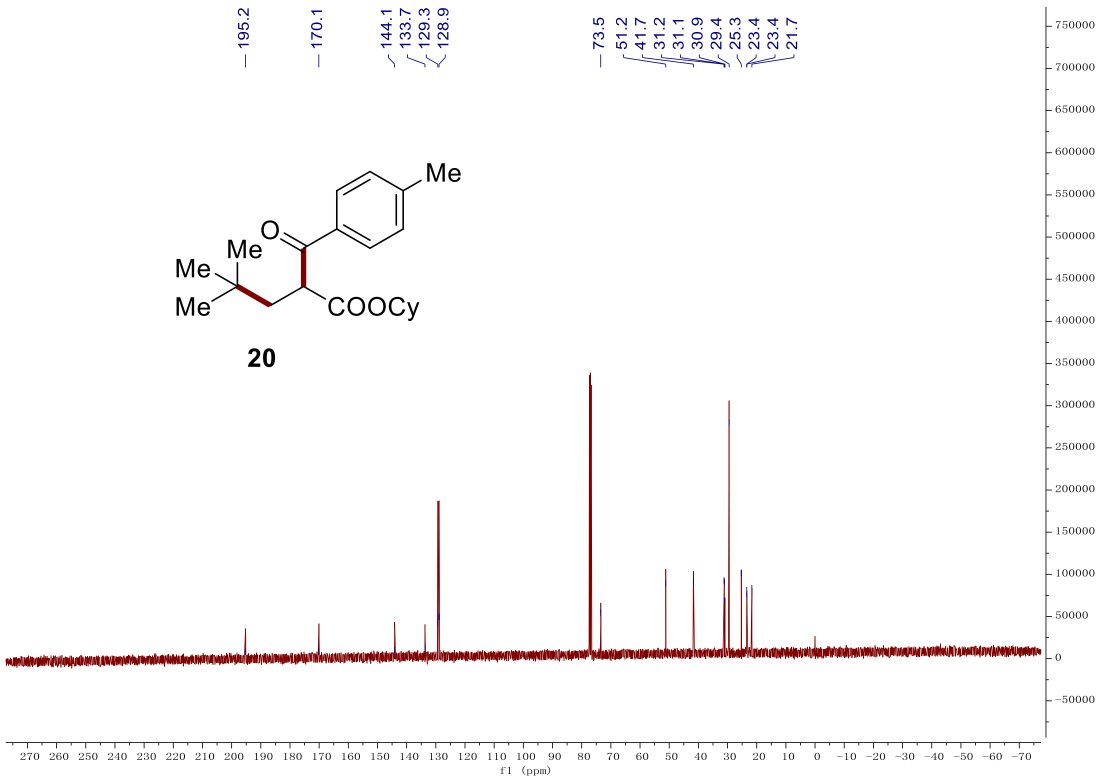

Figure S36. ^{13}C NMR of 20

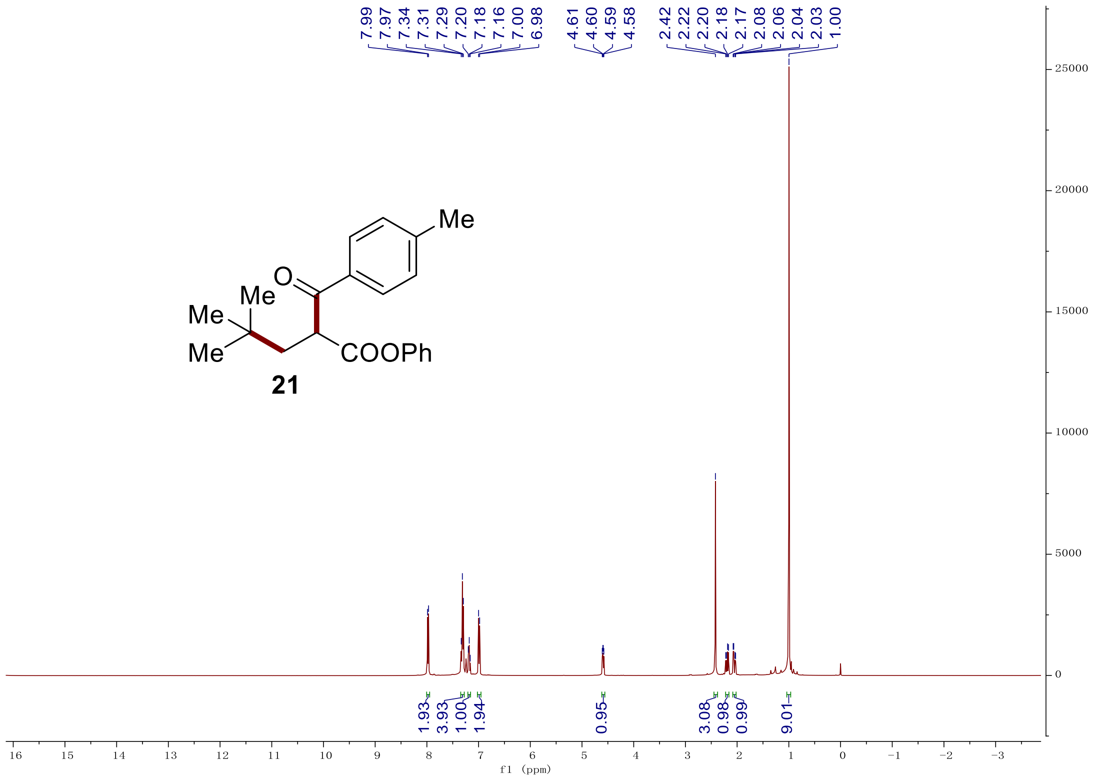

Figure S37. ^{1}H NMR of 21

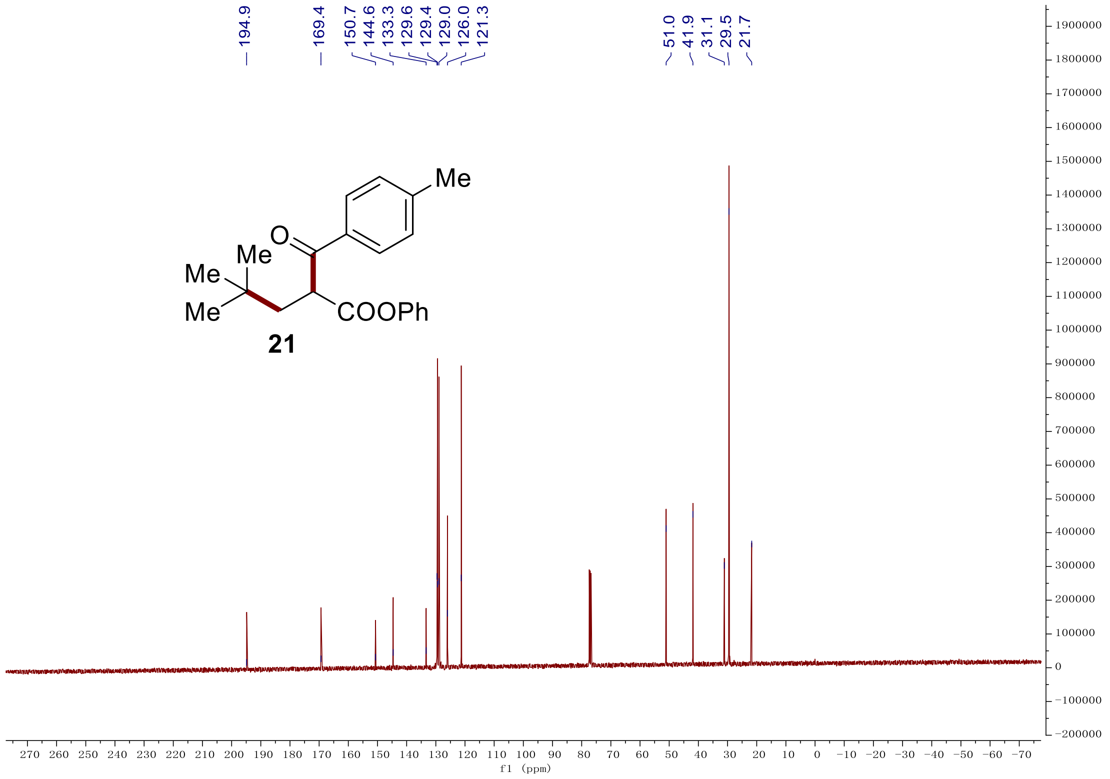

Figure S38. ^{13}C NMR of 21

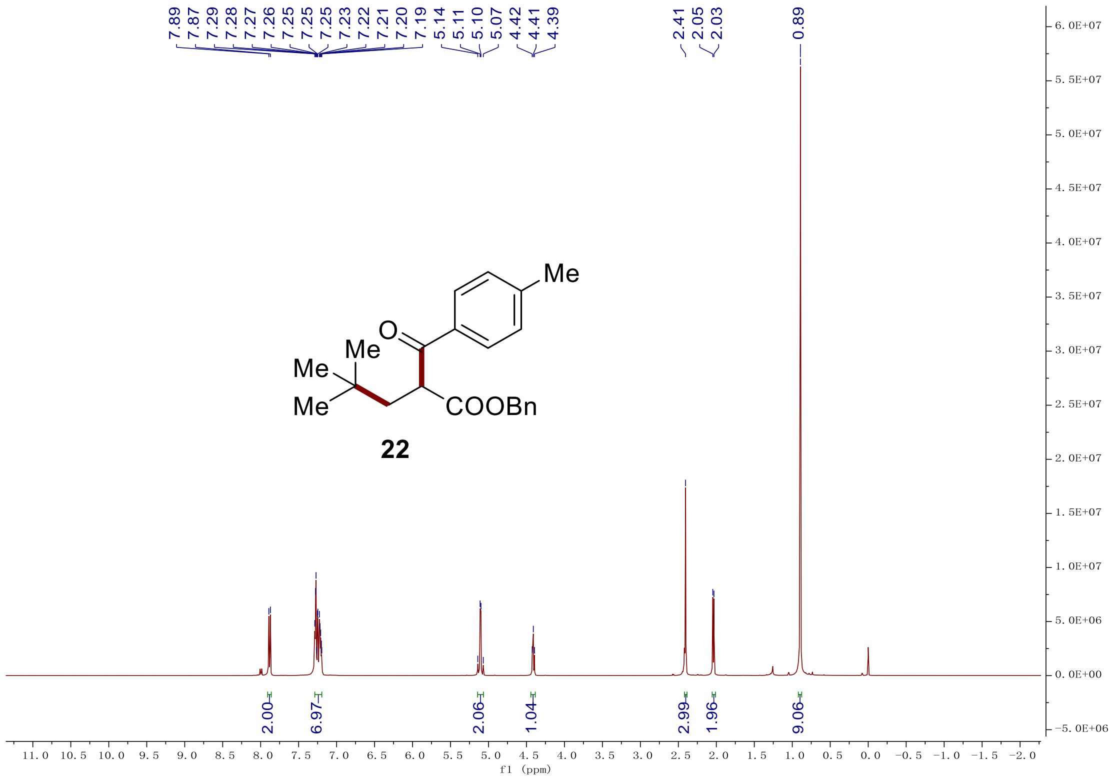

Figure S39. ^{1}**H NMR of 22**

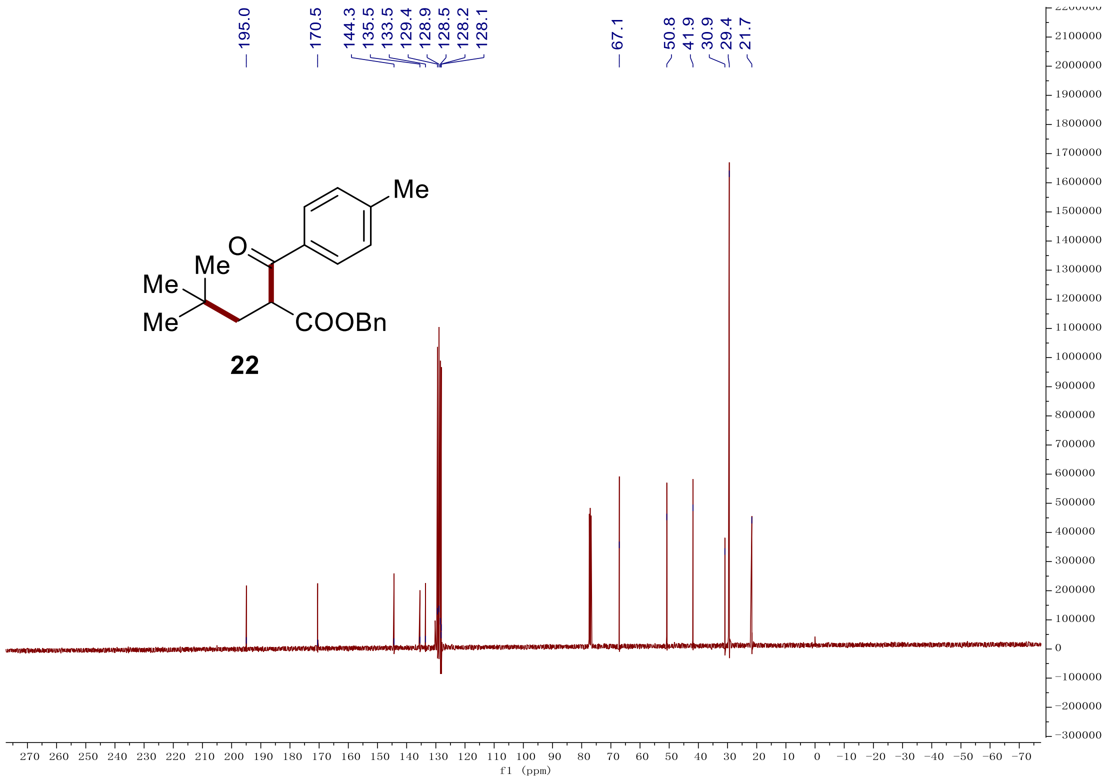

Figure S40. ^{13}**C NMR of 22**

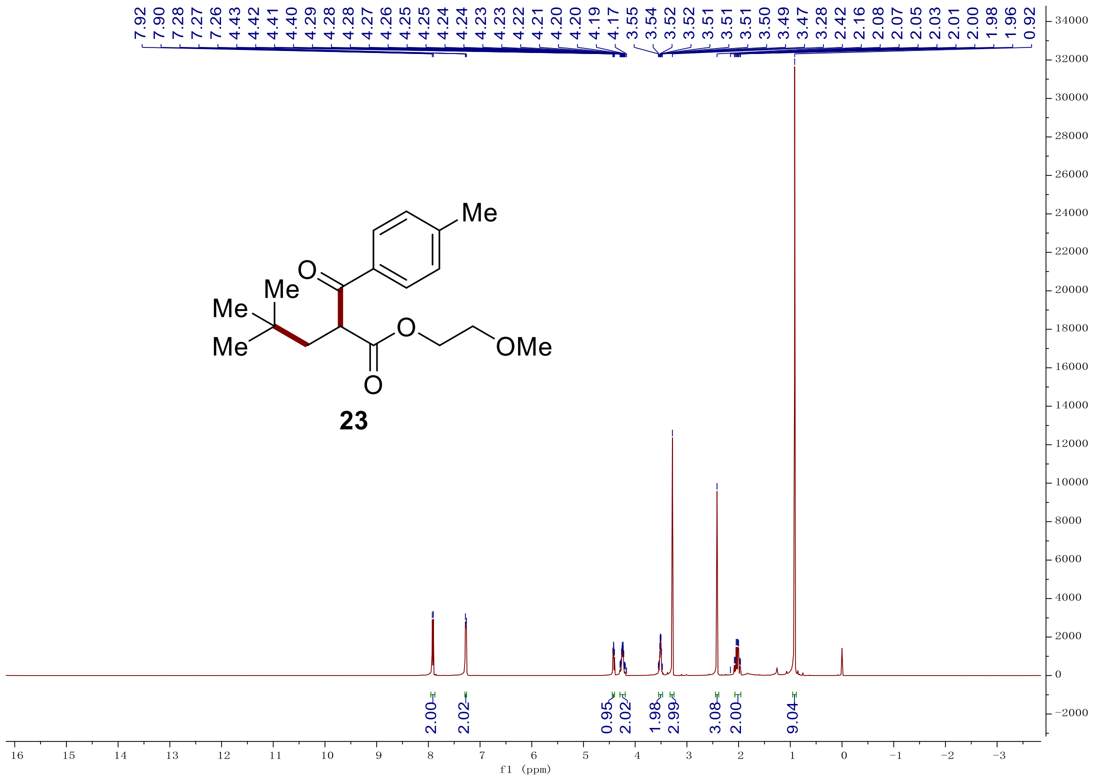

Figure S41. ^{1}**H NMR of 23**

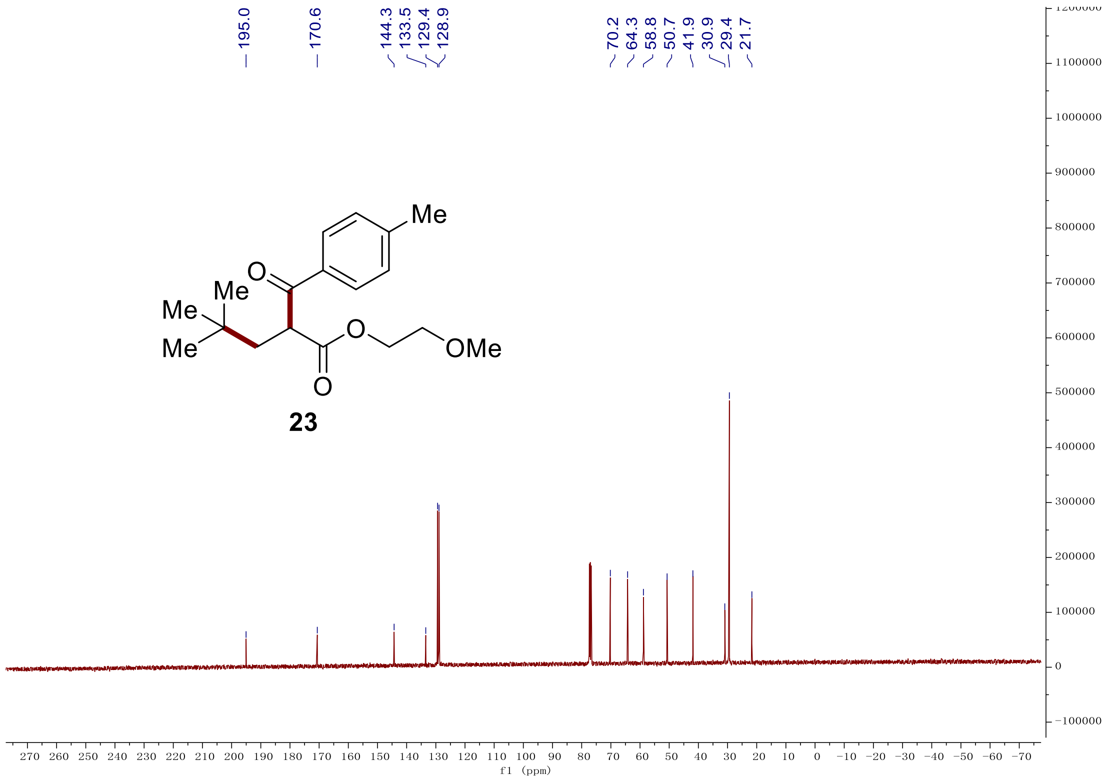

Figure S42. ^{13}**C NMR of 23**

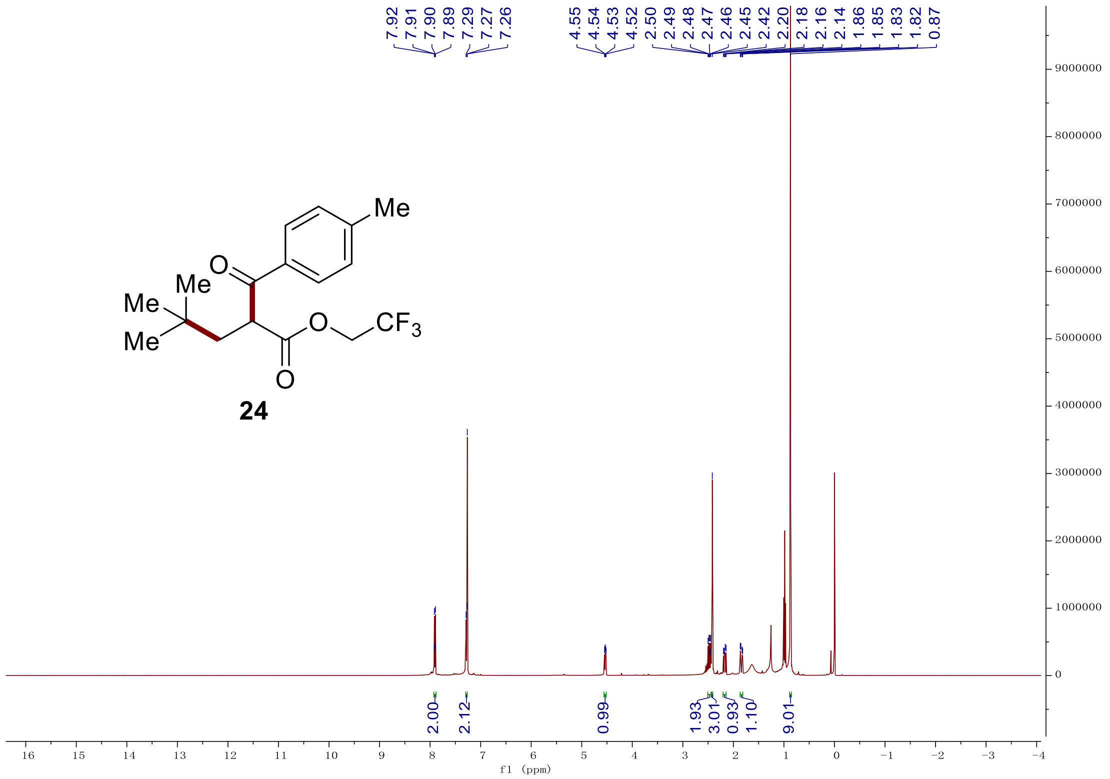

Figure S43. ^{1}H **NMR of 24**

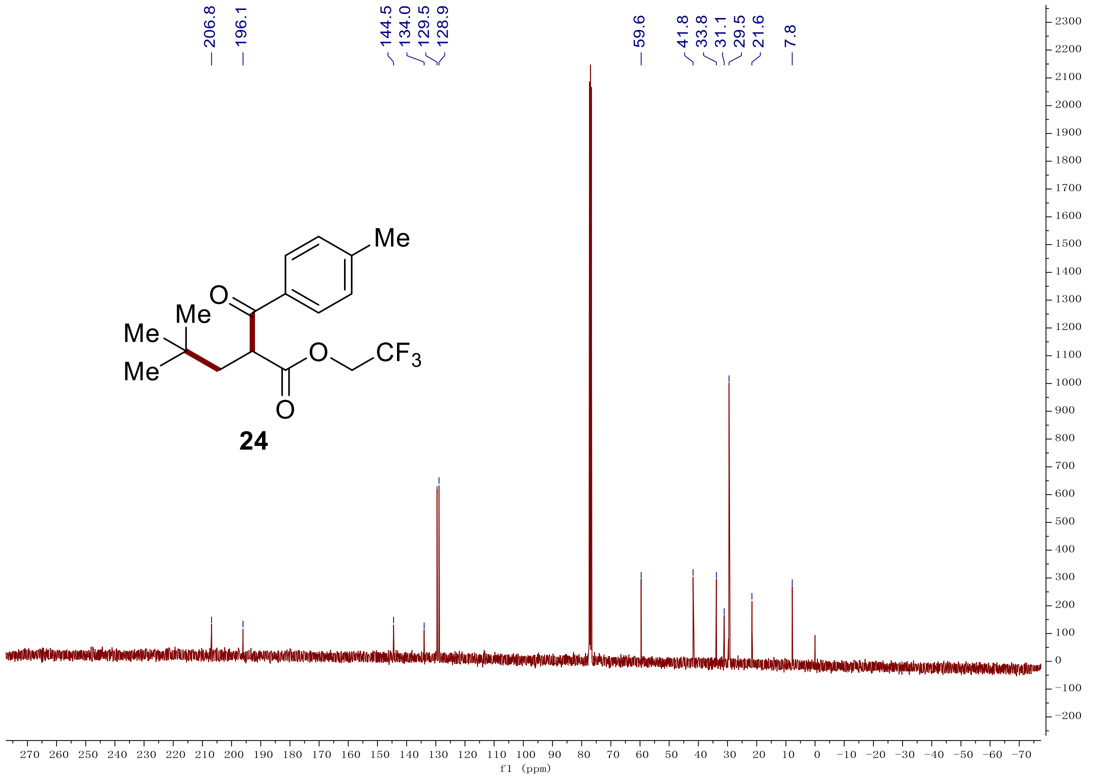

Figure S44. ^{13}C **NMR of 24**

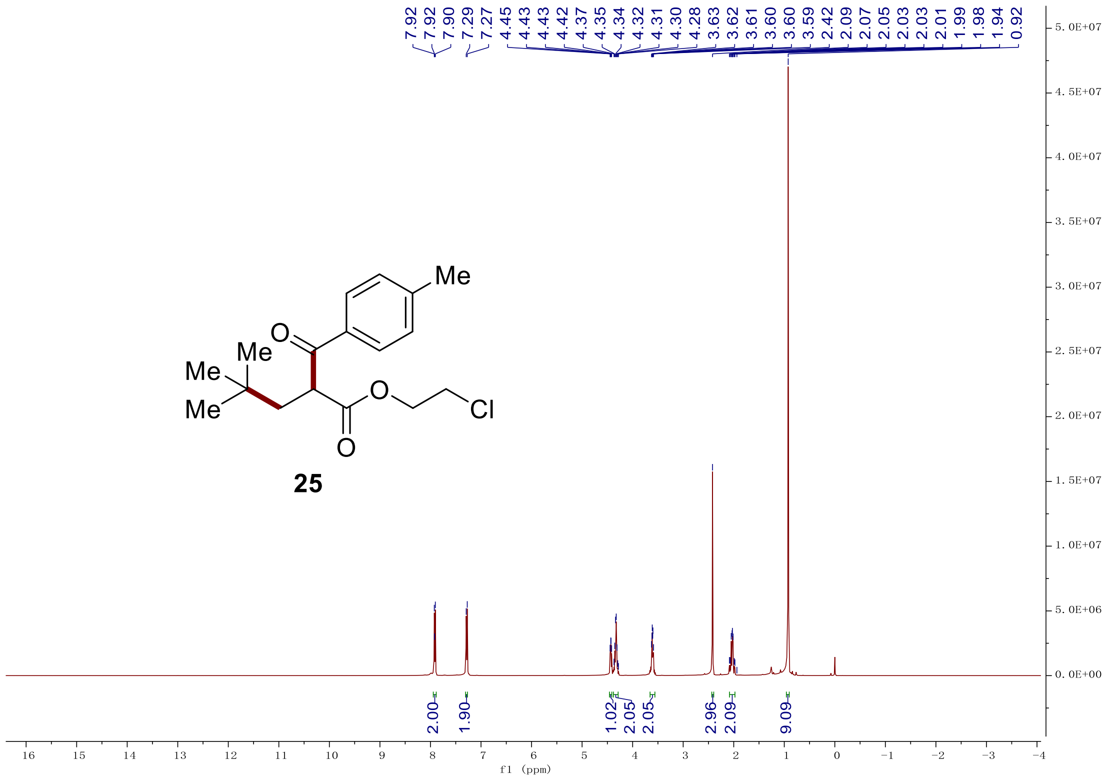

Figure S45. ^{1}H NMR of 25

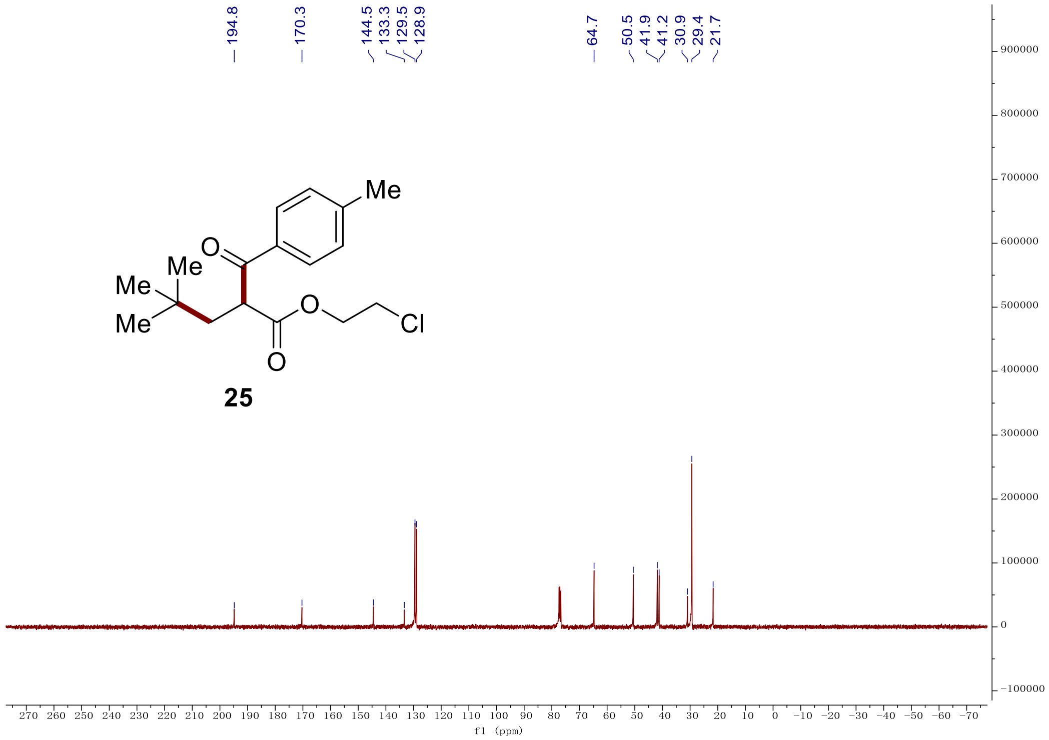

Figure S46. ^{13}C NMR of 25

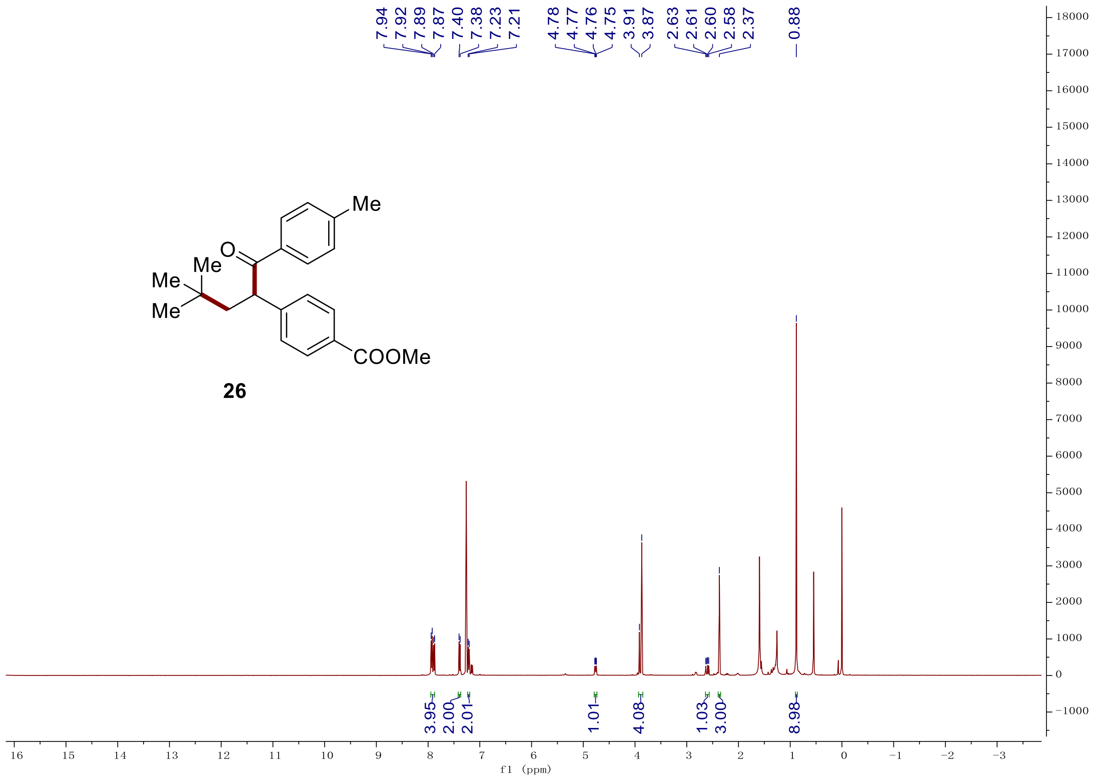

Figure S47. ^{1}H NMR of 26

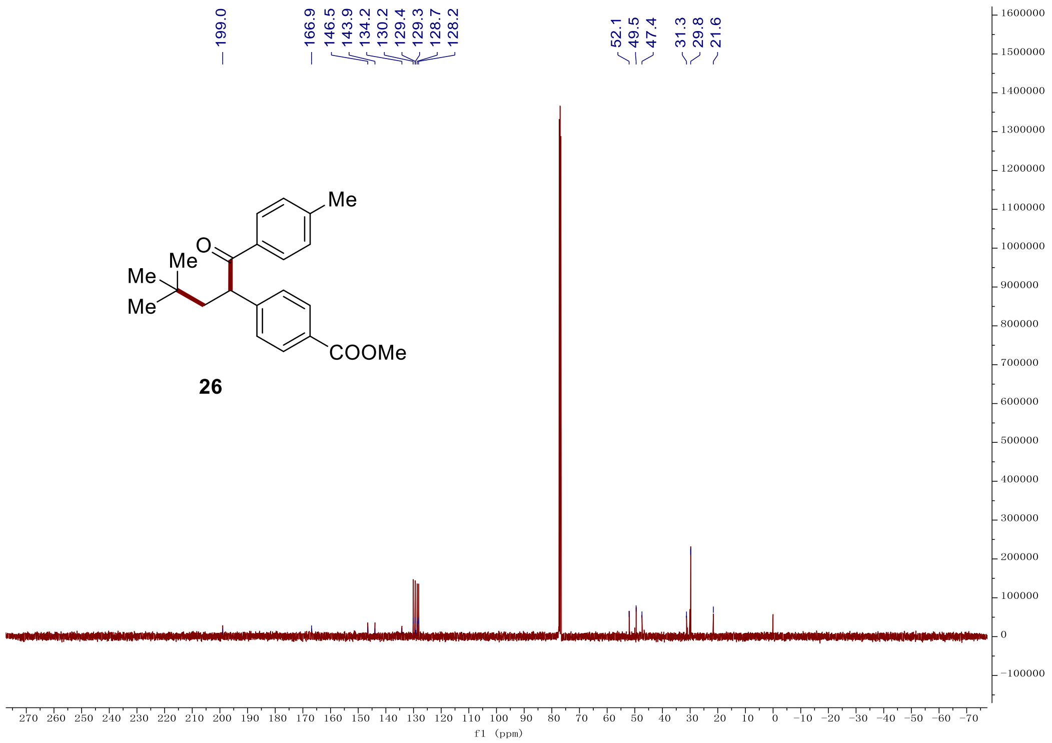

Figure S48. ^{13}C NMR of 26

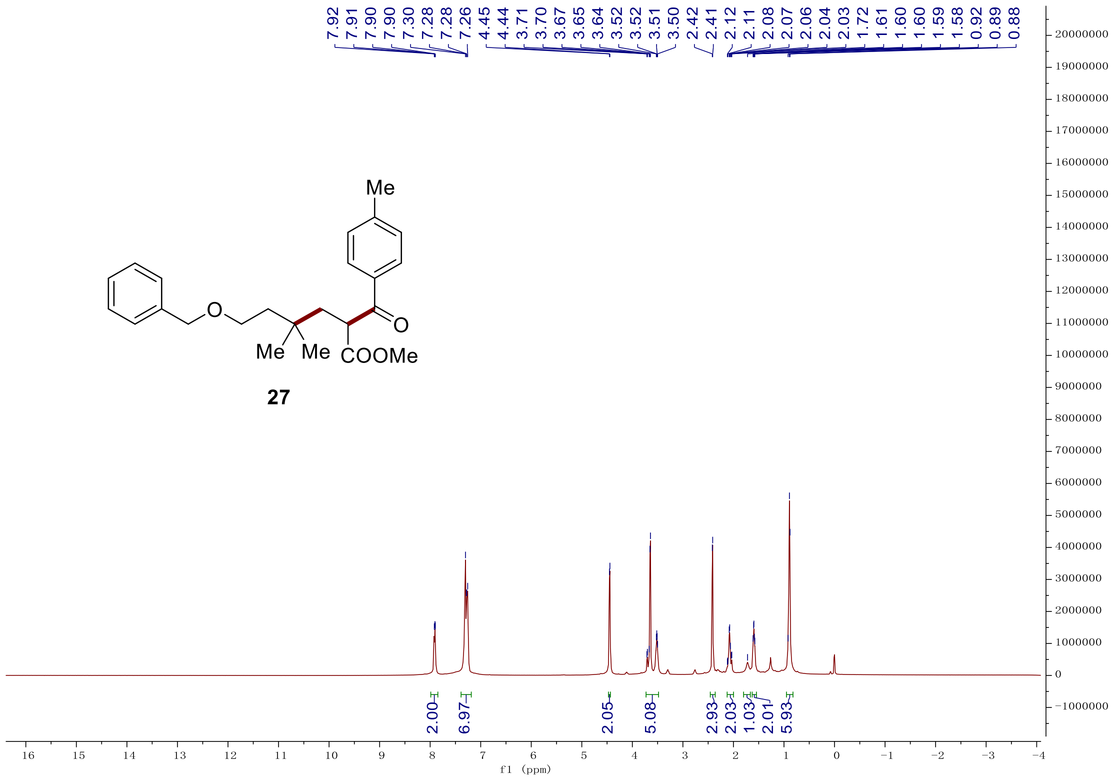

Figure S49. ^{1}H NMR of 27

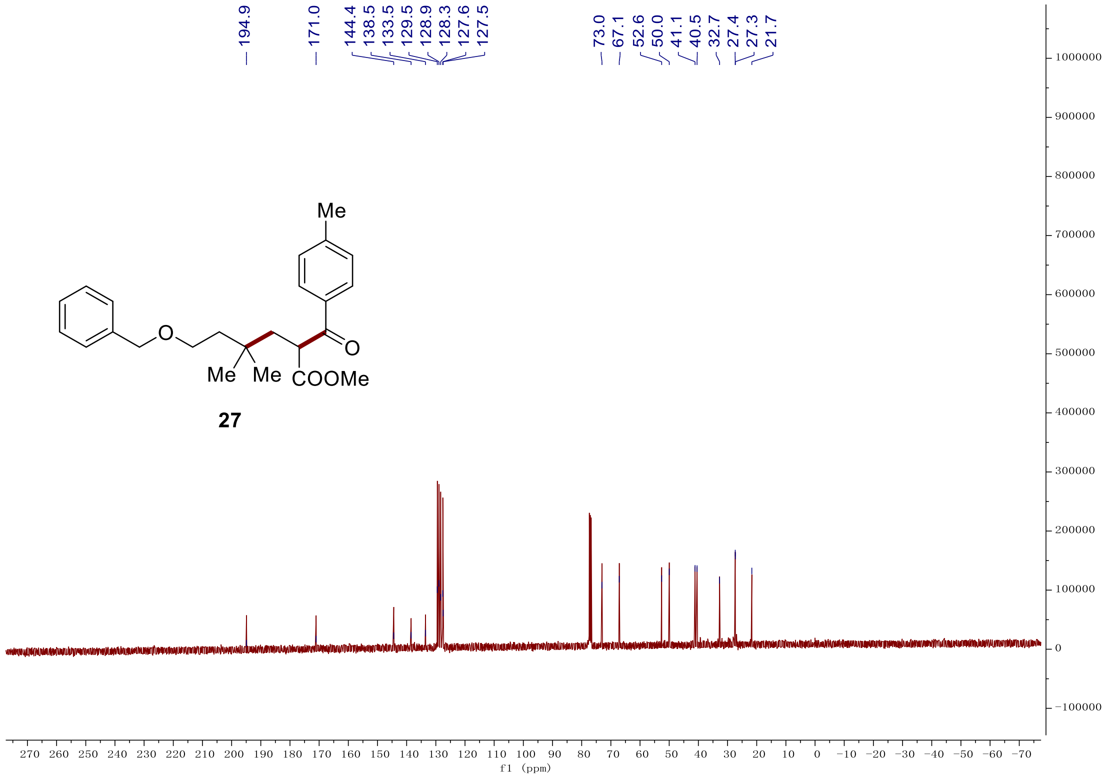

Figure S50. ^{13}C NMR of 27

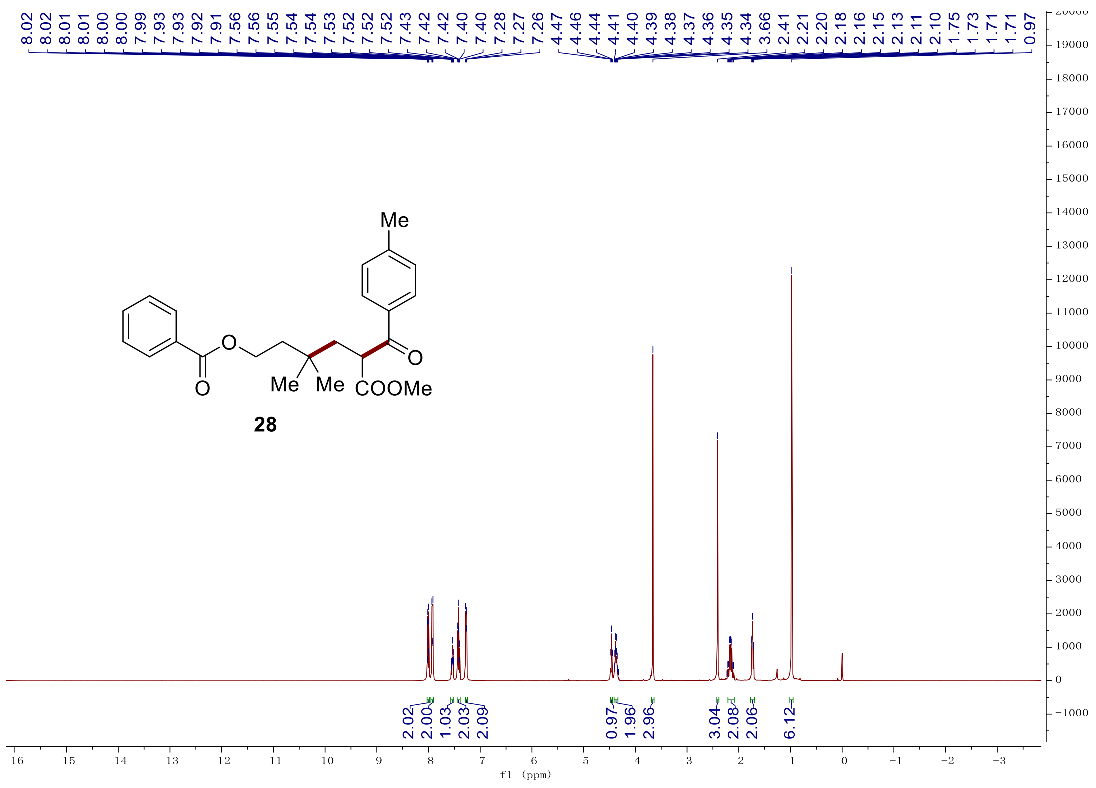

Figure S51. ^{1}H NMR of 28

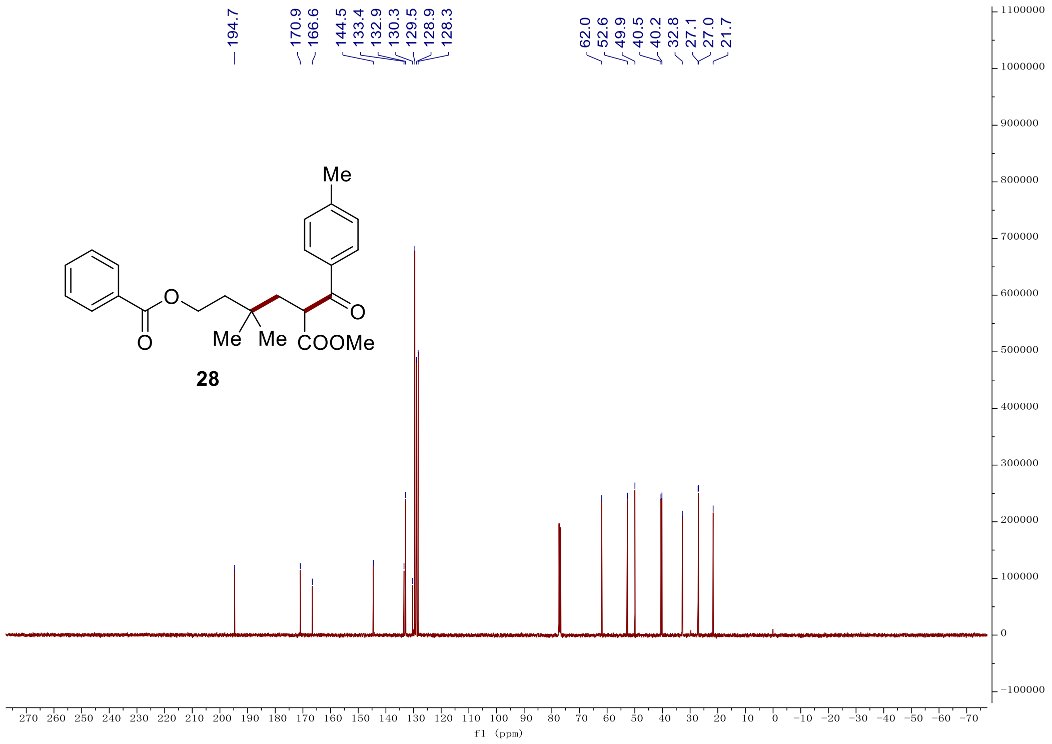

Figure S52. ^{13}C NMR of 28

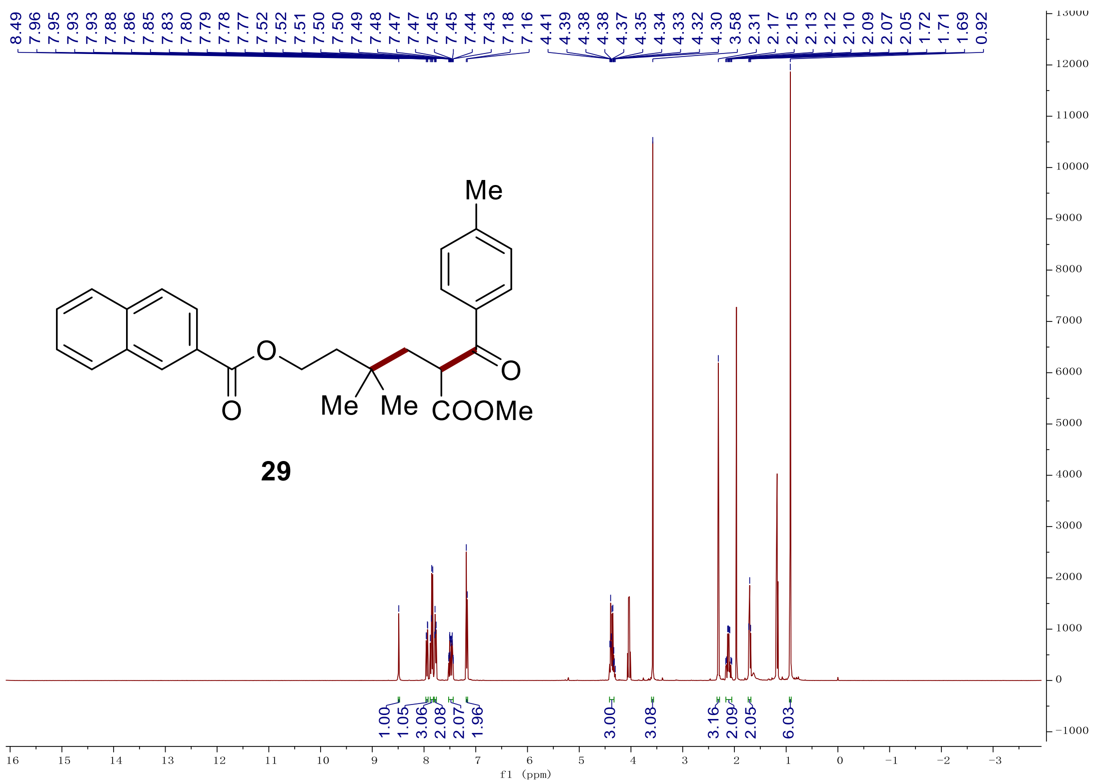

Figure S53. ^{1}H NMR of 29

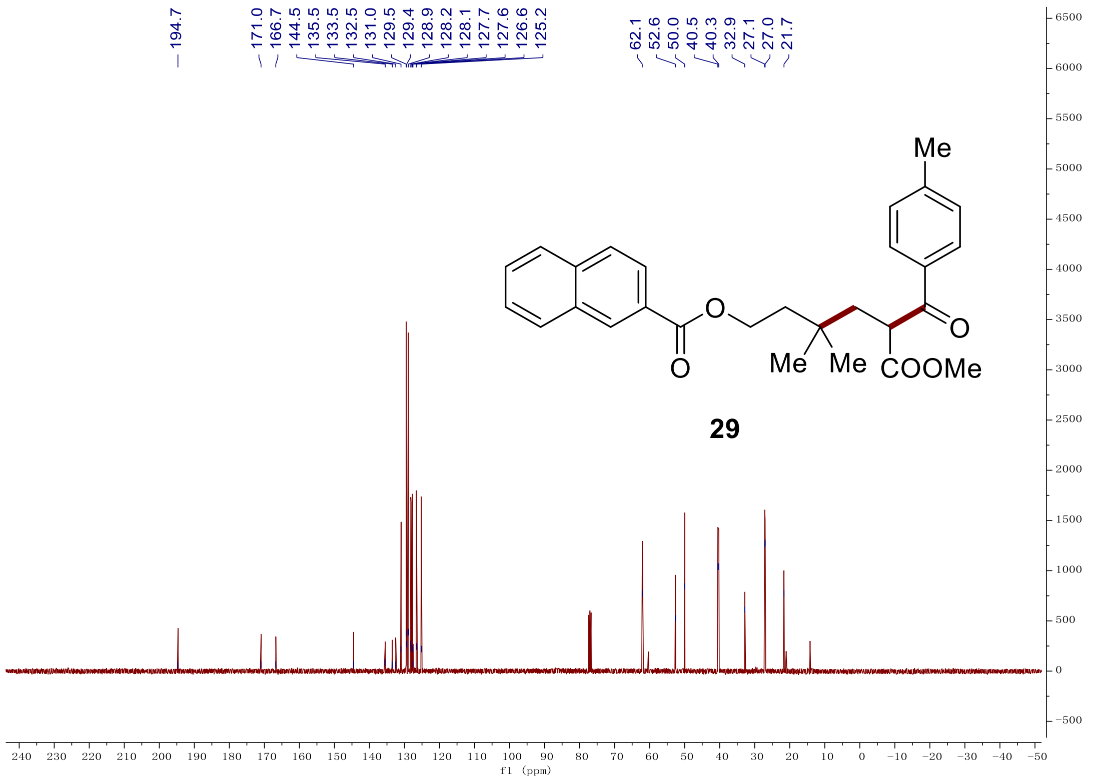

Figure S54. ^{13}C NMR of 29

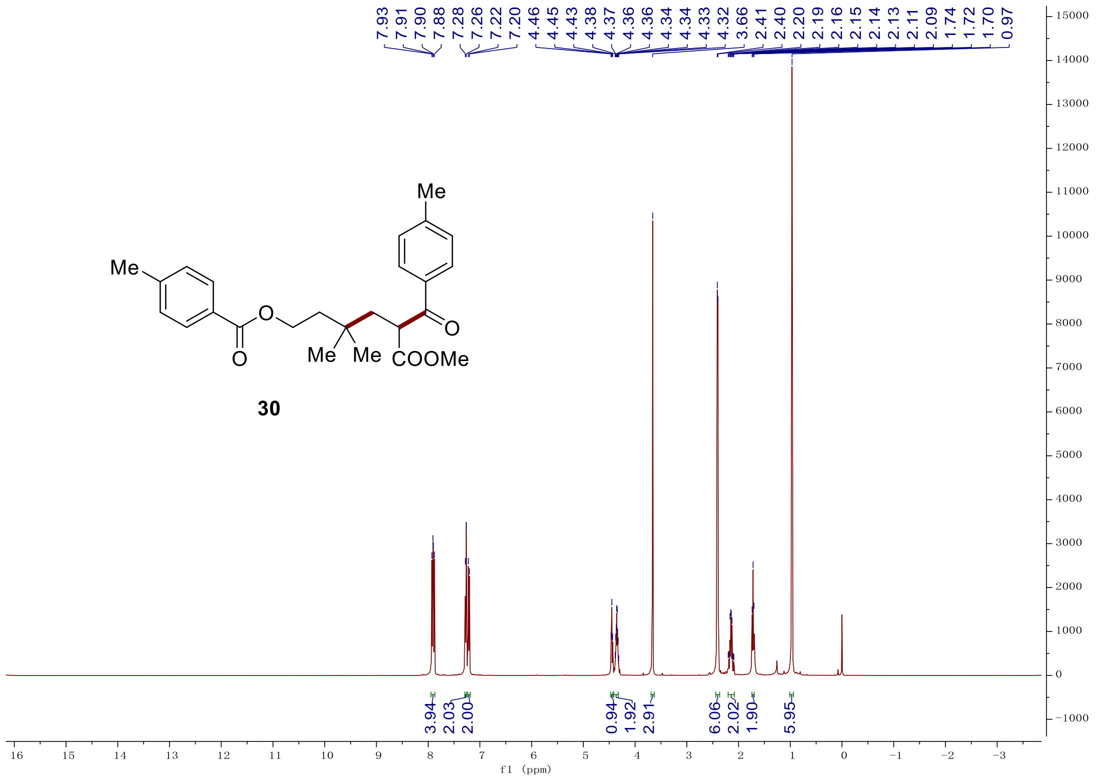

Figure S55. ^{1}H NMR of 30

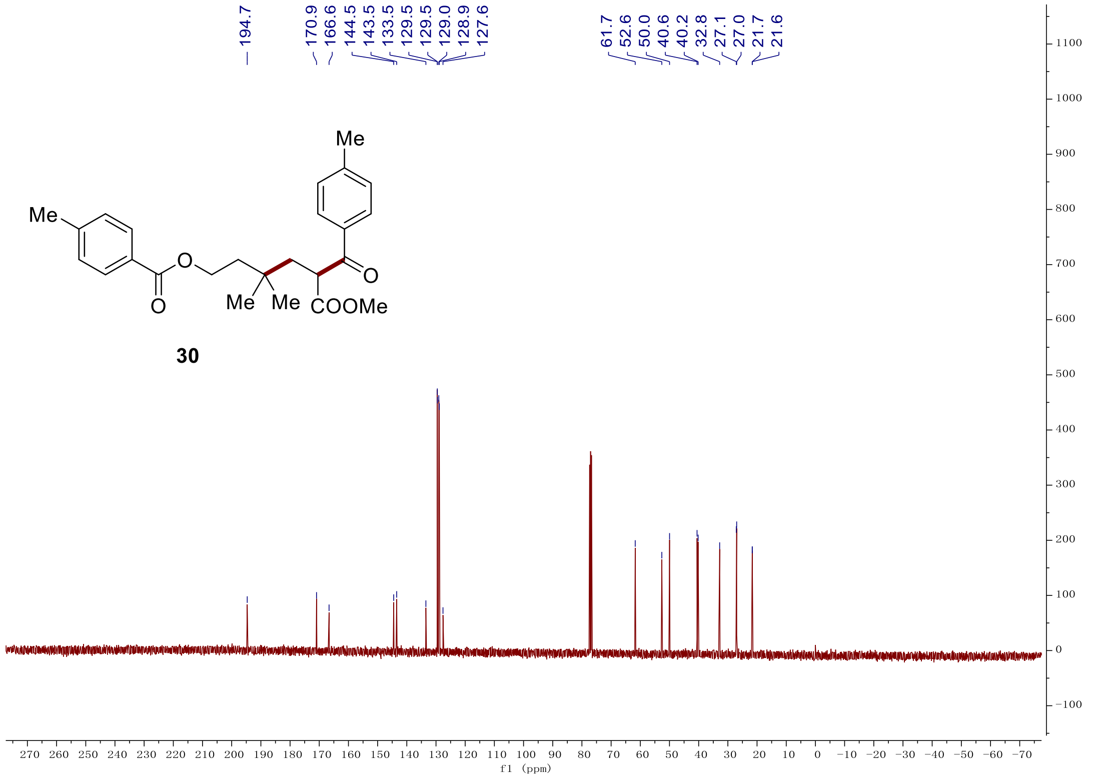

Figure S56. ^{13}C NMR of 30

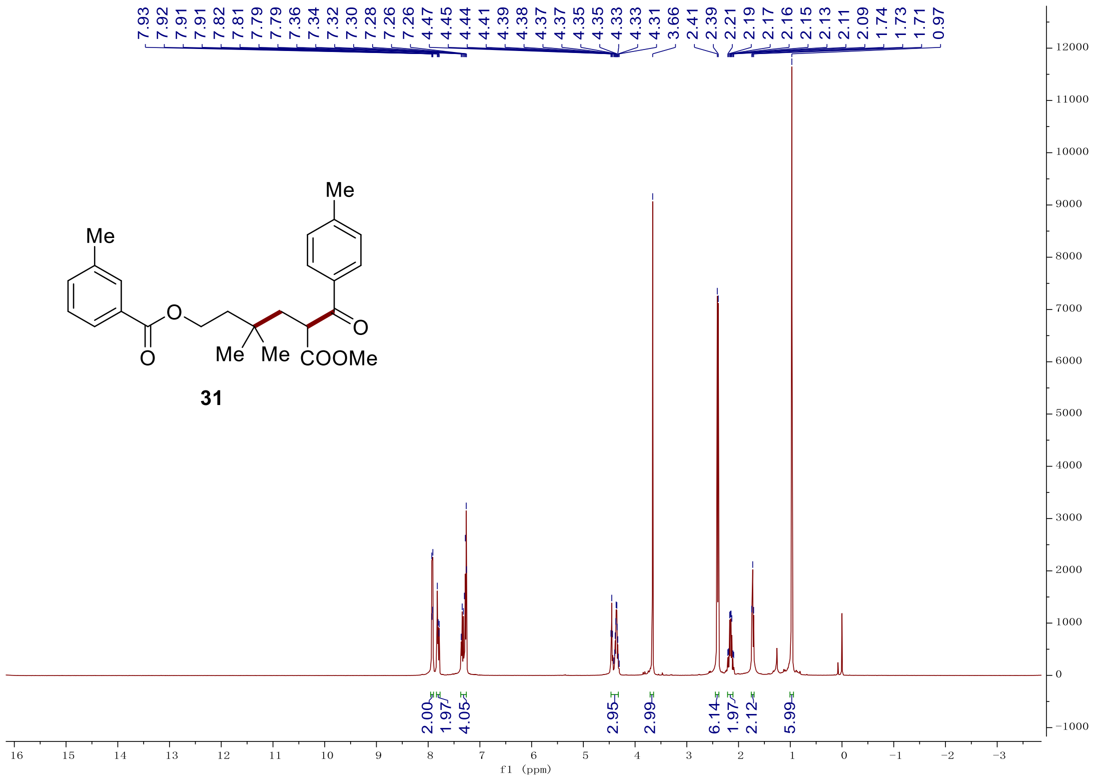

Figure S57. ^{1}H NMR of 31

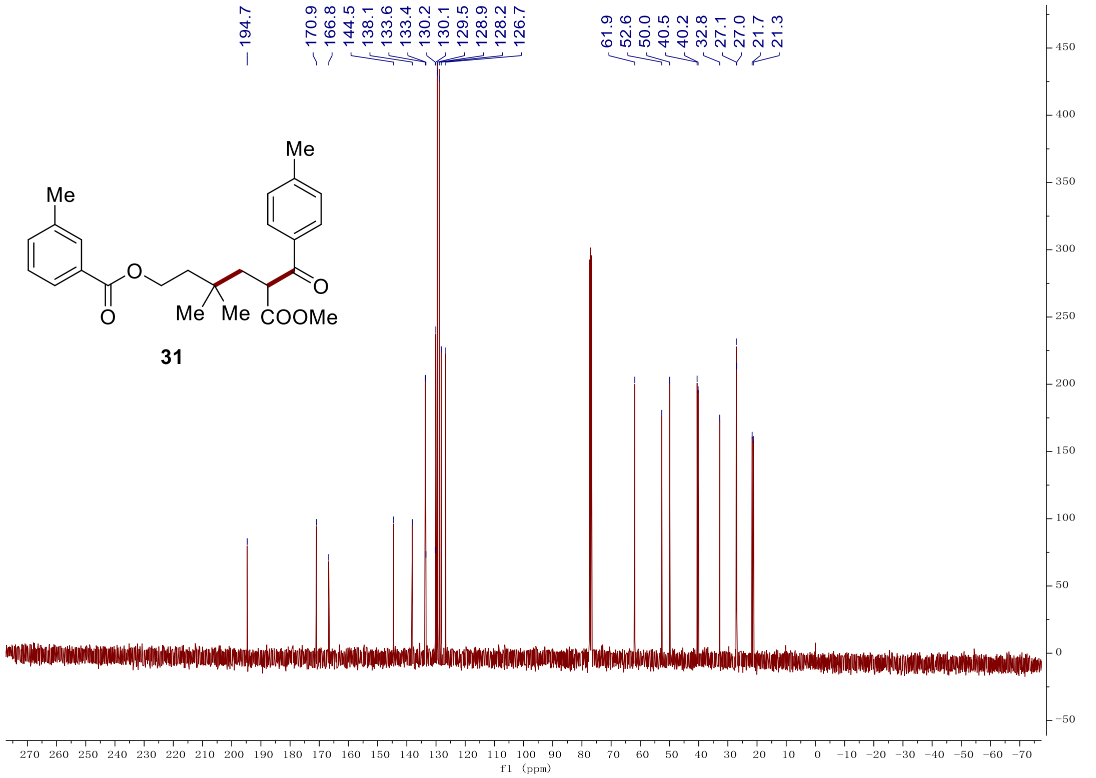

Figure S58. ^{13}C NMR of 31

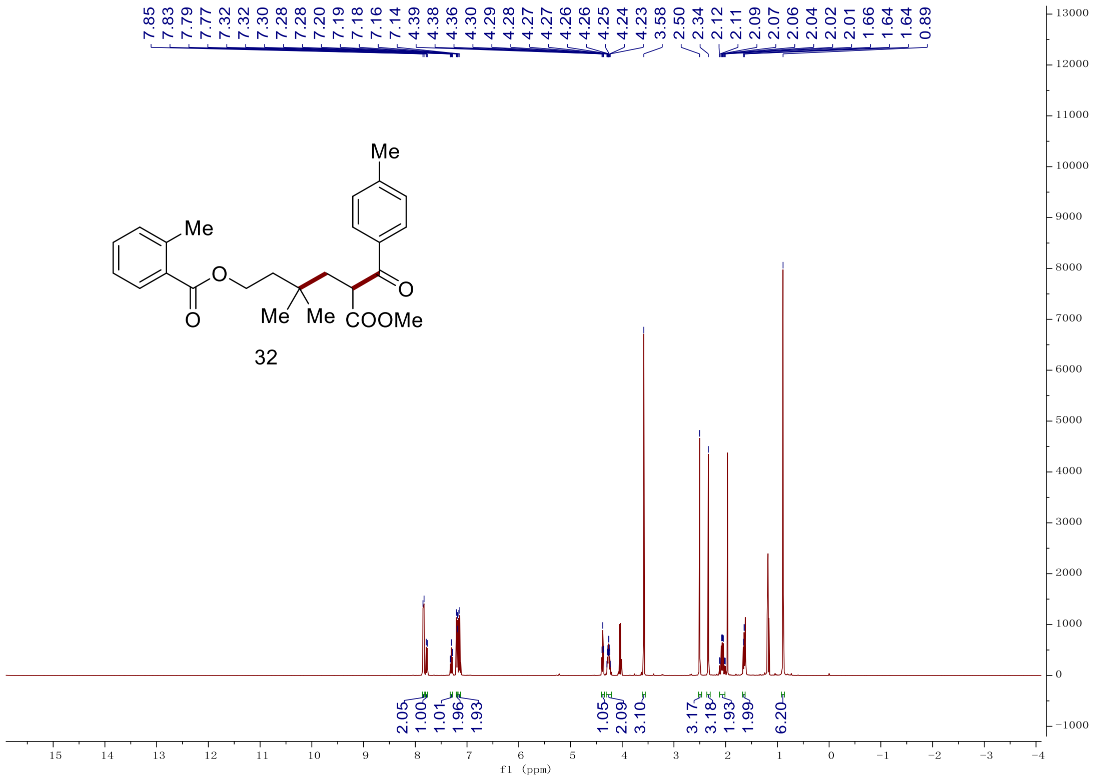

Figure S59. ^{1}H NMR of 32

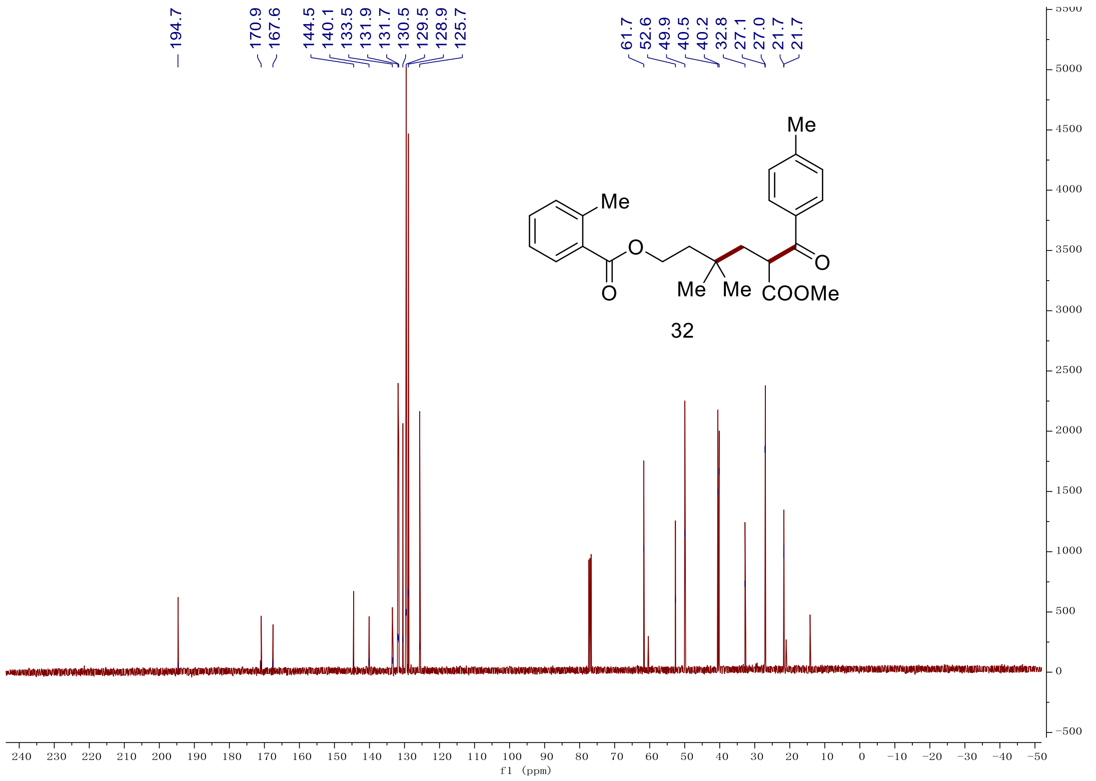

Figure S60. ^{13}C NMR of 32

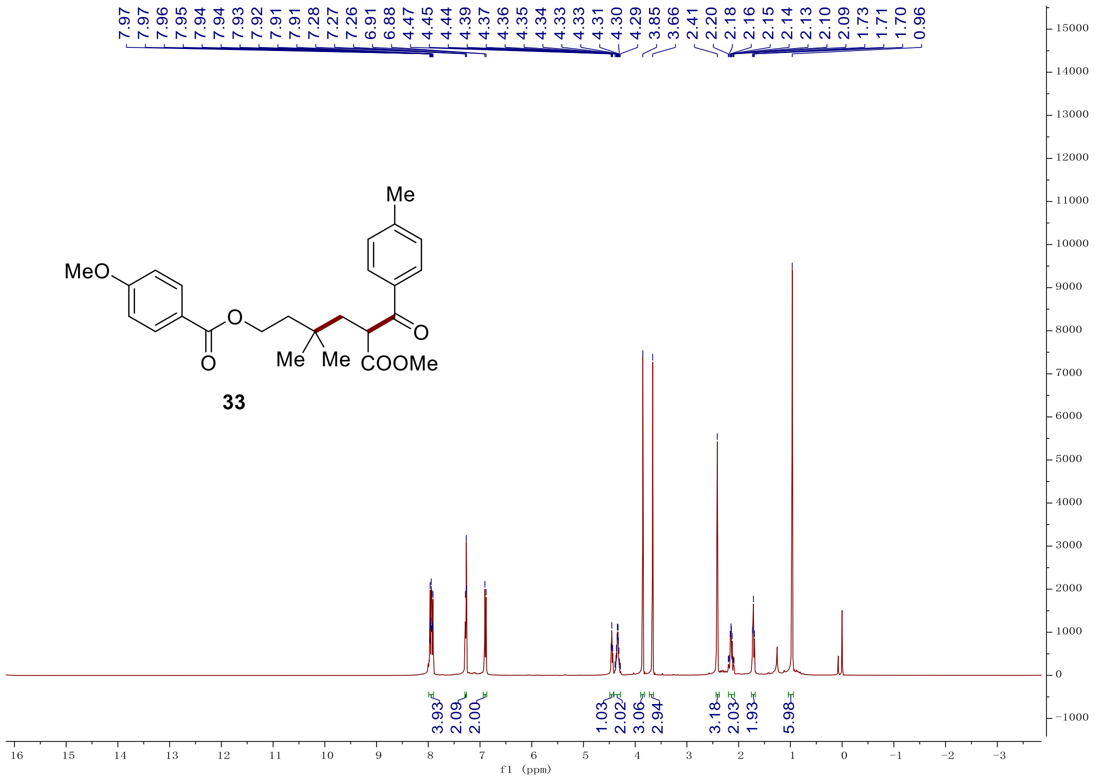

Figure S61. ^{1}H NMR of 33

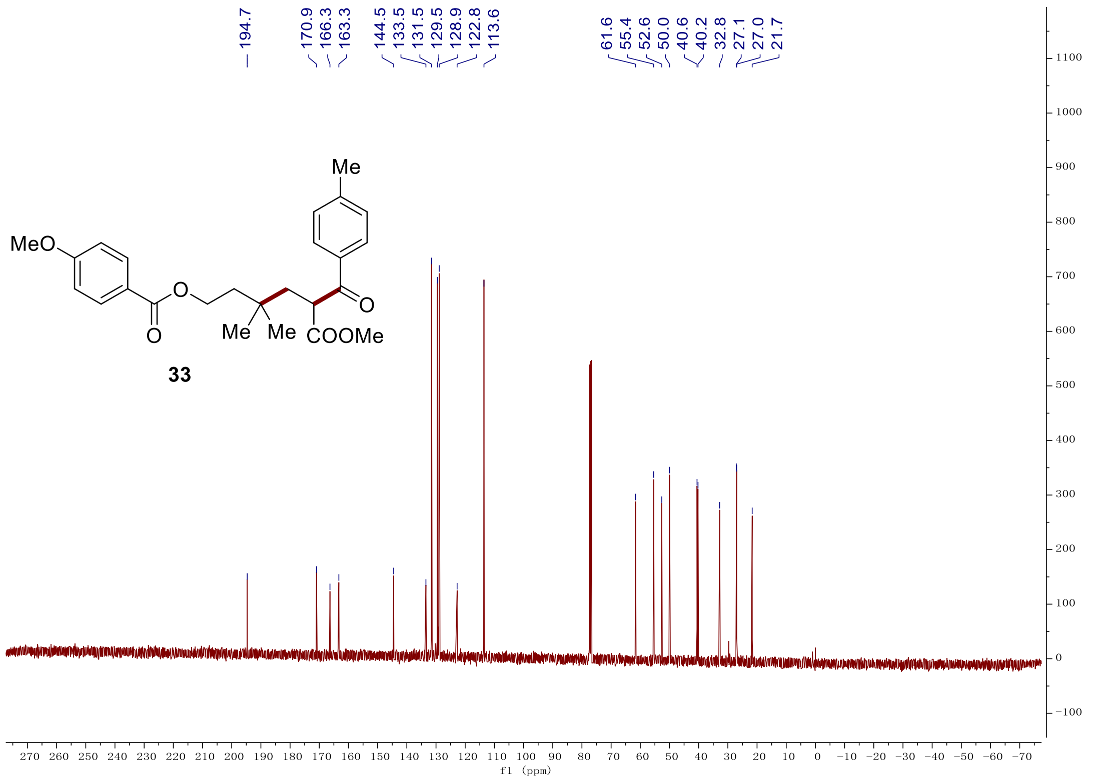

Figure S62. ^{13}C NMR of 33

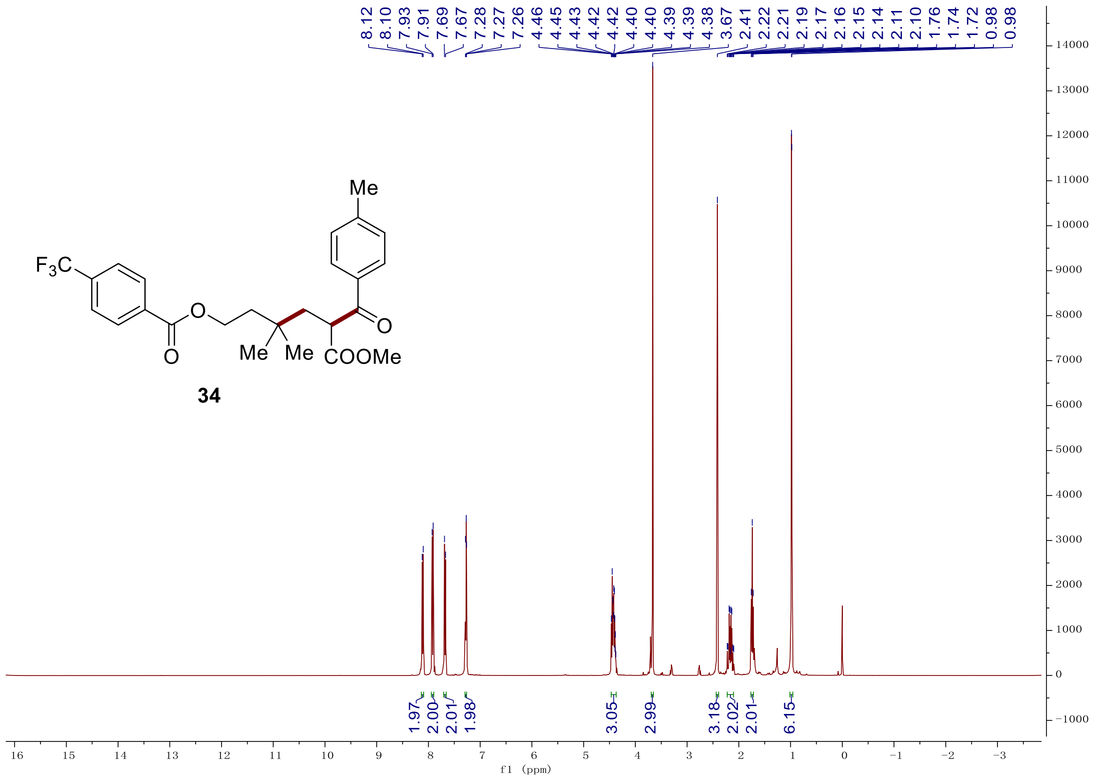

Figure S63. ^{1}H NMR of 34

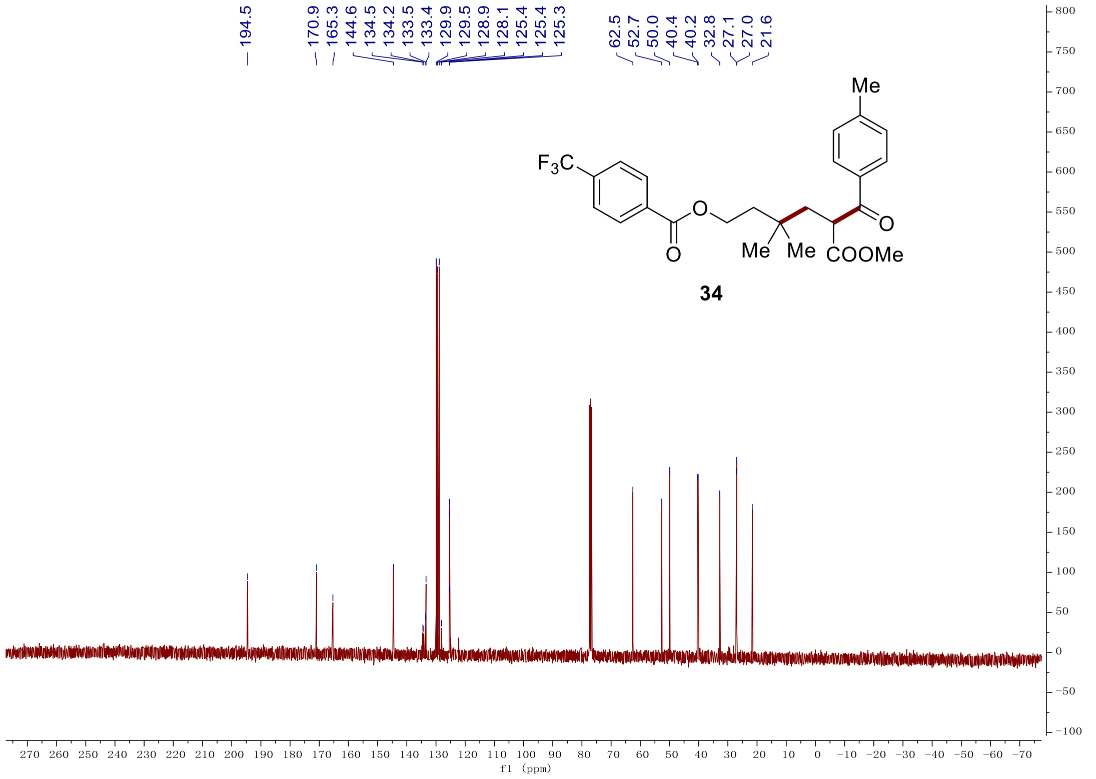

Figure S64. ^{13}C NMR of 34

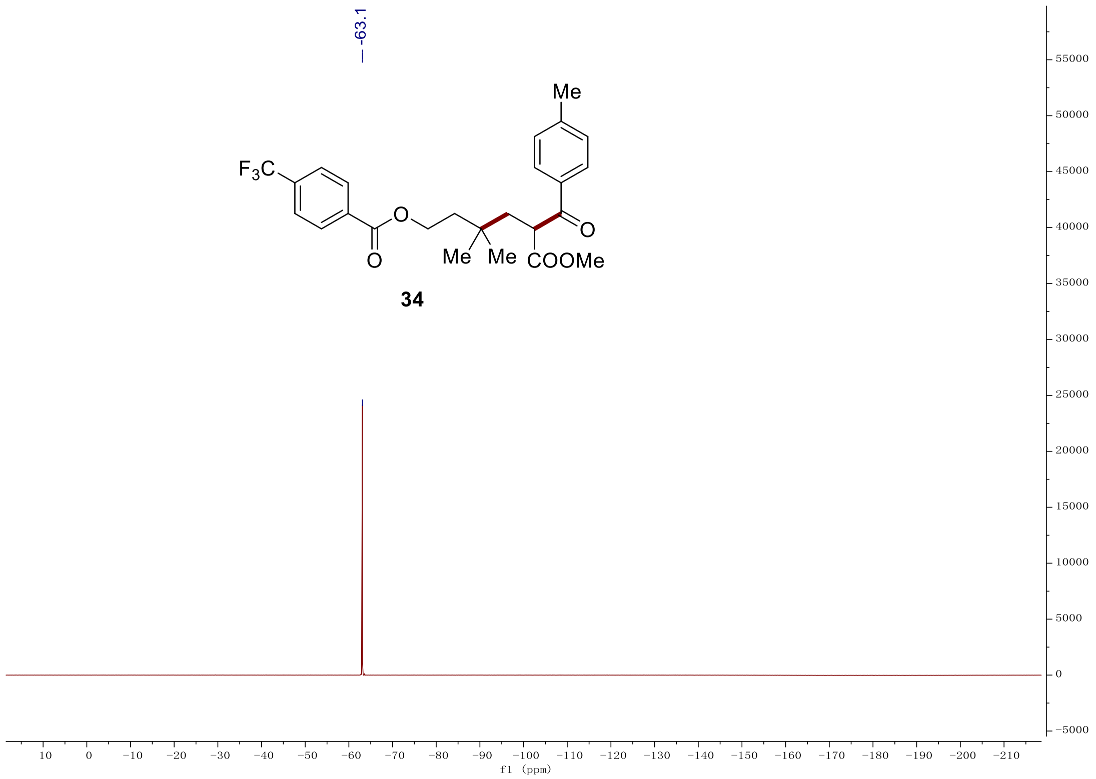

Figure S65. ^{19}F NMR of 34

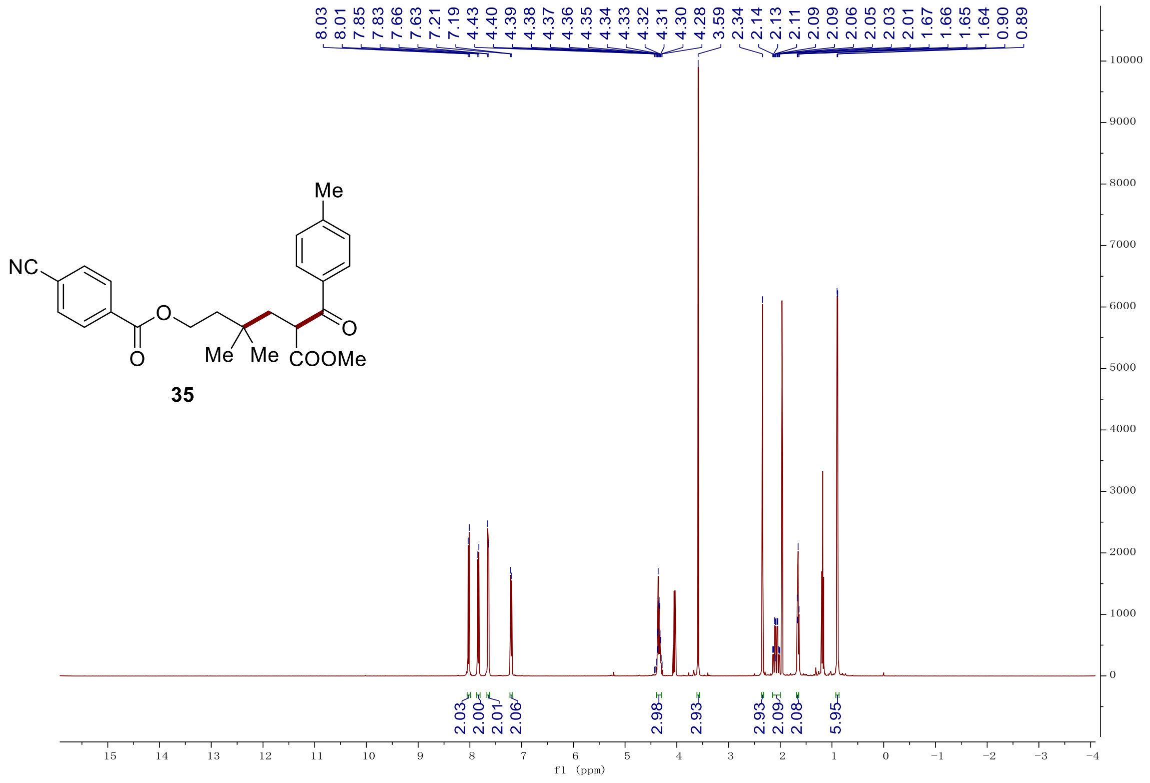

Figure S66. ^{1}H NMR of 35

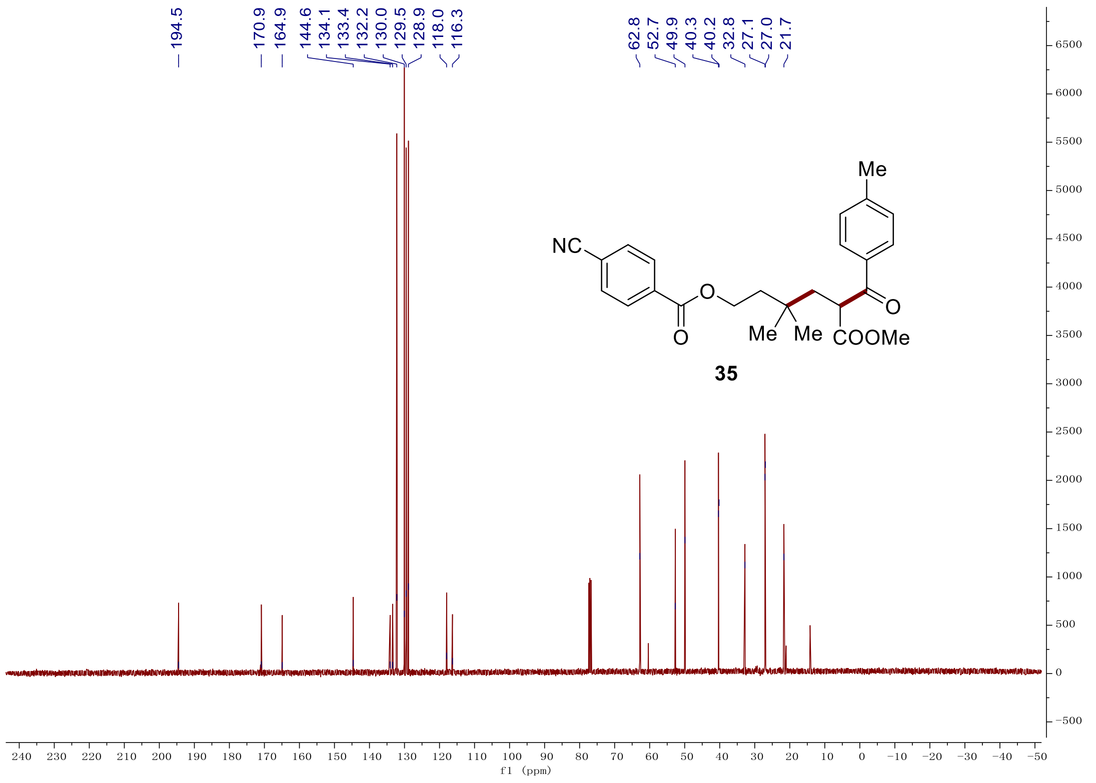

Figure S67. ^{13}C NMR of 35

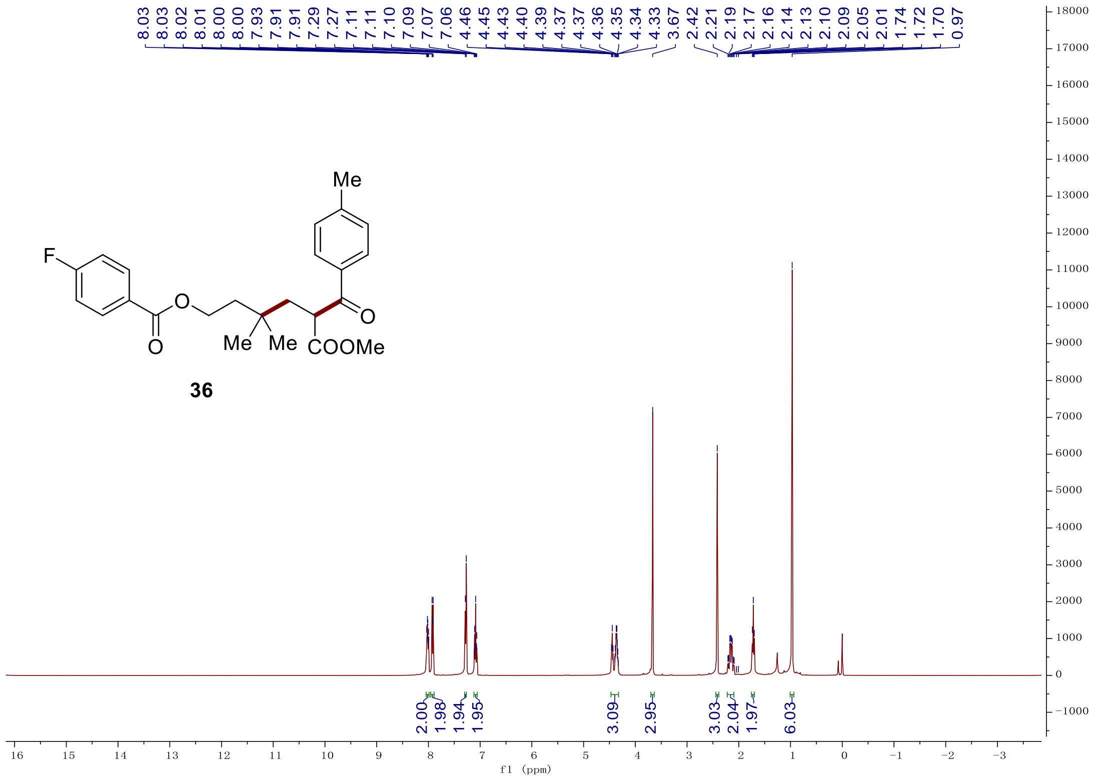

Figure S68. ^{1}**H NMR of 36**

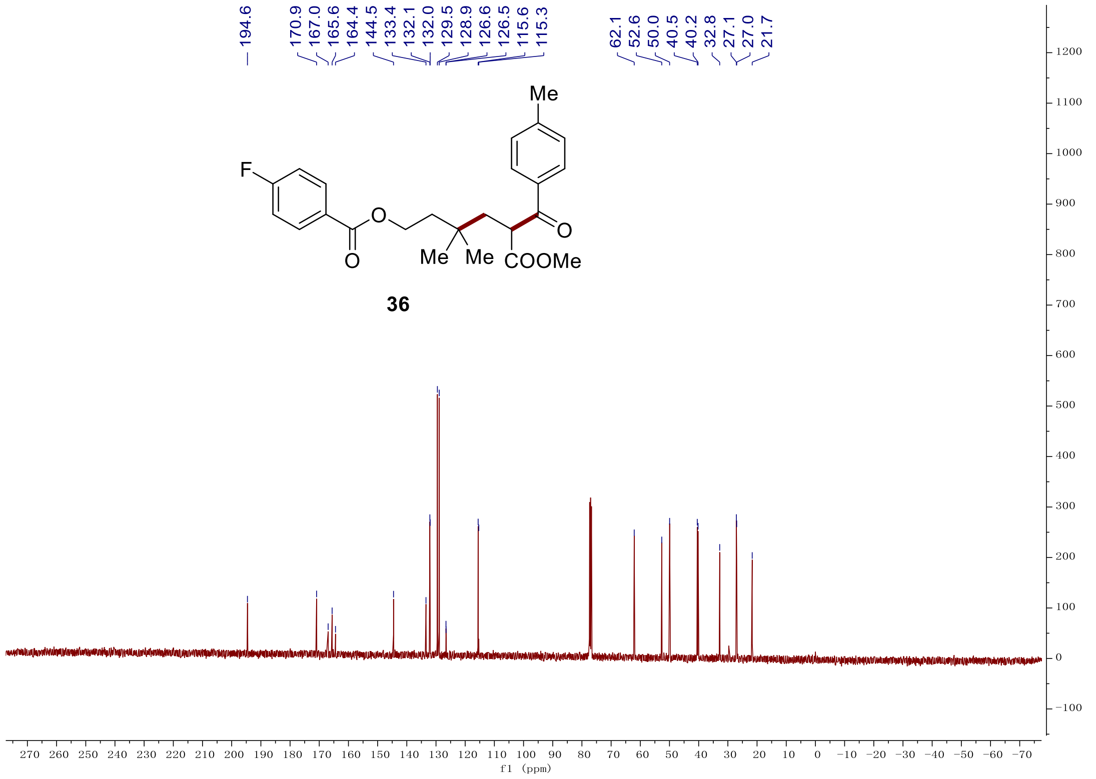

Figure S69. ^{13}**C NMR of 36**

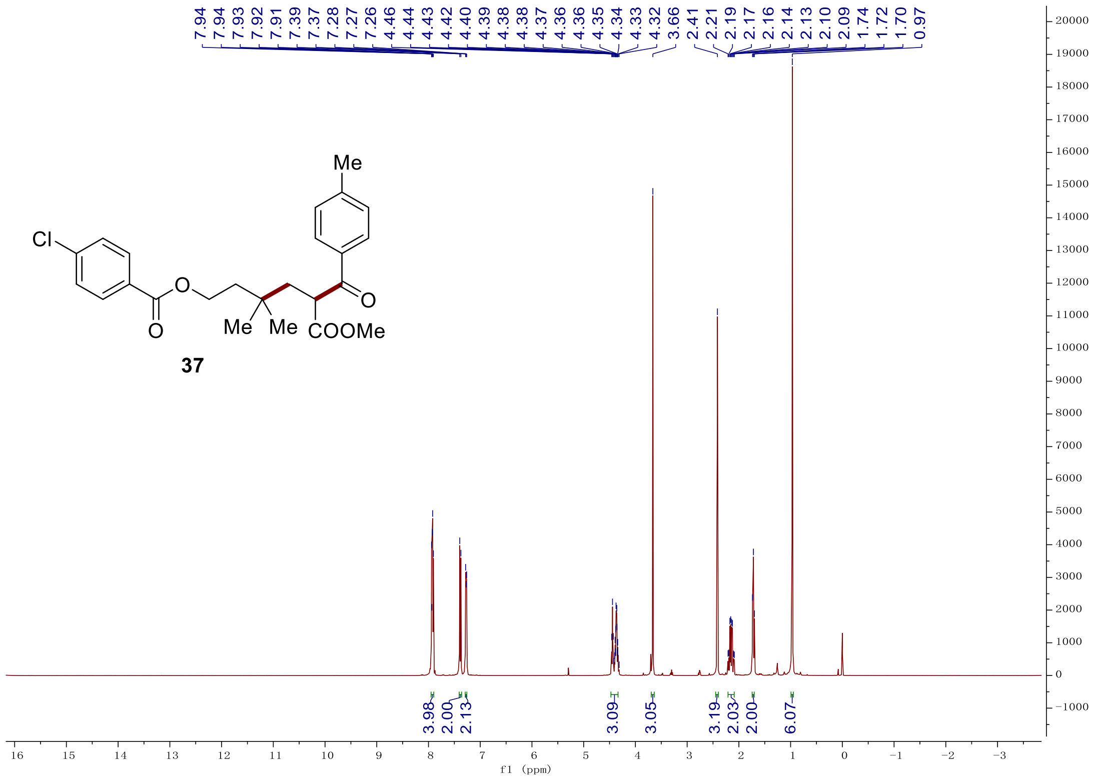

Figure S70. ^{1}H NMR of 37

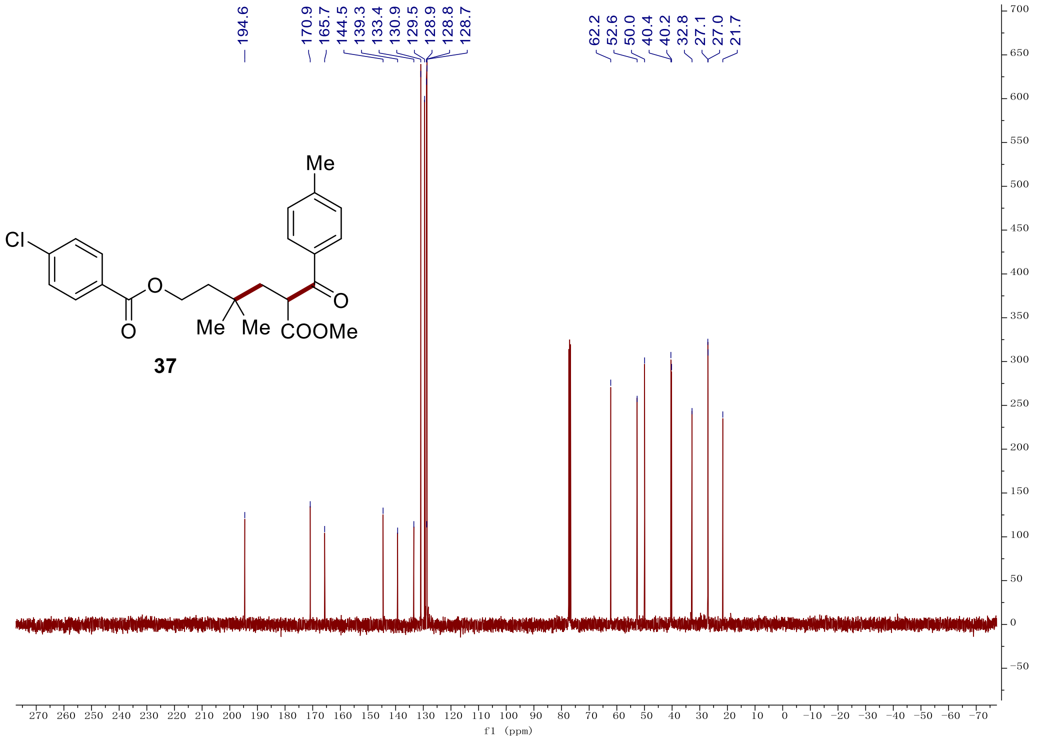

Figure S71. ^{13}C NMR of 37

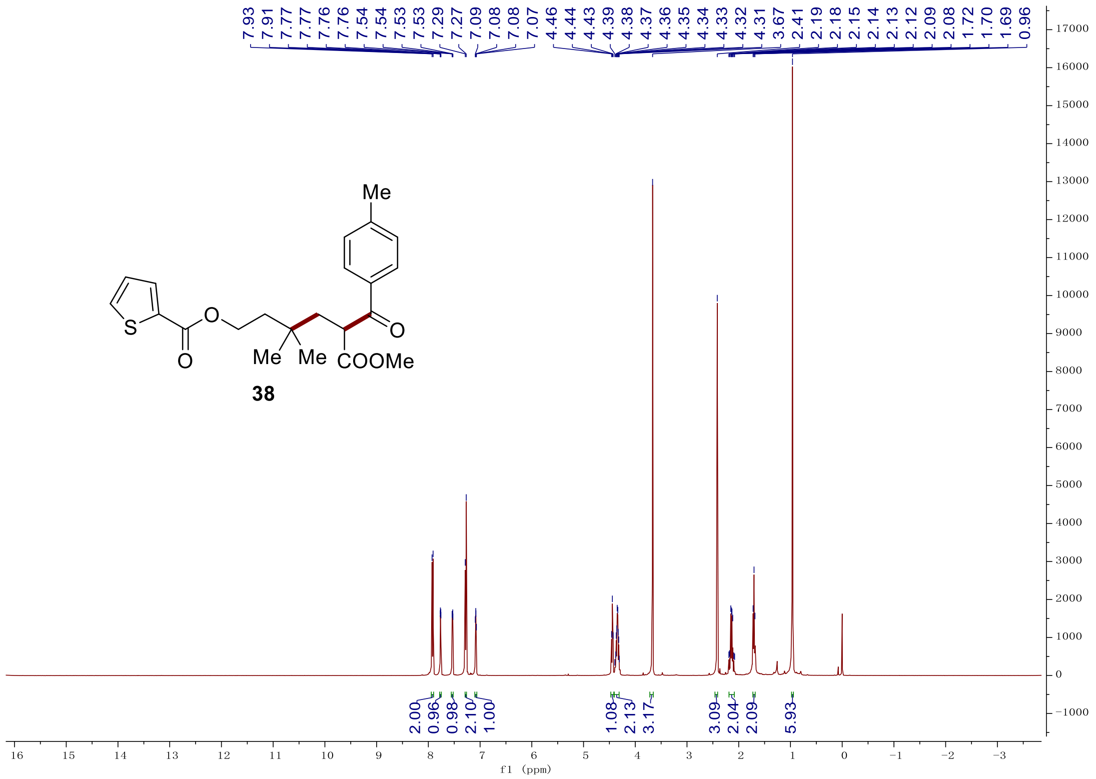

Figure S72. ^{1}H NMR of 38

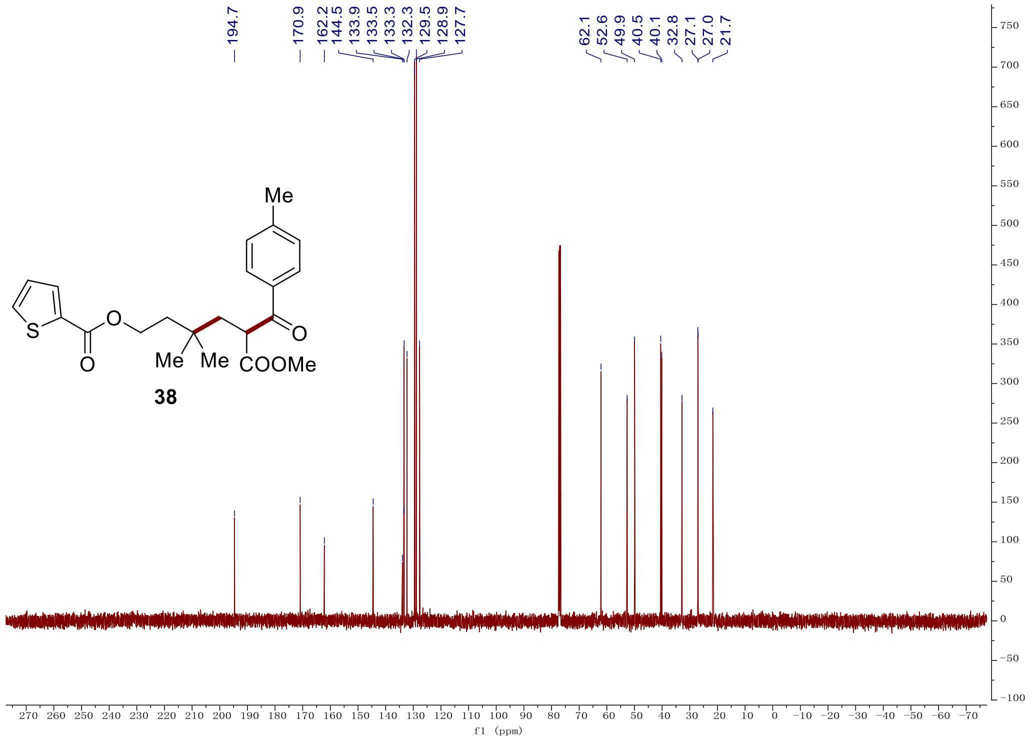

Figure S73. ^{13}C NMR of 38

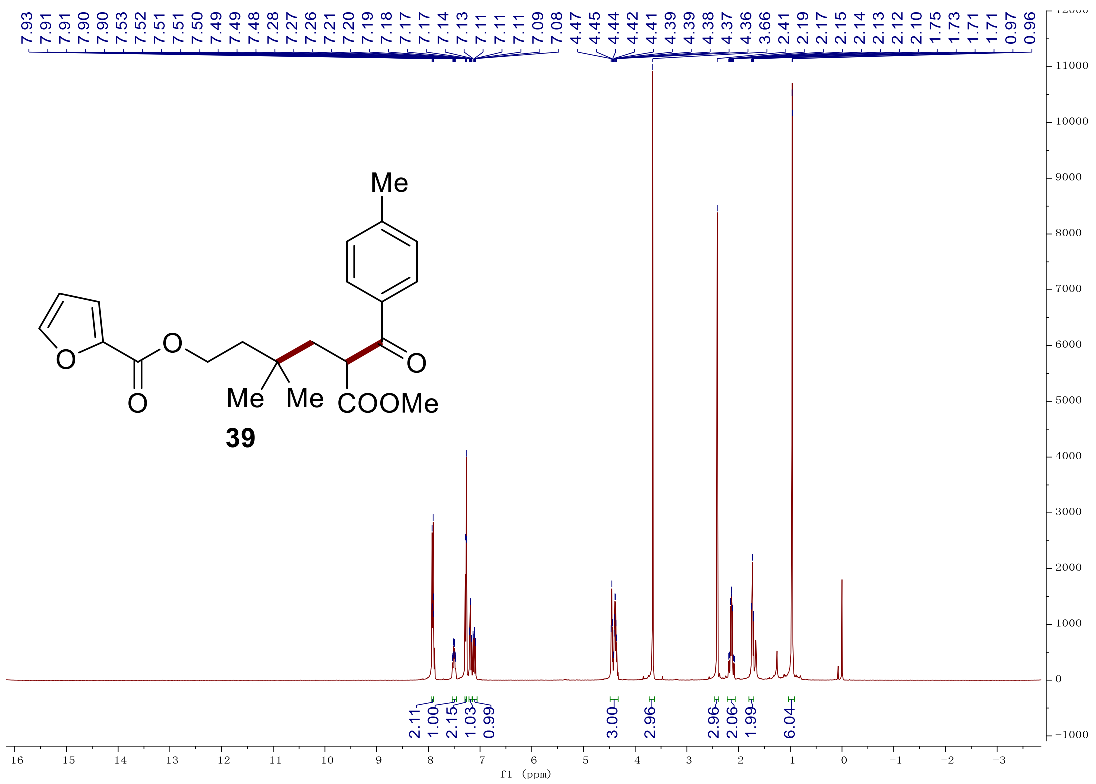

Figure S74. ^{1}H NMR of 39

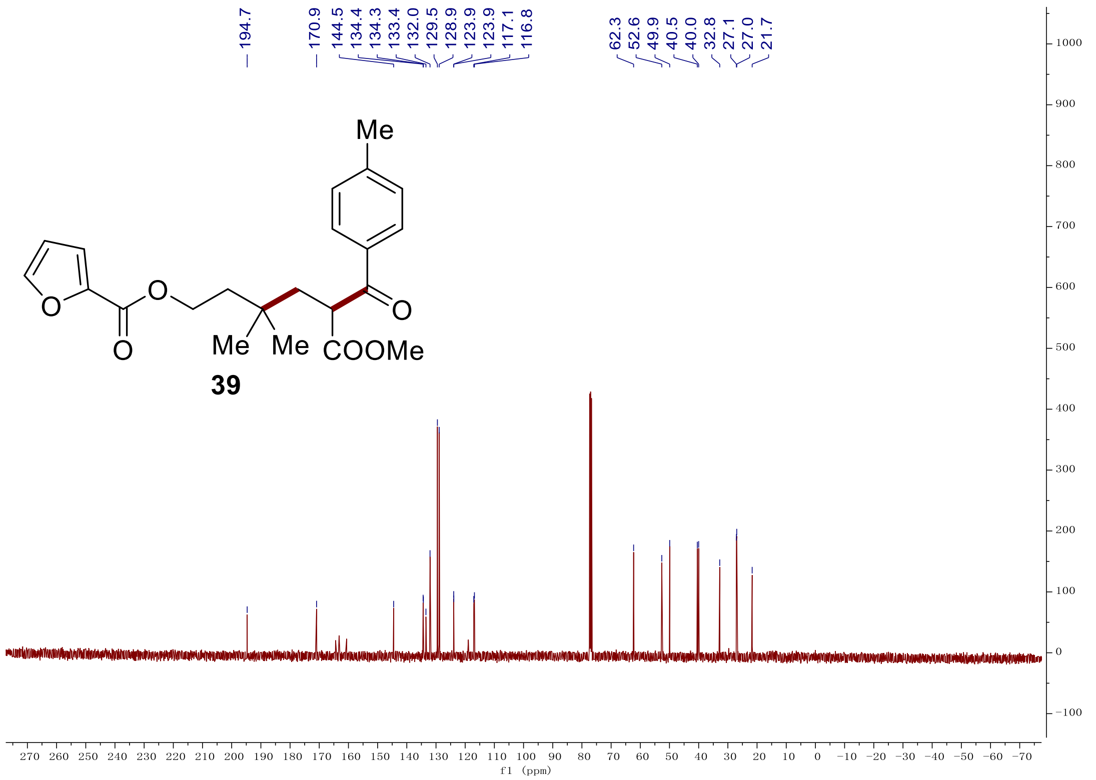

Figure S75. ^{13}C NMR of 39

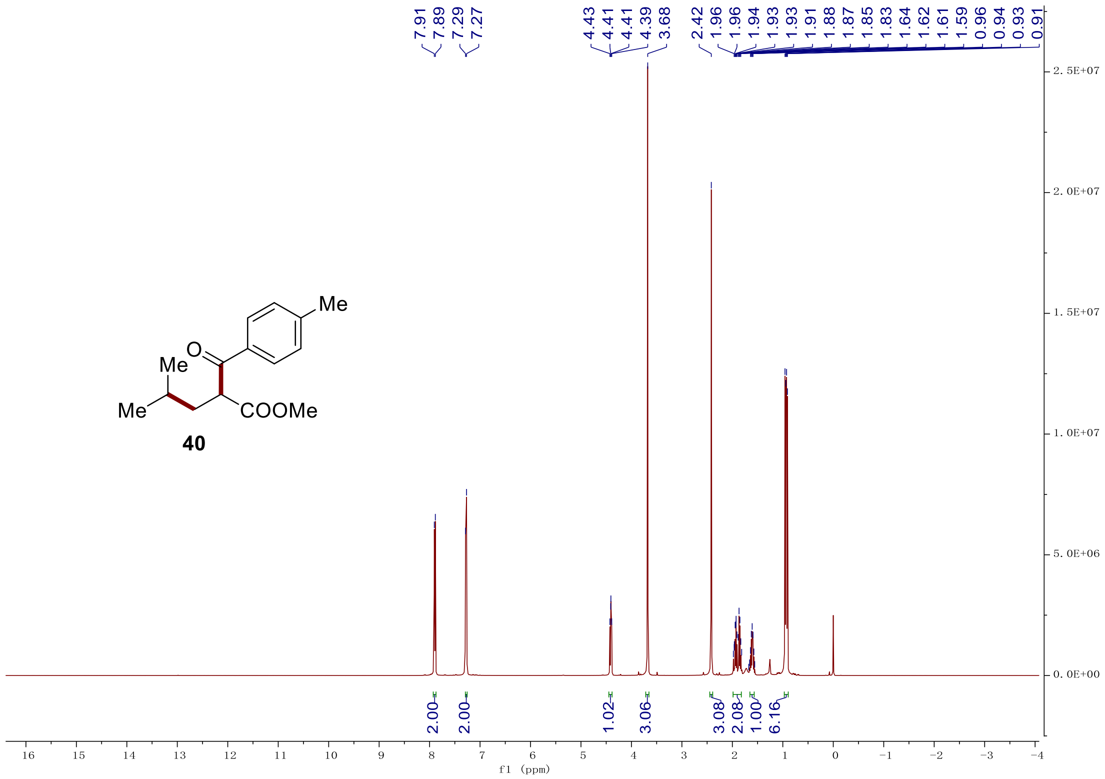

Figure S76. ^{1}H NMR of 40

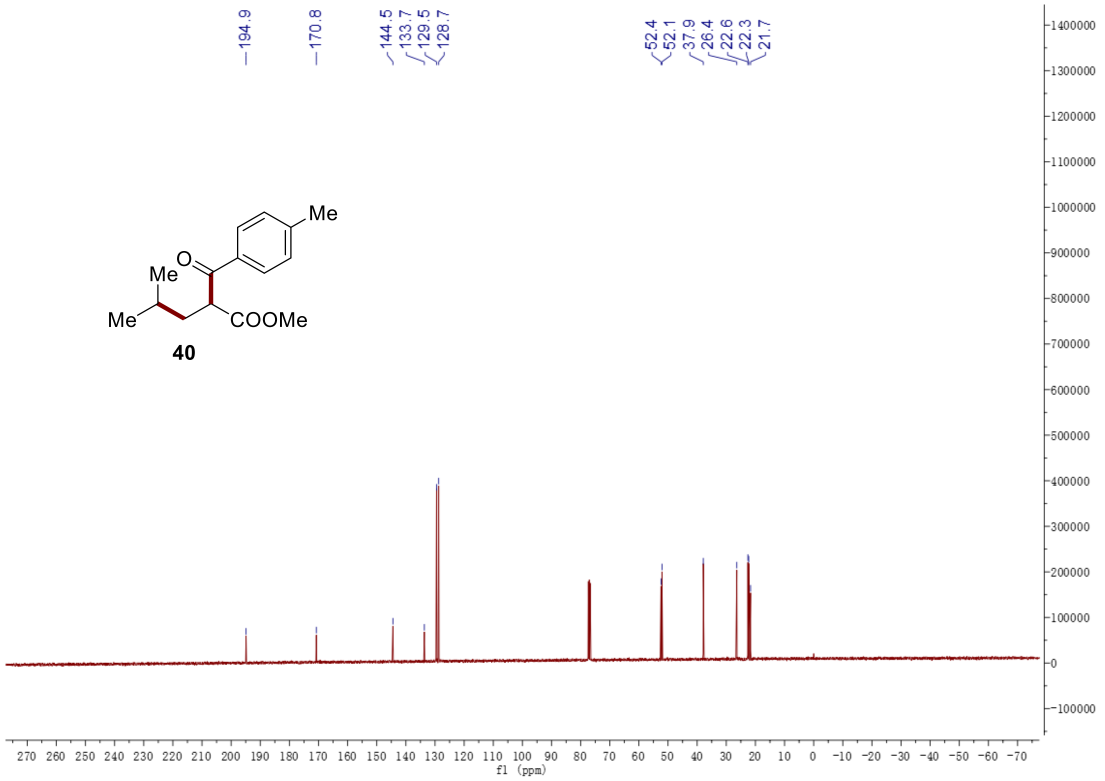

Figure S77. ^{13}C NMR of 40

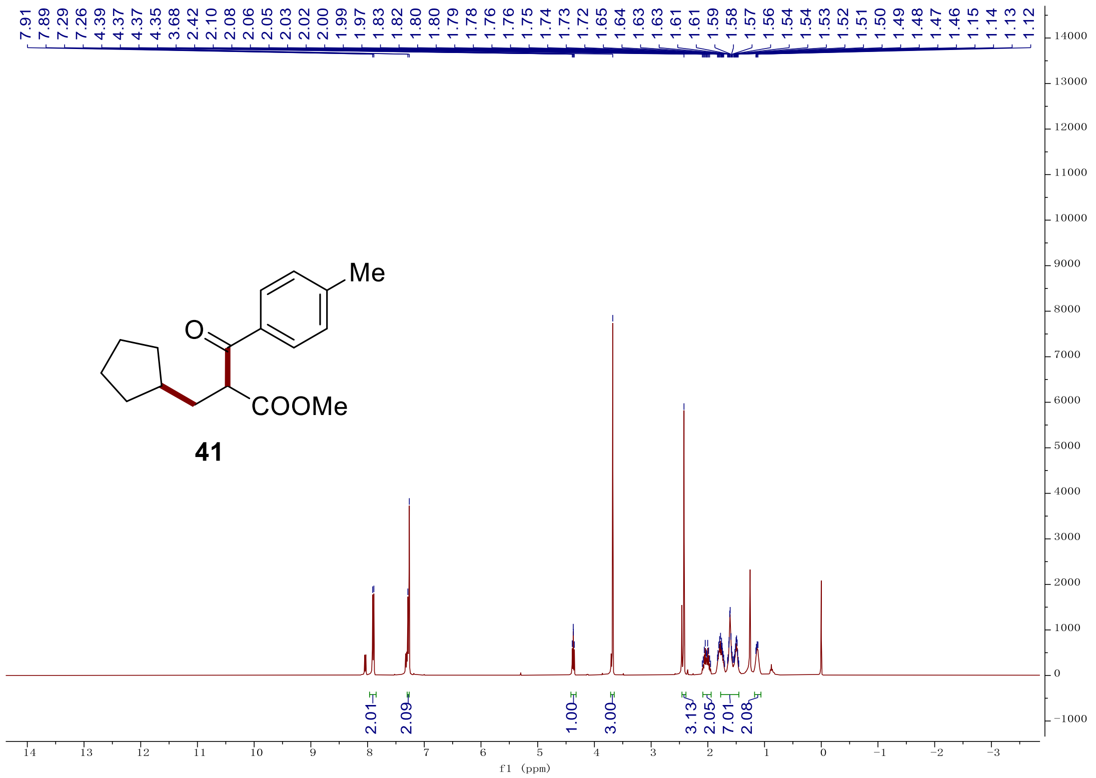

Figure S78. ^{1}H NMR of 41

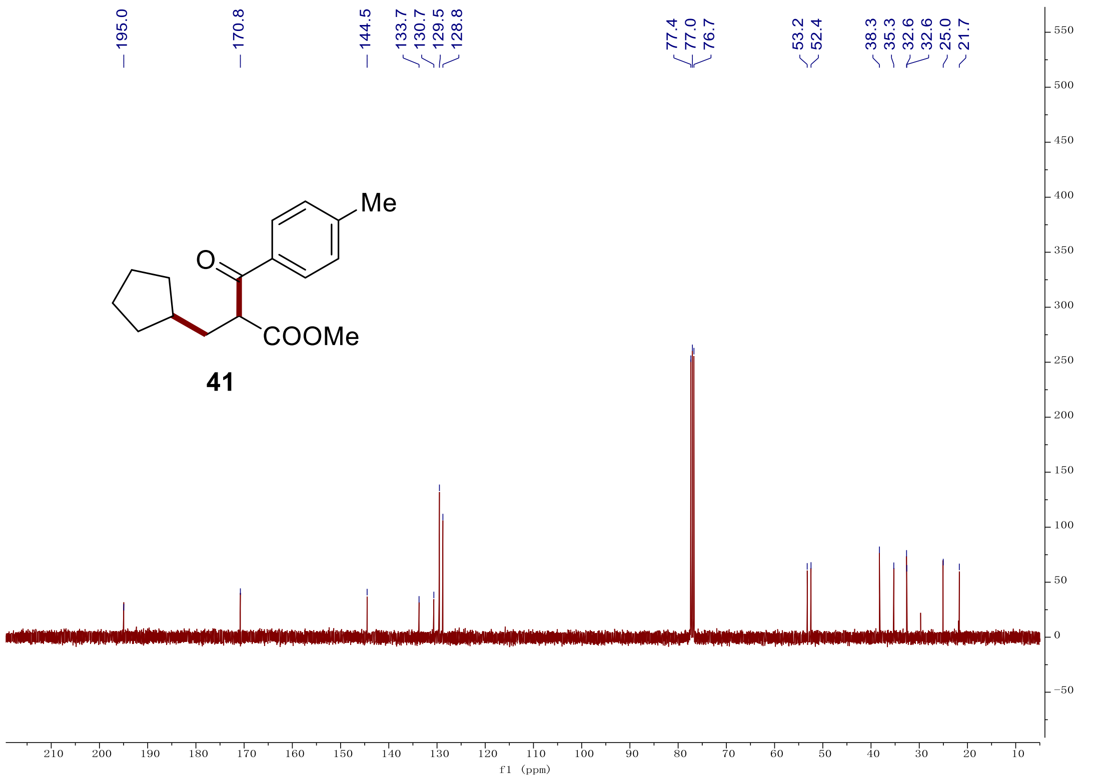

Figure S79. ^{13}C NMR of 41

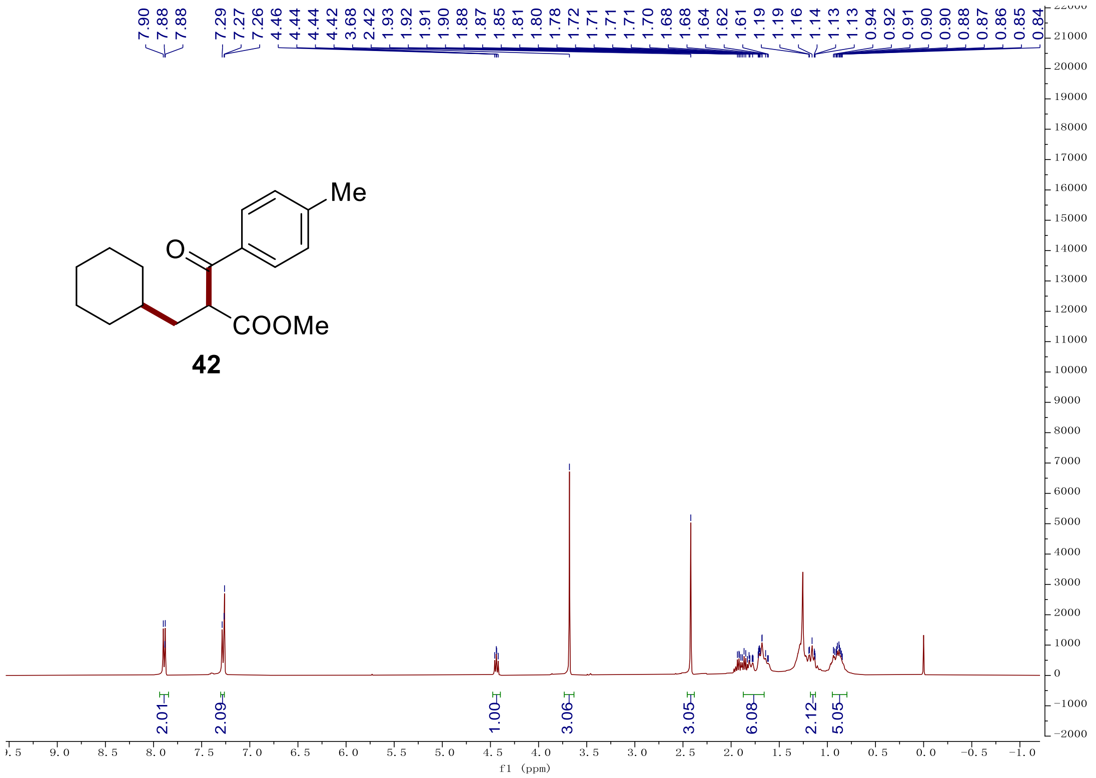

Figure S80. ^{1}H NMR of 42

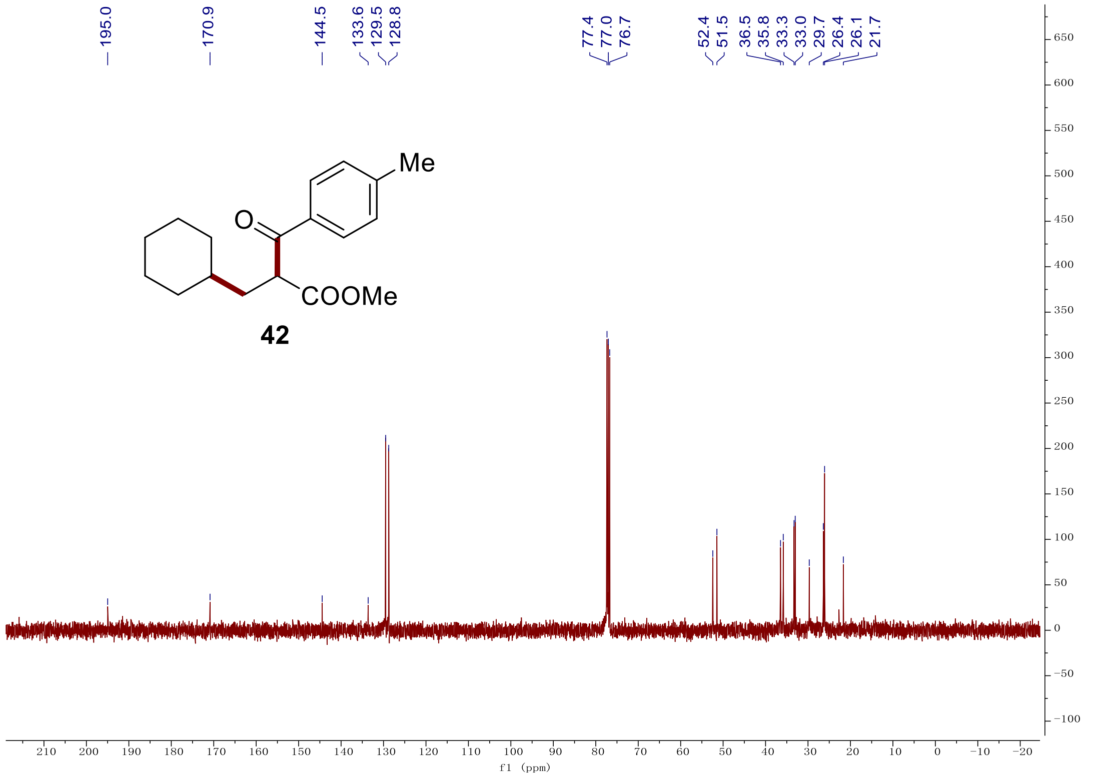

Figure S81. ^{13}C NMR of 42

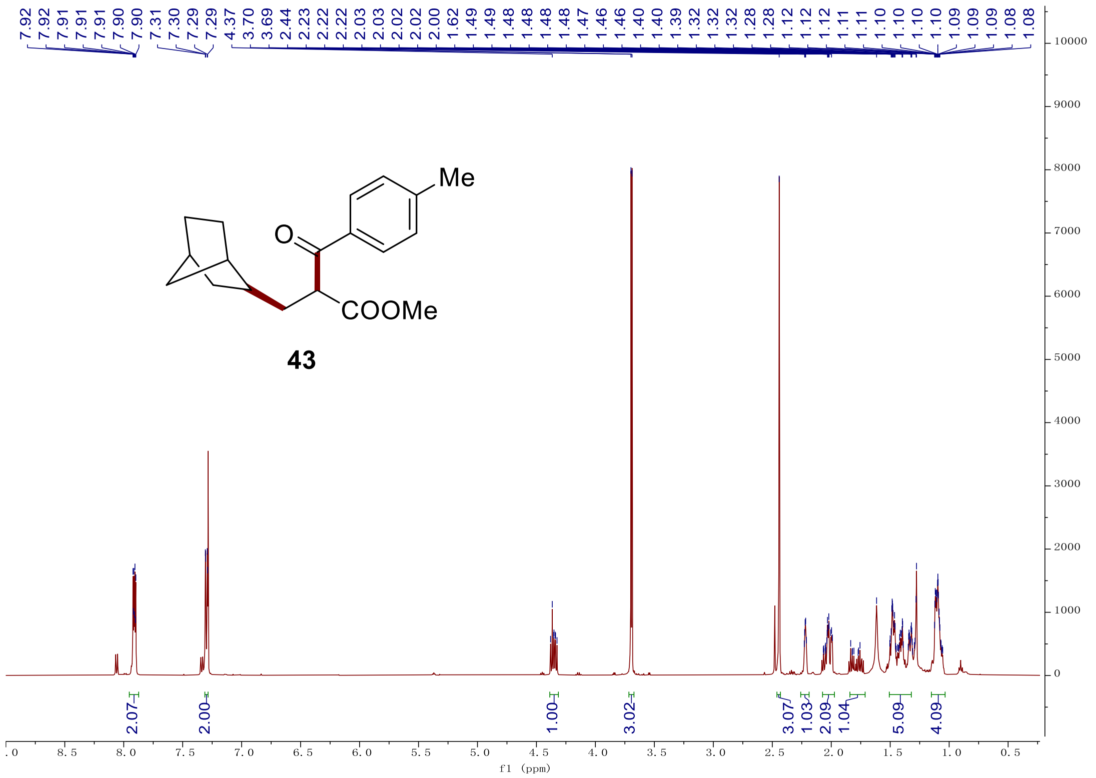

Figure S82. ^{1}H **NMR of 43**

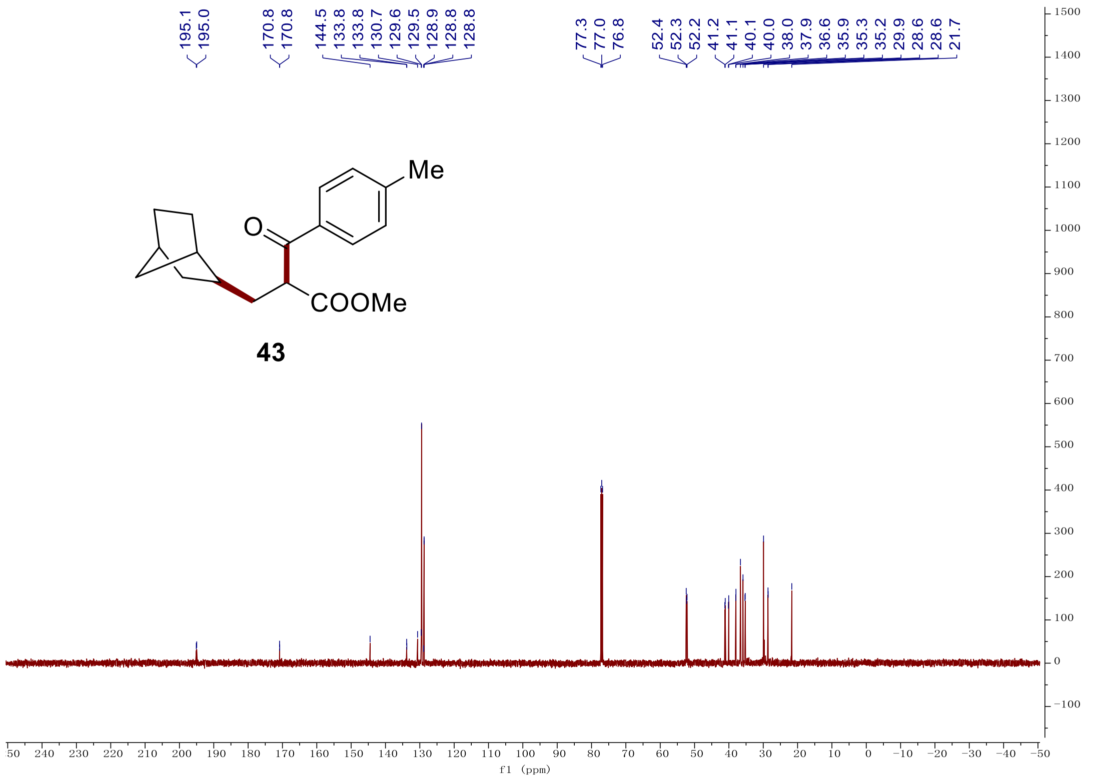

Figure S83. ^{13}C **NMR of 43**
